# Targeting Cdc42 improves motor phenotype in Parkinson’s disease mice and reveals age-dependent susceptibility to α-synuclein

**DOI:** 10.1016/j.isci.2025.114217

**Published:** 2025-11-25

**Authors:** Verena Bopp, Jaehyun LeeBae, Patrick Oeckl, Julia K. Kühlwein, Veselin Grozdanov, Martin Kiechle, Benjamin Mayer, Bettina Möhrle, Hartmut Geiger, Karin M. Danzer

**Affiliations:** 1 Department of Neurology, University Clinic Ulm, 89081 Ulm, Germany; 2 German Center for Neurodegenerative Diseases (DZNE), 89081 Ulm, Germany; 3 Institute of Epidemiology and Medical Biometry, Ulm University, 89075 Ulm, Germany; 4 Institute of Molecular Medicine, Ulm University, 89081 Ulm, Germany

**Keywords:** Molecular biology, Neuroscience, Transcriptomics, Model organism

## Abstract

Aging and accumulation of α-synuclein (α-syn) oligomers in the brain are indisputably linked to Parkinson’s disease (PD). Using an inducible α-syn oligomer mouse model, we demonstrate that the induction of PD-associated α-syn oligomers for the same time span caused PD-associated symptoms only in aged, but not in young mice. Biochemical studies revealed that α-syn oligomer formation precedes motor decline, with age and α-syn expression jointly determining the motor phenotype. Single-nucleus RNA sequencing (snRNA-seq) identified a PD-related transcriptional signature in basal ganglia neurons (BGNs), which overlapped in part with aging-associated changes. Short-term pharmacological inhibition of the small RhoGTPase CDC42 in aged, symptomatic animals improved motor function without reducing oligomer levels. These findings indicate that aging processes strongly influence the susceptibility to PD-like symptoms and that targeting age-related pathways, rather than α-syn oligomer burden alone, may provide effective strategies to improve outcomes in PD.

## Highlights

• Aging increases vulnerability to α-synuclein oligomer-induced motor decline
• Age-related pathways, not oligomer burden, shape susceptibility to α-synuclein oligomers
• Aging and α-synuclein effects intersect on proteasomal and actin systems
• Cdc42 inhibition by CASIN alleviates motoric PD phenotype

## Graphical abstract

**Figure:**
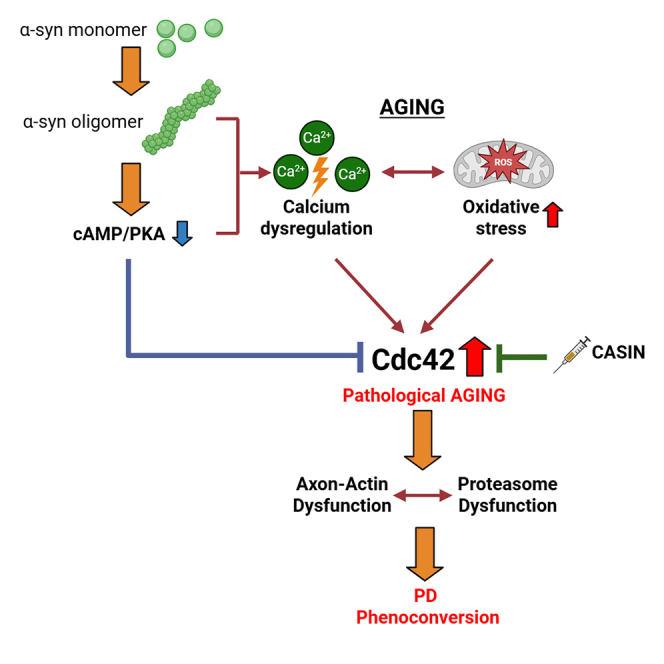

## Introduction

Aging is the major risk factor for Parkinson’s disease (PD). The onset of symptoms typically occurs between the ages of 55 and 65 years.^1^ The symptoms include not only motor disturbances but also non-motoric symptoms like sleep disturbances and cognitive impairment. The severity of symptoms tends to worsen with age. However, the age at disease onset can vary by decades, and this variability in age of symptom onset significantly influences the progression of the disease.^2^

One of the central molecular players implicated in the pathogenesis of PD is α-synuclein (α-syn), a presynaptic neuronal protein that is crucial for synaptic vesicle regulation. Under pathological conditions, α-syn misfolds and aggregates into oligomeric and fibrillar forms, and will by this means contribute to the neurodegenerative processes observed in PD.^3^ The accumulation of α-syn aggregates in mouse models for PD is increased in aged mice,^4^^,^^5^ and there is more α-syn in the soma of neurons of the substantia nigra pars compacta in aged humans.^6^ A range of symptoms that are associated with PD, including motor and cognitive symptoms, may also arise due to aging and co-pathologies.

A central concept of geroscience is that the biological processes of aging drive the onset of aging-associated diseases. Whether molecular and cellular processes that underlay aging contribute causatively to PD initiation, though, is still not known.^7^ We still cannot answer the fundamental question, whether the aged organism is more vulnerable to a disease-triggering event, like α-syn aggregation, so that the disease develops primarily only upon aging, or whether, for example, α-syn oligomer deposits simply require a distinct, long time to develop into disease phenotypes. The second scenario would mean that only the time span is important and thus independent of molecular or cellular changes that are associated with normal aging.

Aging at the cellular level refers to the progressive decline in the physiological function of cells, leading to reduced capacity for repair, replication, and response to stress. This decline is influenced by various interconnected biological processes and mechanisms. In addition to the progressive accumulation of molecular damage inside cells, aging is associated with an overall decrease in proteasome activity, impaired autophagy, mitochondrial dysfunction, rearrangements of the cytoskeleton, and neuroinflammation, which are precisely the signaling pathways that are also dysregulated in neurodegenerative disease.^8^ Additionally, aging primarily leads to impairments of intracellular clearance mechanisms, especially in the ventral substantia nigra, which in turn increases vulnerability to neurodegeneration. An age-related association between the increase in α-syn and the decrease in nigral tyrosine hydroxylase has been demonstrated.^6^

In recent years, advanced methodologies such as single-cell sequencing have facilitated a more detailed analysis of different cell types and states during disease progression and aging. This has opened potential avenues for combating PD,^9^ provided detailed resources for various cell types affected by PD or aging,^7^^,^^10^ and led to the discovery of disease- or age-relevant rare cell types.^11^

In this study, we utilized a well-characterized α-syn oligomer PD mouse model^5^ to determine the age at which induction of α-syn oligomerization results in PD phenotypes. We further dissect α-syn oligomer and motor phenotypes in relation to time span and aging and study on a biochemical and single-cell transcriptomics level which cellular pathways fuel PD disease development and/or aging.

## Results

**Figure 1:**
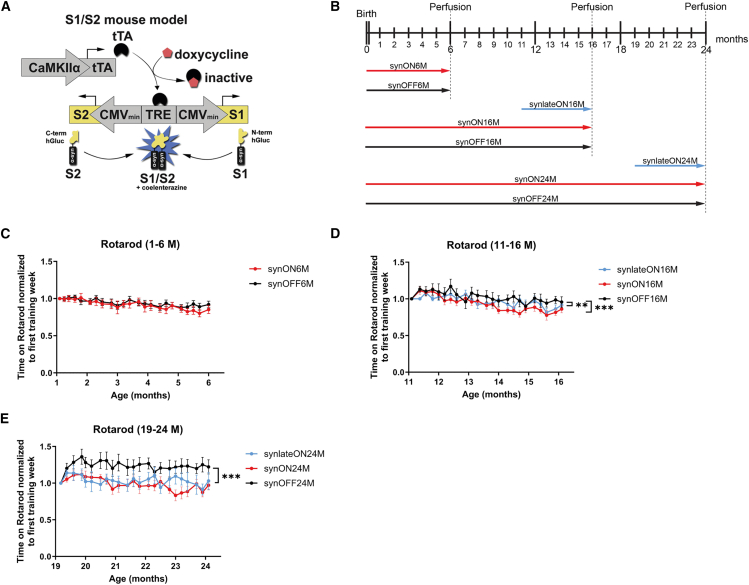
S1/S2 PD mouse model shows age and α-syn-related motoric deficits (A) Schematic principle of the S1/S2 PD mouse model, which includes a protein complementation assay expressing α-syn coupled to either the N- or C-terminal part of a Gaussia luciferase. The expression takes place under the neuron-specific CamKIIα promoter and is modeled as a Tet-off system driven by doxycycline. Figure modified from Kiechle et al., 2019. (B) Overview of all different S1/S2 mouse cohorts that differ in their age (6, 16, and 24 M) and length of S1/S2 expression (short, long, and non-expressing). (C–E) Accelerating Rotarod was used to determine motor balance and coordination of all S1/S2 mouse cohorts, comparing short-, long-, and non-expressing animals (C) 1–6 M, (D) 11–16 M, and (E) 19–24 M of age by measuring the latency to fall in seconds (s) (*n* = 12 male and female mice, repeated measures two-way ANOVA, ∗∗∗*p* < 0.001, ∗∗*p* < 0.01; data: mean ± SEM).

For the onset of PD, aging is the major risk factor. We have previously described a transgenic α-syn PD mouse model in which the time-dependent accumulation of α-syn oligomers upon aging of the mice was accompanied by neuronal cell loss and diminished motoric abilities.^5^ The question whether these motoric impairments were caused by a combination of age-related changes and α-syn oligomer expression, or simply by a time span of α-syn oligomer exposure, could not be determined. We, therefore, used an inducible PD mouse model based on a human α-syn protein complementation system expressing α-syn fused to halves of Gaussia luciferase (called S1/S2 model). The expression of this non-bioluminescent S1/S2 construct is neuron specific and inducible. S1/S2 fragments are reconstituted when brought together by S1-S2 interactions (Tet Off System) (Figure 1A).

To determine a potential vulnerable age for S1/S2 oligomer formation and/or motoric decline, we induced S1/S2 oligomer expression for only 5 months (M) at 11 and 19 M of age (synlateON16M and synlateON24M) and performed Rotarod testing. These groups were compared to mice with the same age and lifelong S1/S2 expression (synON) or no expression at all (synOFF) at the ages of 6, 16, and 24 M (Figure 1B). We also compared these experimental S1/S2 mouse groups that differ in their age (Figure 1B). Young synON and synOFF mice between 1 and 6 M of age showed no significant differences in their motoric abilities (Figure 1C). In contrast, mice expressing S1/S2 for only 5 M starting at 11 M of age (synlateON16M) as well as mice expressing S1/S2 throughout their entire lives (synON16M) exhibited significantly reduced motor performance between 11 and 16 M of age compared to control animals (synOFF16M) (Figure 1D). The progressive decline in motoric abilities became even more pronounced between 19 and 24 M of age in the S1/S2-expressing animals in which expression was only induced at 19 M of age (synlateON24M) or throughout their entire lives (synON24M) compared to controls (Figure 1E). Notably, at 24 M of age, there was no difference in the motoric phenotype between the synlateON and synON condition. These results suggest that age, rather than the duration of oligomer exposure, determines the severity of the S1/S2-induced motoric phenotype with synlateON24M and synON24M animals showing the most significant motoric impairment compared to controls.

**Figure 2:**
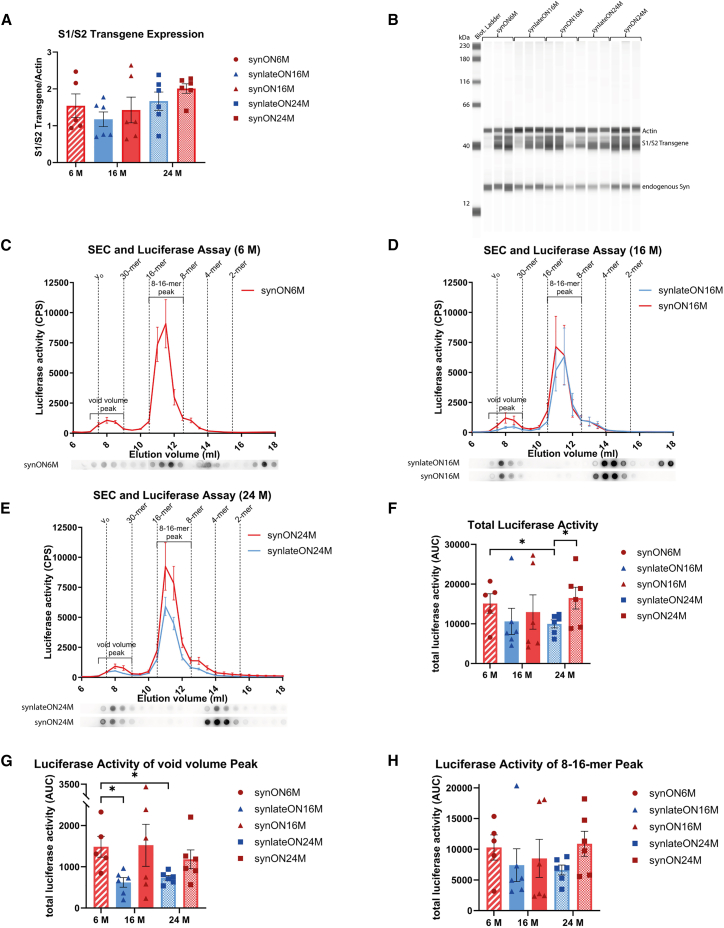
S1/S2 expression is similar across age groups (A and B) Determination of S1/S2 transgene and endogenous α-syn expression of full-brain lysates from S1/S2 mice by capillary-based western blot analysis (simple western) using the Jess system and normalization to Actin (anti-α-syn Syn1 antibody and anti-Actin antibody) (Student’s *t* test; data: mean ± SEM). (C–E) Gaussia luciferase activity and dot blot analysis (anti-α-syn 15G7 antibody) of SEC fractions of full-brain lysates from S1/S2 mice animals of age (C) 6 M, (D) 16 M, and (E) 24 M. The graphs illustrate the mean ± SEM of n = 5–6 animals per group. There is a clear enrichment of 8- to 16-mer species in all mice groups. (F–H) Quantification of the AUC of respective total luciferase activity of SEC fractions (one-way ANOVA with Tukey’s post hoc test, ∗*p* < 0.05; data: mean ± SEM).

To determine whether the motoric alterations correlate with the total amount of expressed S1/S2, we performed capillary-based simple western with anti-α-syn antibody. While the expression levels of S1/S2 between the groups slightly varied, none of the comparisons reached statistical significance (Figures 2A and 2B). To gain insight into the various oligomer species, we performed size-exclusion chromatography (SEC) with subsequent luciferase activity measurement in each fraction. Short-expressing mice at the age of 16 M (synlateON16M) revealed a similar heterogeneous profile of S1/S2 oligomers as S1/S2-expressing mice from birth (synON16M) (Figure 2D). In contrast 24-M-old mice differed in their oligomer profile depending on whether S1/S2 oligomers were present since birth (synON24M) or only for 5 M (synlateON24M). This was also reflected in the total luciferase activity with a significantly higher area under the curve (AUC) for the lifelong expressing animals at 24 M of age (Figure 2F). A detailed analysis of the SEC profile separating the profile according to the two main peaks (void volume peak with sizes >430 kDa and a peak reflecting 8- to 16-mers, Figures 2G and 2H) revealed that 8- to 16-mers S1/S2 oligomers were enriched in synON24 animals compared to synlateON24 mice. Additionally, the void volume peak, containing aggregates larger than 430 kDa, showed a tendency to increase for the synON24M animals compared to synlateON24M (Figures 2G and 2H).

Since the motoric impairment in PD mice was absent at 6 M, we speculated that a lower level of S1/S2 oligomers would be present in this age group. However, contrary to our assumption, 6-M-old S1/S2-expressing mice already exhibited a similar S1/S2 oligomer profile to that of 16- or 24-M-old PD mice (Figure 2C). Together, these results suggest that S1/S2 oligomer formation precedes motoric decline in PD mice, with the age of the PD mice mainly determining the motoric phenotype. In contrast, the duration and timing of S1/S2 expression mainly affect oligomer load.

**Figure 3:**
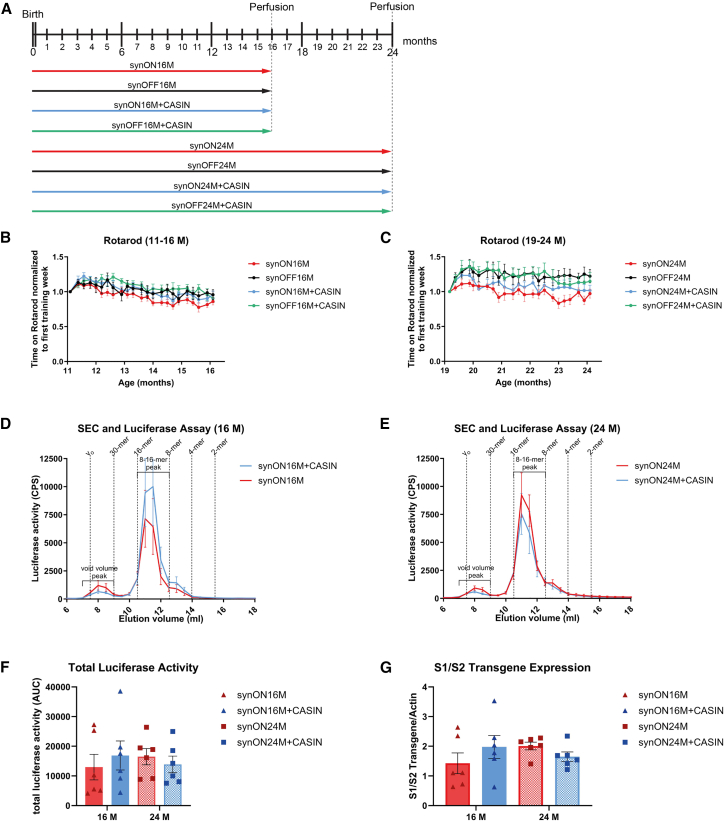
CASIN treatment alleviates motoric phenotype (A) Overview of the different S1/S2 mouse cohorts for CASIN treatment. They are categorized by age (16 and 24 M), levels of S1/S2 expression (ON vs. OFF), and treatment with CASIN. (B and C) Accelerating Rotarod was used to evaluate motor balance and coordination across S1/S2 mouse cohorts. The performance of expressing and non-expressing animals with and without CASIN treatment was compared, and the latency to fall (in seconds) was measured. Performance was assessed at (B) 11–16 M: significant differences were found between synON16M + CASIN vs. synOFF16M + CASIN (∗), synOFF16M + CASIN vs. synON16M (∗∗∗), synON16M + CASIN vs. synON16M (∗∗∗), and synOFF16M vs. synON16M (∗∗∗), and (C) 19–24 M: significant differences occurred between synON24M vs. synOFF24M (∗∗∗), synON24M vs. synON24M + CASIN (∗∗), synON24M vs. synOFF24M + CASIN (∗∗∗), synOFF24M vs. synON24M + CASIN (∗∗∗), and synON24M + CASIN vs. synOFF24M + CASIN (∗∗∗) (*n* = 12 male and female mice, repeated-measures two-way ANOVA, ∗*p* < 0.05, ∗∗*p* < 0.01, ∗∗∗*p* < 0.001; data: mean ± SEM). (D and E) Gaussia luciferase activity analysis was performed on SEC fractions from S1/S2 mice aged (D) 16 M and (E) 24 M. Comparisons included groups with and without S1/S2 expression, as well as with or without CASIN treatment (*n* = 6 male and female mice per group, data: mean ± SEM). (F) Quantification of the AUC for total luciferase activity in SEC fractions (one-way ANOVA with Tukey’s post hoc test; data: mean ± SEM). (G) Quantification of S1/S2 transgene expression in lysates from S1/S2 mice performed by capillary-based western blot analysis (simple western), normalized to Actin (Student’s *t* test; data: mean ± SEM).

The data demonstrated so far support that the biological processes of aging are associated to the onset of PD in the α-syn PD model. Aging has been associated with an elevated level of the activity of the small RhoGTPase cell division control protein 42 homolog (CDC42).^12^^,^^13^ This increase in CDC42 activity contributes to aging-associated phenotypes in multiple tissues and types of cells.^14^ Also in brain, there is significant increase in the activity of CDC42 upon aging.^12^ Moreover, an association of loss-of-function variants of a CDC42-activating gene *ITSN1* and PD has been found.^15^ Attenuation of CDC42 activity via the pharmacological compound CASIN (CDC42 activity specific inhibitor) in already aged mice increases lifespan.^16^ Attenuation of the activity of CDC42 targets several basic cellular and molecular pathways of aging including actin cytoskeleton remodeling, endocytosis, and several signaling pathways.^14^ We, therefore, tested whether CDC42 inhibition by CASIN might potentially rescue the PD-associated motoric decline in α-syn-expressing aged mice and administered it around the age of PD onset (12 M of age) and after age of onset (20 M of age). CASIN was administered in two rounds, each consisting of four consecutive daily doses with a 1-week interval between rounds, for a total of eight applications. Indeed, systemic treatment with CASIN (similar to the treatment regimen that results in lifespan extension, Figure 3A) ameliorated the motoric decline at the age 11–16 M, and was very pronounced at 21–24 M age range (Figures 3B and 3C). Interestingly, CASIN treatment did not affect the amount of α-syn oligomers. Aged mice treated with CASIN improved their motor function but had similar amounts of oligomers compared to aged, non-treated animals (Figures 3D–3G). These data suggest that while oligomers are necessary to develop PD symptoms, they are not sufficient, as confirmed by the presence of oligomers in young animals (Figure 2C). Our data indicate that a reduction of aging-associated changes is accompanied by improvements in PD-related phenotypes in the α-syn model, whereas α-syn oligomer levels remained unaffected. While these findings are consistent with the possibility that mitigating aging-related alterations contributes to the observed phenotypic improvements, the specific mechanisms underlying this relationship remain to be determined.

**Figure 4:**
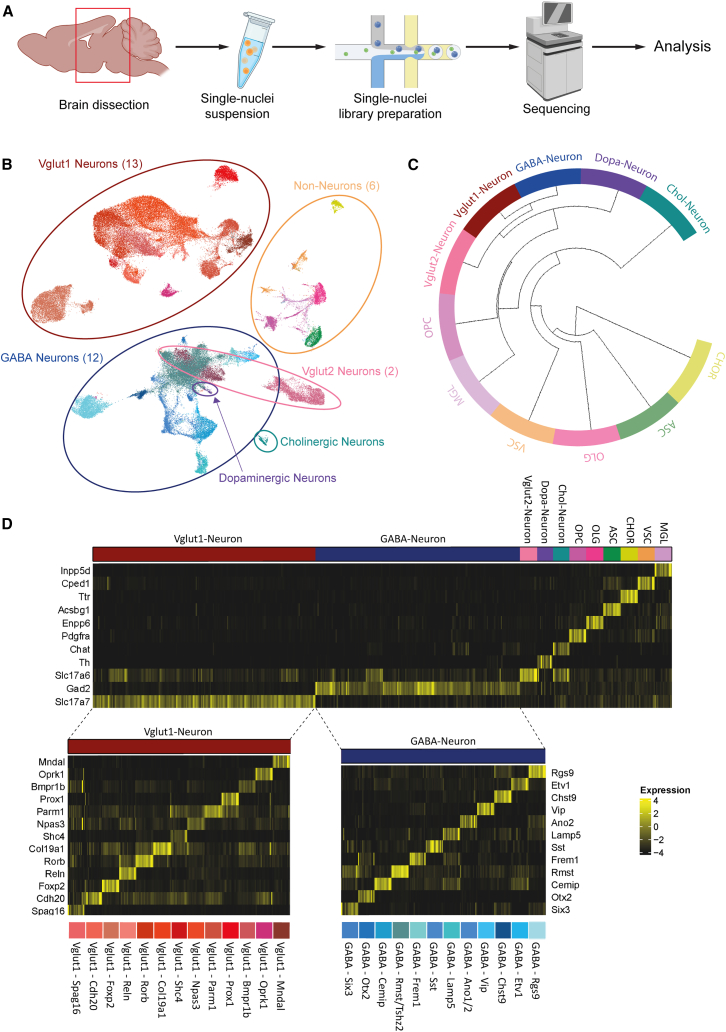
snRNA-seq enables cell-type-specific analysis of S1/S2 mice brains (A) Schematic workflow of the snRNA-seq approach. Nuclei were isolated from S1/S2 PD mice and used to generate gene expression libraries. Those were sequenced and proceeded to analysis (*n* = 4 animals per condition). Created with BioRender. (B) UMAP visualization of unbiased clustering with 105,689 quality-controlled cells. In total, 35 distinct cell types could be annotated. (C) Hierarchical clustering of the main cell types. Distances between non-neuronal and neuronal clusters were the largest. (D) Canonical marker genes and marker genes from mousebrain.org showcased clear differential expression patterns between clusters.

To further uncover molecular mechanisms driving PD pathogenesis upon aging, we performed single-nucleus RNA sequencing (snRNA-seq) on micro-dissected brains, using the 10× platform (Figure 4A). To this end, we isolated nuclei from brain slices of mice from 6, 16, and 24-M-old mice (synON6M, synOFF6M, synON16M, synlateON16M, synOFF16M, SynON24M, SynlateON24, SynOFF24M, *n* = 27 mice) with 2–3 replicate libraries from four animals for each genotype and condition. We obtained 105,689 high-quality nuclei for analysis (Figure S1). Unbiased clustering and cell type annotation based on known cell type-specific markers and marker genes from mousebrain.org^17^ (Table S1) identified 35 distinct cell types, including 12 inhibitory GABA neuronal clusters and 13 excitatory Vglut1 neuronal subclusters. Besides neuronal cell clusters, we also identified six non-neuronal clusters including Astrocytes (ASC), Oligodendrocytes (OLG), Oligodendrocyte progenitor cells (OPC), Microglia (MGL), Choroid Plexus (CHOR), and Vascular cells (VSC) (Figures 4B–4D).

**Figure 5:**
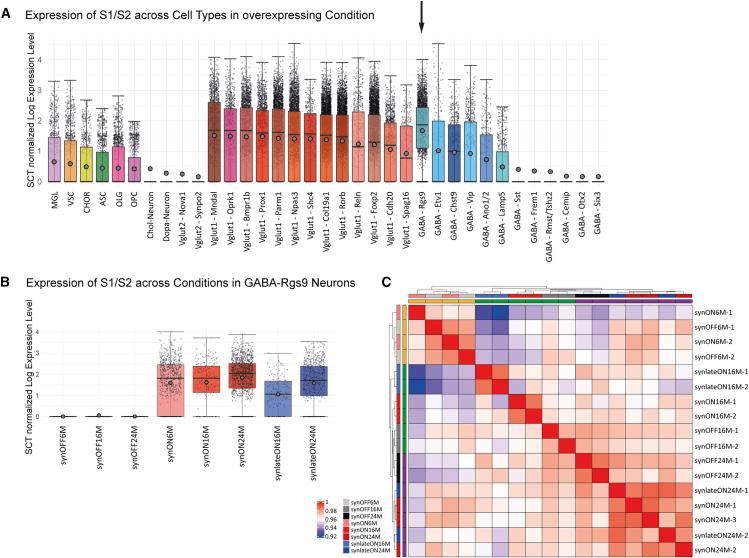
GABA-Rgs9 neurons exhibited higher levels of S1/S2 mRNA expression (A) Expression of S1/S2 transcript in S1/S2-expressing conditions across annotated cell types. GABA-Rgs9 neurons showed the highest overall expression of S1/S2. GABA-Rgs9 neurons also represent GABAergic BGNs (data: mean ± SD). (B) S1/S2 expression in GABA-Rgs9 neurons was comparable between the S1/S2-expressing conditions, while the synOFF conditions showcased near-zero expression levels (data: mean ± SD). (C) Similarity heatmap of all samples in GABA-Rgs9. (Euclidean distance with complete-linkage clustering). At 16 M, all samples were most similar to their replicates, but at 24 M, the synON and synlateON replicates formed a cluster together and at 6 M, even the synON and synOFF clustered together. Boxplots with interquartile range (whiskers) and mean average (dots).

To characterize the impact of the presence of S1/S2 oligomers on the distinct cell types, we assessed the relative abundance of S1/S2 transcripts across every cell type under all transgene-expressing conditions. We have chosen the neuronal cell type GABA-Rgs9 since this cell type exhibited the highest S1/S2 expression among all annotated cell types (Figure 5A). Further support for the choice of GABA-Rgs9 cluster comes from the fact that the GABA-Rgs9 cluster represents GABAergic Basal Ganglia Neurons (BGNs) based on transcriptional data markers from mousebrain.org (Table S1). Basal ganglia is a brain region crucial for motor control and central for motor impairments in PD. GABAergic neurons are important for movement regulated by forming inhibitory pathways within the basal ganglia. Additionally, GABA-Rgs9 cluster shows a high number of differentially expressed genes (DEGs) in PD mice (Figure S2). We, therefore, focused our further analysis on the GABA-Rgs9 cluster.

Within the GABA-Rgs9 cluster, we next examined the level of S1/S2 expression per cell across all conditions. We also performed a similarity analysis using all 11,706 detected genes in the GABA-Rgs9. The levels of S1/S2 transcripts were consistent among the transgene-expressing conditions, whereas the OFF conditions displayed, as expected, near-zero S1/S2 expression levels (Figure 5B). A similarity heatmap based on the expression of all genes revealed subclustering of synON24M and synlateON24M replicates at 24 M, a pattern not observed at 16 M. At 6 M, the synON and synOFF replicates clustered together, which also corresponds to their similar motor performance at this age (Figure 5C), as 16-M-old S1/S2 mice displayed only minor motor deficits.

**Figure 6:**
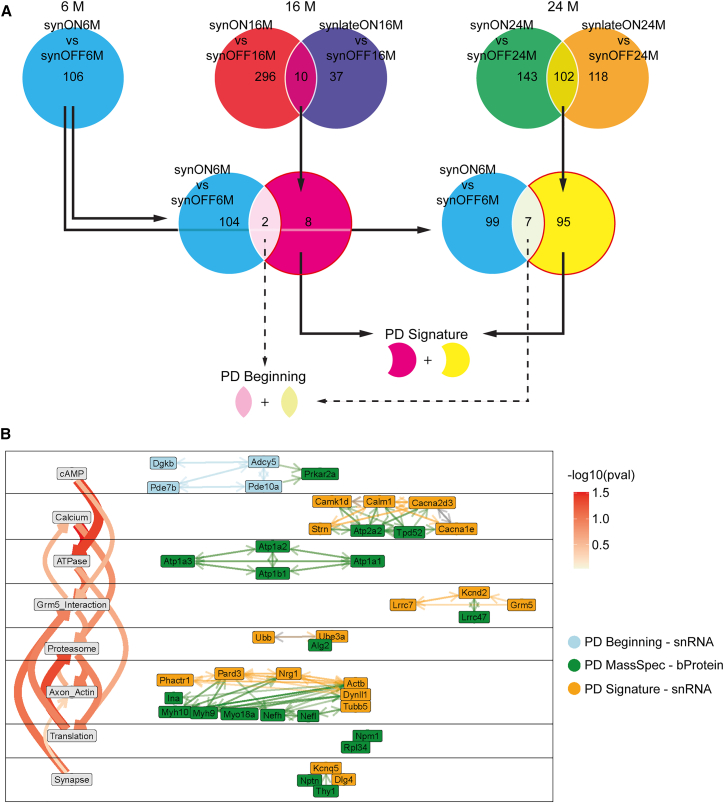
PD Signature points to early cAMP disruption and downstream actin/proteasome dysregulation (A) Defining the “PD Signature” and “PD Beginning” gene set. Based on the Rotarod data we decided to only consider intersection genes between the comparisons “synON vs. synOFF” and “synlateON vs. synOFF” at 16 M and 24 M for PD Signatures. From those intersections the genes that were not differentially expressed in “synON6M vs. synOFF6M” were assigned to “PD Signature,” while genes present in “synON6M vs. synOFF6M” and the intersections were defined as “PD Beginning.” (B) Functional network using the central regulators from snRNA-seq PD Signature, PD Beginning genes, and bulk Protein (bProtein) PD Signature from MassSpec data. The interaction between genes/proteins and the *p* values (pval) for topic-topic interactions were obtained based on omniPath and STRING databases. Network only showed topics with at least two genes/proteins and the top 2 topic-topic connections for each topic. The network suggests an upstream role of cAMP even before onset of motor deficits. This is followed by calcium pathway and Grm5. Grm5 acts as an intersection between the more upstream pathways and the downstream effectors like Proteasome or Axon/Actin. Especially Axon/Actin showed high number of strongly interconnected genes/proteins. Additionally, it should be noted that the “Synapse” topic serves as a fallback category for genes/proteins with broad synaptic functions. Most genes/proteins assigned to other topics are also located in the synapse but have more specific subcellular roles.

To delineate likely underlying molecular mechanisms of the motoric phenotype, DEGs were identified in GABA-Rgs9 cells by employing sample-wise pseudo-bulking for GABA-Rgs9 cells and performing Wald testing via the DESeq2 package.^18^ Given that motor deficits were present in synlateON and synON animals at both 16 and 24 M compared to controls, we focused on DEGs present in synON vs. synOFF and synlateON vs. synOFF comparisons that are also shared between the two time points. These genes were compared to the DEGs from the comparison of synON6M vs. synOFF6M, as there were no motoric deficits in synON6M mice. We derived two central gene signatures from this comparison: (1) the “PD Beginning” signature of genes that are differentially expressed as response to S1/S2 expression prior to symptom and remain differentially expressed during motor deficits (DEGs present at both 6 M and in the intersection of 16 and 24 M) and (2) the “PD Signature” that represents genes differentially expressed only upon severe motoric PD (DEGs present at the intersection of 16 and 24 M that are not present at 6 M, Figure 6A). The PD Signature comprised 102 genes and the PD Beginning comprised 8 genes (Table S3). Additionally, using bulk Mass Spectrometry (MassSpec), we obtained a protein PD Signature based on differential comparisons that are identical to the comparisons for obtaining the RNA PD Signature (Figure S3 and Table S4). A final PD network was constructed by combining the snRNA-seq PD Signature, the snRNA-seq PD Beginning signature, and the bulk MassSpec protein PD Signature into a single representative network showing the most central genes of the signatures (Figure 6B). The snRNA-seq signature networks were based on synON24M transcriptional data in the GABA-Rgs9 cell type.

The functional module “cAMP” included all dysregulated “PD Beginning” (blue) genes, including downregulation of cAMP homeostasis genes (*Adcy5*, *Pde7b*, and *Pde10a*) and reduced protein levels of the cAMP target Prkar2a/protein kinase A (PKA) in all S1/S2-expressing PD conditions. This suggests dyshomeostasis of cAMP levels and reduced PKA activity to be an early impairment in the course of the disease and to be directly associated to elevated levels of S1/S2 oligomers. The calcium pathway containing downregulation of calcium influx channel gene expression (*Cacna2d3* and *Cacna1e*) alongside an upregulation of calcium-binding signaling genes (*Calm1* and *Calm3*; Table S3) was identified as a response to S1/S2 oligomers in response to cAMP signaling, likely as an adaptive response to reduced calcium signaling. Both “cAMP” and “calcium” exerted strong direct and indirect effects on the glutamate metabotropic receptor 5 “Grm5_Interaction.” Genes upstream (*Lrrc7*) and downstream (*Kcnd2*) of the glutamate metabotropic receptor 5 (*Grm5*), as well as *Grm5* itself, were downregulated in the PD Signature. Interestingly, *Grm5* Interaction appears to function as a connector, as it receives signals not only from the upstream modules cAMP and calcium but also from the downstream modules Translation and Synapse, while at the same time effecting the downstream modules of Proteasome and Axon/Actin. The Proteasome and Axon/Actin modules exhibited the strongest ingoing connections, indicating more downstream roles during the course of the disease. Within the Axon/Actin module (Actin: *Actb*, *Nrg1*, *Pard3*, and *Phactr1*; Myosin/Dynein: MYH10, MYH9, MYO18A, and *Dynll1*; Tubulin: *Tubb5*, NEFH, and NEFL) the expression levels of actin genes were decreased, while myosin/dynein/tubulin protein levels were increased, implying a significant change in axonal transport mechanisms (Figure 6B; Tables S2 and S4). Notably, CDC42 activity can be a major regulator of cytoskeletal organization and synaptic activity.^19^

**Figure 7:**
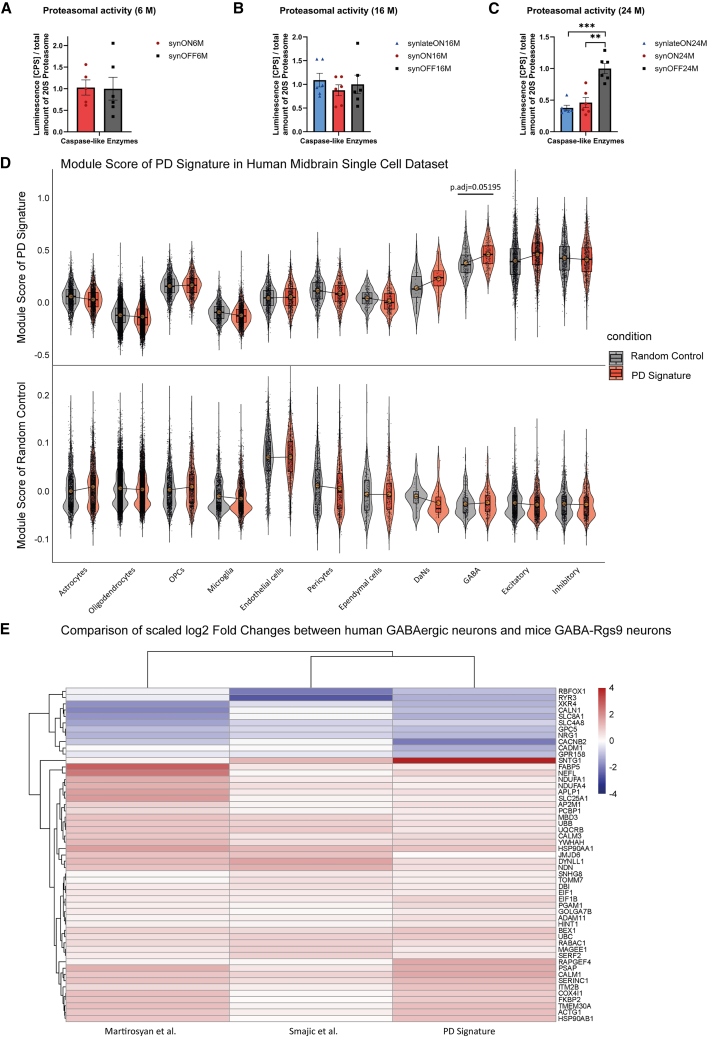
Age-related proteasomal impairment in mice and concordant PD Signature in human snRNA-seq data (A–C) Determination of proteasomal activity of caspase-like enzymes of full-brain lysates from S1/S2 animals by proteasome activity assay. Comparison of short-, long- and non-expressing animals aged (A) 6 M, (B) 16 M and (C) 24 M. Luminescence signal (counts per second [CPS]) is proportional to proteasomal activity and was normalized to total amount of 20S proteasome determined by western blot analysis (Student’s *t* test, ∗∗∗<0.001, ∗∗<0.01; data: mean ± SEM). (D) Top 20 PD Signature genes and 20 randomly sampled genes were used to calculate a Module Score on the human midbrain snRNA-seq dataset Smajic et al., 2022. *p* values were calculated by Wilcoxon test on sample-wise pseudo-bulked samples. Reflecting our finding in mice GABA-Rgs9 Neurons (GABAergic BGNs), the PD Signature is found to be most affecting the GABAergic neurons in the human midbrain. This effect is not observed in the random control (data: mean ± SD). (E) Heatmap of scaled log2 fold changes of genes from the PD Signature that were concordantly changed across mice GABA-Rgs9 synON24M vs. synOFF24M, Smajic et al., human PD vs. control in GABAergic neurons, and Martirosyan et al. PD vs. control in GABAergic neurons. We obtained a total of 53 concordant genes, including genes we deemed as central to our PD Signature, such as Ubb, Dynll1, or Calm1.

The data predict proteasomal dysfunction to be a central and downstream mechanism for motor dysfunction in the PD model (Figure 6B). To test these *in silico* findings experimentally, we determined protein degradation mechanisms in full-brain lysates. We investigated both the ubiquitin-proteasome system (UPS) and the autophagic-lysosomal pathway, which have also been previously shown to be dysregulated in PD patients.^20^^,^^21^ While the autophagy system (Figures S5A–S5C) and the chymotrypsin- and trypsin-enzymatic protease activity (Figures S5G and S5H) was not affected, reduced caspase-like enzymatic protease activity confirmed downregulation of the UPS in synlateON24M or synON24M animals compared to the control mice (synOFF24M) (Figures 7A–7C). This is in line with our *in silico* results, which obtained UPS-associated genes (*Ubb* and *Ube3a*) as central genes of our PD Signature network (Figure 6B).

Finally, we correlated our PD Signature to an snRNA-seq dataset of human PD midbrains.^9^ Comparing our top 20 central PD Signature genes with the human dataset, there was a substantial increase in the PD vs. control module scores in Dopaminergic Neurons (DaN), GABAergic Neurons, and Excitatory Neurons (Figure 7D), which was not observed in a random control experiment (Figure 7D). Additionally, we used another snRNA-seq dataset of human PD midbrains with 15 PD patient samples and 14 controls.^7^ We performed pseudo-bulked DGE analysis in GABAergic neurons for both datasets (Tables S6 and S7) and compared the log2 fold changes from our PD Signature from mice GABA-Rgs9 neurons to the log2 fold changes in the human GABAergic neurons (Figures 7E and S6). We identified 53 genes with concordant expression changes in both human and mouse GABAergic neurons (Figure 7E). These 53 genes included the central PD Signature genes such as *Ubb*, *Calm2*, or *Nrg1* (Figures 7E and S6).

Our findings are consistent with the idea that early PD may involve alterations in cAMP and calcium signaling, which could be linked to reduced ATP production and impaired Grm5 function. Such changes might contribute to proteasomal and axon/actin system disturbances observed in GABA-Rgs9 neurons in mice and in other neuronal populations in humans, which in turn may be associated with motor deficits in aging-related PD.

**Figure 8:**
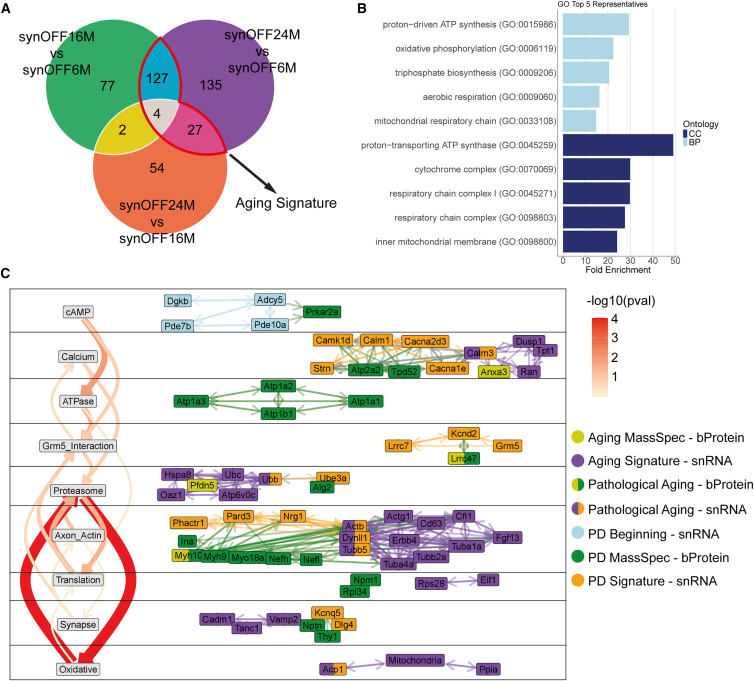
Aging impairs proteasomal activity through increased oxidative stress-related disruptions (A) Aging Signature contains all genes differentially expressed in “synOFF24M vs. synOFF6M” as well as in “synOFF16M vs. synOFF6M” or “synOFF24M vs. synOFF16M.” (B) The Aging Signature was heavily influenced by genes specific to mitochondrial function and obscured other topics. Thus, all those genes were summarized under the term “Mitochondria.” (C) Final functional network combining the PD network and the top 40 central regulators from the non-mitochondrial Aging Signature. Notably, aging not only affected many same functional topics found in PD but also introduced strong topic-topic interaction pathways, especially the interaction between Proteasome and Oxidative Damage. We also found six genes that play a central role in both PD and aging and thus were defined as “Pathological Aging-snRNA”: Calm3, Ubb, Actb, Dynll1, Tubb5, and Acp1, and two “Pathological Aging-bProteins”: Lrrc47 and Myh10.

Aging is the causative driver for the onset of PD in the α-syn model. We, therefore, further identified an Aging Signature (similar to the identification of the PD Signature) by comparing the DGE synOFF16M vs. synOFF6M (6 M → 16 M aging), the synOFF24M vs. synOFF16M (16 M → 24 M), and the synOFF24M vs. synOFF6M (6 M → 24 M). Aging Signature genes were identified as genes that were concordantly changed throughout all age stages, meaning only genes exhibiting the same direction of differential expression across all the three comparisons have been considered associated with aging and were included in the Aging Signature (Figure 8A). As previously conducted for the PD Signature, the Aging Signature derived from MassSpec data was also integrated into the Aging-related network (Figure S3; Table S5). The functional network for aging was constructed using the same methodology as for the PD Signature (Figures S2 and S5). We grouped genes related to mitochondrial function, due to their abundance, under the category “Mitochondria” encompassing all mitochondria-related genes (section inference of central regulators in STAR Methods; Figures 8B and S4; Table S5). Since the Aging Signature network contained nearly twice as many regulators compared to the PD Signature network, our analyses now comprised the top 40 non-mitochondrial central regulators.

The Aging Signature held functional modules that were very similar to the ones already identified in the PD Signature: Calcium, Proteasome, Axon/Actin, and Oxidative Damage. Interestingly, there was a direct overlap including eight genes/proteins (*Calm3*, *Ubb*, *Actb*, *Dynll1*, *Tubb5*, *Acp1*, MYH10, and LRRC47), which we designated as “Pathological Aging” signature. These genes/proteins likely serve as central regulators in both PD and aging (Figure 8C). Expression of the Pathological Aging signature genes were also consistently upregulated in the human snRNA-seq PD datasets (Figure S6). The data further imply that dyshomeostasis of cAMP levels and reduced PKA activity are early impairments specific to the presence of S1/S2 oligomers, since dyshomeostasis of cAMP levels and reduced PKA activity were not present in the Aging Signature.

Together, we suggest that α-syn oligomers evoke dyshomeostasis of cAMP-levels and reduced PKA activity as early response and are necessary to develop PD symptoms; however, the presence of these oligomers alone is not sufficient for disease development. For PD disease development, molecular mechanisms of aging (cytoskeleton rearrangement [axon-actin], oxidative damage, and proteasomal dysfunction) are necessary and sufficient for the development of the phenotypic disease.

## Discussion

The findings presented here provide important insights into the interplay between aging-related processes and PD progression in a model driven by α-syn oligomer formation. We demonstrate that the levels of aggregated α-syn do not show significant differences across the different age groups for the lifelong α-syn expression. Our data suggest that the onset of motor symptoms may not be directly correlated with α-syn oligomer load and that oligomer formation may precede motor decline in aged mice. These results indicate that the combination of aging and α-syn oligomers contributes to a PD-like motor phenotype, rather than either factor alone.

Support for the idea that aging modulates susceptibility to pathological α-syn propagation comes from the study by Vena den Berge et al.^22^ Here intragastrical injection of α-syn preformed fibrils into young rats did not result in central nervous system pathology, whereas old rats clearly showed α-syn pathology throughout the brain. This indicates that aging influences the brain’s susceptibility to α-syn-induced pathology rather than the aggregation process per se.

Further evidence also comes from our snRNA-seq data. SnRNA-seq identified that the PD Signature is indeed partially overlapping with an Aging Signature in GABA-Rgs9 that is most affected by the expression of the α-syn oligomers. Attenuation of Cdc42 activity, previously linked to restoration of aging-related cellular functions,^23^ alleviated PD-like symptoms in aged mice without reducing oligomer levels, suggesting that aging-associated processes are required for symptom onset.

Aging remains the single largest risk factor for most chronic diseases. In PD, aging is recognized as the strongest risk factor.^24^ Our α-syn-inducing PD model provides experimental support for the geroscience concept, in which age-related cellular and molecular changes contribute to disease progression. Specifically, α-syn oligomers evoked early dysregulation of cAMP/PKA signaling, but oligomer load alone was not sufficient to drive phenoconversion. For PD disease development, molecular mechanisms of aging like cytoskeleton rearrangements, oxidative damage, and proteasomal dysfunction are necessary.

Pharmacological attenuation of elevated CDC42 activity in aged animals ameliorated motor dysfunction without affecting α-syn oligomer levels, suggesting that targeting CDC42 might be beneficial in regard of aging aspects including actin cytoskeleton rearrangement and endocytosis among others. It cannot be determined which specific aging-related mechanism is directly targeted by CDC42 inhibition, or which restored mechanism is responsible for the observed motor improvements.

Our data suggest that α-syn oligomers reduce PKA signaling by disrupting cAMP homeostasis (e.g., via downregulation of *Adcy5* and *Pde10a*). Since PKA inhibits CDC42 activity, reduced PKA signaling could exacerbate CDC42 activation. Elevated CDC42 activity, already linked to aging-related cellular changes,^14^ may reach a pathological threshold when combined with α-syn-induced disruption of PKA signaling. We have already demonstrated that α-syn oligomers bind CDC42 effector proteins and may thereby influence CDC42 signaling.^25^ This may help explain why PD symptoms manifest primarily during aging.

Our snRNA-seq and network analyses identified key pathways dysregulated by aging, including cAMP and calcium signaling, proteasomal function, and cytoskeletal dynamics. The aging signature shared substantial overlap with PD-associated pathways, particularly in proteasomal and axon/actin modules, supporting the idea that “Pathological Aging” contributes to PD phenoconversion.

Notably, the cAMP pathway was deregulated before symptom onset and remained altered over time, suggesting it may represent an early vulnerability. However, additional age-related changes, such as calcium signaling deficits, appear to be required for PD-like phenotypes. Calm3, a member of our Pathological Aging gene set, encodes calmodulin, a calcium-binding protein and a key calcium sensor, which regulates synaptic activity.^26^ Calcium imbalance increases with aging and has been implicated in PD.^27^ We find Calm1 and Calm3 to be upregulated in human PD brain. PD patients’ brains show elevated intracellular calcium, especially in substantia nigra neurons, where L-type Cav1.3 calcium channels are highly expressed.^28^^,^^29^ The calcium channel blocker isradipine improved motor function and reduced neurodegeneration in animal models.^30^ Finally, excess calcium promotes α-syn aggregation by exposing its aggregation-prone NAC region.^31^ Our findings of enrichment of α-syn oligomers in aged PD animals suggest a feedback loop where excess calcium drives α-syn aggregation to further negatively influence PKA/CDC42 signaling.

It is well known that proteasomal dysfunction is linked not only to aging but also to PD, with atypical ubiquitination of proteins like α-syn promoting insoluble aggregates.^32^^,^^33^ Loss of proteostasis is also considered a hallmark of aging.^34^ Our PD Signature shows changes in *Ubb* and *Ube3a*, while we also found altered levels of *Ubb* in our Aging Signature, supporting a role for proteasomal dysfunction as part of Pathological Aging and PD. Reduced proteasome activity in aged animals further supports dysfunction as a late-stage effect. Among aging genes, Acp1 may connect oxidative damage, PD, and proteostasis decline, although mechanistic links (e.g., Acp1-Cdc42) remain to be clarified.

In summary, we report that α-syn oligomers reduce activity in the cAMP/PKA pathway in an early non-symptomatic phase, while molecular mechanisms of aging like calcium signaling dysregulation, cytoskeletal rearrangements, oxidative damage, and proteasomal dysfunction likely act together to drive symptom onset. These findings support the idea that aging creates a permissive environment for α-syn oligomers to contribute to PD progression, although further studies are needed to clarify the exact mechanisms.

### Limitations of the study

This study has limitations. First, the mouse model used in this study employs a generic neuronal promoter, which does not limit the expression of the α-syn oligomers to a specific cell population, e.g., dopaminergic neurons in the substantia nigra resulting in a transgenic α-syn overexpression mouse model. Second, the overexpression of human α-syn takes place in the murine α-syn background, which might affect aggregation kinetics, seeding potency, and the structural characteristics of the resulting fibrils.^35^ Third, the human α-syn is tagged with a luciferase reporter, which, therefore, might not fully recapitulate PD phenotype in all aspects of the human disease. Another important limitation concerns the specificity of the CDC42 inhibition-related processes. CDC42 inhibition is associated to a range of aging-associated processes including regulation of the actin cytoskeleton, cell polarity, morphology and migration, endocytosis and exocytosis, cell cycle, and proliferation in many different cell types. Therefore, direct translation of our findings may be limited.

## Resource availability

### Lead contact

Requests for further information and resources should be directed to and will be fulfilled by the lead contact, Karin M. Danzer (karin.danzer@dzne.de).

### Materials availability

This study did not generate new unique reagents.

### Data and code availability

• Data: All new raw snRNA sequencing data synthesized for this study can be accessed on ArrayExpress under the accession number E-MTAB-14889. Raw mass spectrometry data have been deposited to the ProteomeXchange Consortium via the PRIDE^36^ partner repository with the dataset identifier PXD069528. Human snRNA data from Smajic et al., 2022, can be accessed at GEO under GSE157783 and data from Martirosyan et al., 2024, can be accessed at GEO under GSE243639.
• Code: All original code has been deposited on Zenodo and is publicly available at https://doi.org/10.5281/zenodo.17597372 as of the date of publication.
• Other: Preprocessed data have been deposited on Zenodo and are publicly available as of the date of publication at https://doi.org/10.5281/zenodo.14893736.

## Acknowledgments

We thank R. Bück, S. Meier, A. Erk, and A. Jesionek for their excellent work and technical support.

This work was supported and funded by the 10.13039/501100001659Deutsche Forschungsgemeinschaft (10.13039/501100001659DFG) Emmy Noether Research Group DA 1657/2-1, GRK 1789 (CEMMA), and SFB 1506 (Aging at Interfaces).

## Author contributions

V.B., M.K., and K.M.D. designed the project. V.B. performed experiments and analyzed the data. J.K. supported snRNA-seq experiments. J.LB. performed snRNA-seq and MassSpec analysis. P.O. performed MassSpec experiments and analysis. J.K.K., V.G., B. Mayer, B. Möhrle, and H.G. provided intellectual input. V.B., J.LB., and K.M.D. wrote the manuscript. All authors reviewed and approved the manuscript.

## Declaration of interests

K.M.D. and H.G. are listed as inventors on a patent application related to this technology.

## STAR★Methods

### Key resources table

**Table:** 

REAGENT or RESOURCE	SOURCE	IDENTIFIER
**Antibodies**		

Anti-α-Synuclein (human)	Enzo, Farmingdale, NY, USA	RRID:AB_2270759
Anti-α-Synuclein Syn-1	BD Bioscience, Franklin Lakes, NJ, USA	RRID:AB_398108
Anti-β-Actin	Bio-techne, Minneapolis, MN, USA	MAB8929
Anti-20S proteasome subunits	Santa Cruz Biotechnology, Dallas, TX, USA	RRID:AB_785332
Anti-LC3	Novus Biologicals, Minneapolis, MN, USA	RRID:AB_10003146
Goat anti-rat IgG (H + L) Cross-Adsorbed Secondary Antibody, HRP	Thermo Fisher Scientific, Waltham, MA, USA	RRID:AB_2535648
Goat anti-mouse IgG (H + L) Cross-Adsorbed Secondary Antibody, HRP	Thermo Fisher Scientific, Waltham, MA, USA	RRID:AB_2536527
Anti-rabbit IgG (H + L), HRP Conjugate	Promega, Madison, WI, USA	RRID:AB_430833

**Chemicals, peptides, and recombinant proteins**		

4′,6-Diamidino-2-phenylindole (DAPI)	Sigma-Aldrich, St. Louis, MO, USA	D9542
(2-Hydroxypropyl)-beta-cyclodextrin	Sigma-Aldrich, St. Louis, MO, USA	H5784
Acetonitrile (ACN)	Sigma-Aldrich, St. Louis, MO, USA	741857
AdaAhx_3_L_3_VS	Sigma-Aldrich, St. Louis, MO, USA	114802
Ammonium peroxydisulfate (APS)	Carl Roth, Karlsruhe, Germany	9592.1
Bovine Serum Albumin (BSA)	Carl Roth, Karlsruhe, Germany	T844.2
Calcium Chloride (CaCl_2_)	Sigma-Aldrich, St. Louis, MO, USA	223506
CASIN	Xcess Biosciences, Chicago, IL, USA	M60040
Coelenterazine	PJK GmbH, Kleinblittersdorf, Germany	102171
Dimethyl Sulfoxide (DMSO)	Sigma-Aldrich, St. Louis, MO, USA	D2650
Dithiothreitol (DTT)	AppliChem, Darmstadt, Germany	A2948
Dulbecco’s Phosphate Buffered Saline (DPBS)	Thermo Fisher Scientific, Waltham, MA, USA	14190–094
Ethylenediaminetetraacetic Acid (EDTA)	Carl Roth, Karlsruhe, Germany	8043.1
Glycine	neoLab Migge GmbH, Heidelberg, Germany	LC-4522.2
Hydrochloric Acid (HCl)	Merck, Darmstadt, Germany	30721
Ketamine (10%)	WDT, Garbsen, Germany	–
Magnesium Acetate (Mg(Ac)_2_)	Sigma-Aldrich, St. Louis, MO, USA	M5661
Methanol	Supelco, Bellefonte, PA, USA	1.06009.2511
Nonidet P40 Substituent (NP-40)	AppliChem, Darmstadt, Germany	A1694
Opti-MEM	Thermo Fisher Scientific, Waltham, MA, USA	11058021
OptiPrep Density Gradient Medium	Sigma-Aldrich, St. Louis, MO, USA	92339-11-2
PageRuler Prestained Protein Ladder	Thermo Fisher Scientific, Waltham, MA, USA	26616
RiboLock RNase Inhibitor	Thermo Fisher Scientific, Waltham, MA, USA	EO0384
Rompun (2%)	Bayer AG, Leverkusen, Germany	–
Roti-Block	Carl Roth, Karlsruhe, Germany	A151.1
Rotiphorese 30	Carl Roth, Karlsruhe, Germany	3029.1
Skim milk powder	Merck Millipore, Darmstadt, Germany	70166-500G
Sodium Dodecyl Sulfate (SDS) Solution (20%)	Carl Roth, Karlsruhe, Germany	A3942.1000
SPRIselect	Beckman Coulter, Brea, CA, USA	B23318
Sucrose	Sigma-Aldrich, St. Louis, MO, USA	S0389
SuperSignal West Pico PLUS Chemiluminescent Substrate	Thermo Fisher Scientific, Waltham, MA, USA	34577
TEMED	Carl Roth, Karlsruhe, Germany	2367.3
Triethylammonium bicarbonate (TEAB)	Sigma-Aldrich, St. Louis, MO, USA	T7408
Trifluoroacetic acid (TFA)	Sigma-Aldrich, St. Louis, MO, USA	8.08260
Tris(2-carboxyethyl)phosphine hydrochloride (TCEP)	Sigma-Aldrich, St. Louis, MO, USA	C4706
Tris (Hydroxymethyl-)Amino-Methane (Tris)	Sigma-Aldrich, St. Louis, MO, USA	T1503
Trypsin/LysC enzyme	Promega, Fitchburg, WI, USA	V5071
Trizma base	Sigma-Aldrich, St. Louis, MO, USA	T1503-1 KG
Tween 20	Carl Roth, Karlsruhe, Germany	9127.1
β-Mercaptoethanol	Sigma-Aldrich, St. Louis, MO, USA	M3148-25 ML

**Critical commercial assays**		

Anti-Mouse Detection Module	Bio-techne, Minneapolis, MN, USA	DM-002
Chromium Next GEM Chip G Single Cell Kit	10× Genomics, Pleasanton, CA, USA	PN-1000127
Chromium Next GEM Single Cell 3ʹ GEM, Library & Gel Bead Kit v3.1	10× Genomics, Pleasanton, CA, USA	PN-1000121
Dual Index Kit TT Set A	10× Genomics, Pleasanton, CA, USA	1000215
Gel Filtration Calibration Kit	GE Healthcare, Chicago, IL, USA	28403841
High Sensitivity D5000 Screen Tape/Reagents	Agilent, Santa Clara, CA, USA	5067-5592/5067-5593
Pierce^TM^ BCA Protein Assay Kit	Thermo Fisher Scientific, Waltham, MA, USA	23227
Proteasome-Glo Assays	Promega, Madison, WI, USA	G8531

**Deposited data**		

Raw 10× 3′ Chromium snRNA Sequencing Data	This Paper	ArrayExpress: E-MTAB-14889
Processed snRNA data	This Paper	Zenodo: https://doi.org/10.5281/zenodo.14893736
Raw Mass Spectrometry data	This Paper	https://github.com/DanzerLab/snRNA_PDMouseModel_Age MS_results.xlsx and MS2_results.xlsx PRIDE: PXD069528
snRNA Data from Smajic et al., 2022	Smajić et al.^9^	Gene Expression Omnibus: GSE157783
snRNA Data from Martirosyan et al., 2024	Martirosyan et al.^37^	Gene Expression Omnibus: GSE243639

**Experimental models: Organisms/strains**		

B6-Tg(ind alpha-syn split Luci)D;Camk2-tTA GVO	Kiechle et al.^5^	N/A

**Software and algorithms**		

Adobe Illustrator	Adobe Inc., San José, CA, USA	N/A
Analysis Code	This Paper	Zenodo: https://doi.org/10.5281/zenodo.17597372
Compass	Bio-techne, Minneapolis, MN, USA	N/A
Fusion	VILBER Lourmat GmbH, Eberhardzell, Germany	N/A
GraphPad Prism Version 9	GraphPad Software, Inc., San Diego, CA, USA	N/A
Julia	Julia, USA	N/A
Jupyter Notebook	Project Jupyter, USA	N/A
MS Office 2016	Microsoft Corporation, Redmond, WA, USA	N/A
Python 3.10.xx	Python Software Foundation, Wilmington, Delaware, USA	N/A
R 4.4.0	R Foundation for Statistical Computing, Vienna, Austria	N/A
Rotarod 1.2.0 software	Med Associates, Fairfax, VT, USA	N/A
R Studio	Posit Software, PBC, Boston, MA, USA	N/A
TapeStation System Software 4.1.1	Agilent, Santa Clara, CA, USA	N/A
Zotero	Zotero, Virginia Beach, VA, USA	N/A

### Experimental model and study participant details

#### Ethical statement

Mouse experiments were performed in accordance with the German Animal Welfare Act (Tierschutzgesetz) and in line with the local guidelines of the Animal Research Center, Ulm University.

#### Generation and housing of transgenic animals

Generation and a detailed description of the phenotype characterization has been previously published.^5^ The animals were housed at the Animal Research Center, Ulm University under standardized conditions. As the S1/S2 model is a Tet-off system, the transgene expression was suppressed by adding 100 mg/mL doxycycline in the drinking water, which was additionally sweetened with 10 g/L glucose. All animals were housed in groups in open polycarbonate type II long cages under controlled room temperature, humidity, a 12 h dark/light cycle and food and water *ad libitum*. The cages were enriched with nesting material and polycarbonate houses. Each subgroup of the experimental cohorts included 12 animals (balanced mixed sex groups) which underwent behavioral testing once a week.

### Method details

#### Behavioral experiment – Accelerating rotarod

Accelerating Rotarod (five lane Rotarod, Med Associates) was used to determine motor balance and coordination of all S1/S2 mouse cohorts, comparing animals with different ages (starting at 1, 11 and 19 M of age) and length of S1/S2 expression. For each time point, every mouse had to undergo three consecutive trials with breaks of 5 min in between. For one trial, the rotation of the rod increased from 4 to 40 rpm over a period of 300 s and the latency to fall was recorded by a software (Rotarod 1.2.0 software, Med Associates). For the analysis, mean values were used.

#### Protein complementation assay

The S1/S2 mouse model includes a bioluminescent protein complementation assay (PCA) expressing human wildtype α-syn fused to either the N- or C-terminal part of a Gaussia luciferase. Those halves are called S1 and S2, and are expressed simultaneously. After the aggregation of the expressed α-syn, the halves form a functional active luciferase that can be detected by the administration of the substrate coelenterazine.^38^ The expression takes place under the neuron-specific CamKIIα promoter^39^ and is inducible employing a Tet-off system driven by Doxycycline.^5^ The drinking water of the breeding cages always included doxycycline to avoid any transgene expression during embryonic development.

#### CASIN treatment

CASIN (Xcess Biosciences M60040) was freshly prepared on a weekly basis prior to each round of injections. Initially, the substance was dissolved in dimethyl sulfoxide (DMSO) to achieve a 100 mM stock solution, which was subsequently diluted using a (2-Hydroxypropyl)-beta-cyclodextrin solution (Sigma H5784). Mice received intraperitoneal injections at a dose of 25 mg/kg once daily, around midday, for four consecutive days. This cycle was followed by a one-week break, after which the four-day injection regimen was repeated. Treatments commenced when the mice were either 12 or 20 M old. Control animals received injections containing only the vehicle in equivalent volumes. CASIN was supplied as a lyophilized powder, and a single batch was consistently used throughout the entire experimental period.

#### Perfusion

Transcardial perfusion with 1× PBS of the whole animal was performed to rapidly remove the blood from the animals. At the age of 6, 16 or 24 M, the mice were terminally anesthetized with ketamine (100 mg/kg) and xylazine (16 mg/kg). After testing reflexes, the skin of the animal was cut ventrally under the ribs cage. The ribs were cut on the left side and lifted to expose the heart. The needle attached to a syringe filled with 50 mL PBS was put into the left ventricle and the right atrium was cut immediately to allow the blood and fluid to flow out of the organism. Afterwards, the brain was removed from the skull and fresh frozen in liquid nitrogen. Those samples were used for all following experiments, including SEC, Western blotting, snRNA-seq and proteome analysis.

#### Protein extraction of mouse brain tissue

Hemispheres of 5–6 animals per group (age 6, 16 or 24 M) were used for protein extraction for the following experiments (balanced mixed sex groups). For full-brain lysate preparation, one hemisphere per brain was homogenized in PBS with a ratio of 1 g tissue per 10 mL PBS. After homogenization using a TissueLyser II (Qiagen) for 2 × 2 min at 25 Hz, the homogenates were centrifuged at 20,800 g for 30 min at 4 °C. Then protein concentration of the supernatant was determined by BCA assay (Thermo Fisher Scientific) and used for the further experiments (SEC, Western blot analysis, Simple Western experiments and Proteasome Activity assay).

#### BCA assay

The protein concentrations of the full-brain lysates were determined by colorimetric two-component Pierce BCA Protein Assay Kit (Thermo Fisher Scientific) like indicated in the manufacturer’s instructions. The lysates were diluted 1:5, 1:10 and 1:20, and PBS was used as blank. The concentrations of the used protein standards (bovine serum albumin) ranged from 0 to 2 mg/mL (0, 125, 250, 500, 750, 1000, 1500, 2000 μg/mL). After mixing the fluorescent dye with the standard samples, lysates and blank, everything was incubated at 37 °C for 30 min and the fluorescence intensity was measured in duplicates using a Microplate reader (SPECTROstar Nano) at wavelength 562 nm.

#### Size-exclusion chromatography

The full-brain lysates from 5 to 6 mice (6, 16 or 24 M of age) of the animals which underwent behavioral testing (balanced mixed sex groups) were used for SEC performed with the Superdex 200 Increase 10/300 GL column (Cytiva). The column was connected to an Äkta pure system (Cytiva) and got equilibrated with two column volumes (CV) of filtered (0.22 μm filters) and degased PBS before use. A 1 mL loop was used for automated sample injection and 600 μL of each lysate was loaded onto the column. A flow rate of 0.75 mL/min for PBS was used for elution of the proteins from the column. The maximum pressure was set to 5 MPa and the eluted proteins were monitored by UV absorbance at 280 and 215 nm. The eluate was collected in fractions of 500 μL in deep-well plates for later experiments. 200 μL were used for duplicate measurement of luciferase activity and 100 μL were used for dot blot analysis. Determination of the molecular mass of the eluted proteins was performed using the Gel Filtration Calibration Kit (Cytiva) with the following standard proteins: Conalbumin (75 kDa), Ovalbumin (44 kDa), Carbonic anhydrase (29 kDa), Ribonuclease A (13.7 kDa) and Aprotinin (6.5 kDa). The void volume (v_o_) was determined using Blue Dextran.

#### Human gaussia luciferase assay

Duplicates of 100 μL of each protein-containing fraction of SEC were used for measurement of luciferase activity. Aggregated α-syn with luciferase halves reconstitutes the whole Gaussia luciferase which oxidatively decarboxylates the substrate coelenterazine with emission of light as the result. Coelenterazine (P.J.K) stock was prepared previously to the experiments to a final concentration of 1 mg/mL in methanol and stored at −80°C. The same batch was used for all measurements. The working solution of coelenterazine was prepared in Opti-MEM (40 μM) and incubated at RT for 25 min without light. 100 μL of the cell permeable substrate was directly added to each sample before measurement by the automated dispenser module of the plate reader (Victor X3 microplate reader, PerkinElmer). Luminescence was then measured at 480 nm with a signal integration time of 1 s. Luminescence signal was afterward normalized to protein concentration. Lysates from animals without S1/S2 expression which did not undergo SEC but were also tested by luciferase activity assay to verify that there is no remaining luciferase expression.

#### Dot blot

Additionally, the SEC fractions were tested for total human α-syn signal by using a Dot blot apparatus (Minifold I, Whatman). The system was used as described by the manufacturer. 100 μL of each protein-containing SEC fraction was filtered through a nitrocellulose membrane by vacuum to transfer the proteins. After drying the membrane for 10 min at RT, it was blocked with 1× Roti-Block (Carl Roth) for 1 h at RT. It was then incubated overnight with primary antibody in 1× Roti-Block (anti- α-syn 15G7 antibody, 1:100) at 4 °C. The membrane was washed 3 × 10 min with TBS-T and then incubated with secondary antibody in 1× Roti-Block (anti-rat HRP-conjugated goat antibody, 1:5000) for 2 h at RT. Afterwards, additional 3 washing steps were performed for 10 min with TBS-T each. Images were taken with the Fusion SoloS Imager (Vilber) with HRP substrate (SuperSignal West Pico PLUS, Thermo Fisher Scientific). The quantification of α-syn signal of SEC fractions was performed using the Fusion software.

#### Simple western

The Jess system (Bio-techne) was used to conduct a capillary-based Western blot analysis (Simple Western). The same full-brain lysates like for SEC (5–6 animals per condition; 6, 16 or 24 M; balanced mixed sex groups) underwent dilution and preparation according to the manufacturer’s protocol. Briefly, the samples were mixed with Simple Western Sample Buffer to achieve a final concentration of 0.4 mg/mL, followed by denaturation at 95 °C for 5 min. The samples, along with primary (anti- α-syn Syn1 antibody, BD, 1:100) and secondary (anti-mouse HRP antibody) antibodies, chemiluminescent substrate and wash buffer were dispensed into the 384-well plate. The Jess system executes all assay steps automatically by placing Simple Western assay buffers, capillaries and the prepared assay plate. Protein separation occurs within the capillaries as they migrate the stacking and separation matrix, leading to the immobilization of separated proteins on the capillary wall. Afterward, target proteins are identified by primary antibody and immunodetection takes place by an HRP-conjugated secondary antibody and chemiluminescent substrate. The Compass software (Bio-techne) determines automatically molecular weight and AUC for immunodetected proteins. The expression of Syn-1 was normalized to beta-actin.

#### Western blot

For Western blotting, 37 μL of each sample was used, including 25 μg protein of the same full-brain lysates like for SEC (5–6 animals per condition; 6, 16 or 24 M; balanced mixed sex groups) and 14 μL β-mercaptoethanol, and got heated for 5 min at 95 °C. The samples were separated by sodium dodecyl sulfate-polyacrylamide gel electrophoresis (SDS-PAGE), using polyacrylamide gels with a 16% separating and a 5% stacking gel in a XCell SureLock Mini-Cell Electrophoresis system (marker: PageRuler Prestained protein ladder, Thermo Fisher Scientific). The gels were started with 80 V and were increased to 100 V when the samples reached the separating gel (running buffer: 25 mM Tris, 192 mM Glycin, 1 g/L SDS). Next, the proteins were transferred from the gel to a pre-activated PVDF membrane using a XCell SureLock XCell II Blot Module (Thermo Fisher Scientific) with 25 V for 2 h (blotting buffer: 25 mM Tris, 192 mM Glycin, 20% Methanol). The transfer was verified by PonceauS staining. The following step included blocking of the membrane with TBS-T buffer (10 mM Tris-HCl, 150 mM NaCl, 0.05% Tween 20) containing 5% milk powder for 1 h. Overnight incubation of blots with primary antibodies (anti-LC3, Novus biologicals) diluted at 1:1000 in blocking solution occurred at 4 °C. The membranes underwent three 10-min washes with 1× TBS-T to eliminate unbound primary antibodies. The next step involved incubation with secondary antibody (anti-rabbit IgG, diluted at 1:5,000 in blocking solution) for 2 h at RT, utilizing HRP-conjugated goat secondary antibodies (Promega). Following three additional 10-min washes with 1× TBS-T, images were captured using a Fusion SoloS Imager (Vilber) with HRP substrate (SuperSignal West Pico PLUS, Thermo Fisher Scientific). Densiometric analysis was performed using the Fusion software.

For Western blots with the antibody against the 20S proteasome α1/α2/α3/α5/α6/α7 subunits (Santa Cruz), only 12% polyacrylamide gels and 40 μg protein per sample was used. Here, a nitrocellulose membrane was used instead of PVDF and the blocking solution was 1× Roti-Block in water (Carl Roth).

#### Proteasome activity assay

To evaluate proteasomal activity in mouse full-brain lysates, the same full-brain lysates like for SEC (5–6 animals per condition; 6, 16 or 24 M; balanced mixed sex groups) were used and a modified version of a commercially available indirect enzyme-based luminescent assay (Promega, G8621), utilizing a substrate designed for caspase-like activity was employed. The setup was adapted to measure proteasomal activity in total protein extracts from brain tissue according to Strucksberg et al. in 2009.^40^ The full-brain lysates were diluted to a concentration of 0.2 mg/mL with PBS. Subsequently, 50 μL, equivalent to 10 μg of total protein, was mixed with 50 μL of the luminescent reagent containing Ultra-Glo Luciferase and the signal peptide coupled to luciferin. All samples were incubated for 1 h in a 96-well plate at RT and the luminescence was then measured with a signal integration time of 1 s (Victor X3 Microplate reader, PerkinElmer). The luminescent signal strength in each examined lysate is directly proportional to the overall peptidase activity, representing the summation of proteasomal activity and non-specific peptidase activities originating from other enzymes present in the protein extract. The approach involved measurement with and without the addition of 30 μM of the irreversible and specific proteasomal inhibitor adamantine-acetyl-(6-aminohexanoyl)3-(leucinyl)3-vinyl-(methyl)-sulfone (AdaAhx3L3VS, Calbiochem). Proteasomal activity was determined as value by subtracting the non-specific background activity from the total peptidase activity. For a conclusive determination of specific proteasomal activity correlated with the proteasome quantity, the calculated proteasomal activity value underwent normalization using the densiometry data obtained from the 20S proteasome α1/α2/α3/α5/α6/α7 subunits immunoblot analysis.

#### Nuclei isolation

Nuclei were isolated from same mice (6, 16 or 24 M) that had previously undergone behavioral testing and biochemical analyses. Each group consisted of 6 animals (balanced mixed sex groups). Around 100 mg of brain tissue was cut from one hemisphere including 0 mm bregma to −5 mm bregma using a mouse coronal brain matrix (Cell Point Scientific). Two of those brain pieces from two different mice were pooled for the extraction. All procedures were carried out on ice. 1400 μL of homogenization buffer (320 mM Sucrose, 5 mM CaCl_2_, 3 mM Mg(Ac)_2_, 10 mM Tris HCl pH 8, 0.1 mM EDTA pH 8, 0.1% NP-40, 1 mM β-mercaptoethanol, 0.4 U/μL RiboLock in H_2_O) was added to a douncer including the brain tissue and mechanical disruption with around 40 strokes was performed using pestle A followed by pestle B. The resulting homogenate was then passed through a 70 μm Flowmi cell strainer, followed by a 40 μm Flowmi filter. Next, 700 μL of the homogenized suspension was mixed with 450 μL of the working solution (50% Opti-Prep, 5 mM CaCl_2_, 3 mM Mg(Ac)_2_, 10 mM Tris HCl pH 8, 0.1 mM EDTA, 1 mM β-mercaptoethanol in H_2_O). A gradient was prepared using the following components: 300 μL of 40% Opti-Prep (40% Opti-Prep, 96 mM Sucrose, 5 mM CaCl_2_, 3 mM Mg(Ac)_2_, 10 mM Tris HCl pH 8, 0.1 mM EDTA, 0.03% NP-40, 0.12 U/μL RiboLock in H_2_O), 750 μL of 30% Opti-Prep (30% Opti-Prep, 134 mM Sucrose, 5 mM CaCl_2_, 3 mM Mg(Ac)_2_, 10 mM Tris HCl pH 8, 0.1 mM EDTA pH 8, 1 mM β-mercaptoethanol, 0.04% NP-40, 0.17 U/μL RiboLock in H_2_O) and 700 μL of the mixture of homogenized tissue in working solution. The gradient underwent centrifugation at 10,000 g for 5 min at 4 °C. Following centrifugation, approximately 200 μL of the nuclei were aspirated and transferred into a 1.5 mL low-DNA-binding tube. To this, 250 μL of 2% BSA and 0.12 U/μL RiboLock in PBS was added. Subsequent centrifugation at 2,000 g for 3 min at 4 °C was performed and the supernatant was discarded. The resulting pellet was resuspended in 250 μL of 2% BSA and 0.12 U/μL RiboLock, followed by a second round of centrifugation at 2,000 g for 3 min at 4 °C. After discarding the supernatant, the pellet was once again resuspended in 250 μL of 2% BSA and 0.12 U/μL RiboLock and filtered through a 40 μm Flowmi cell strainer into a low-DNA-binding tube. Further centrifugation at 2,000 g for 3 min at 4 °C was carried out. The pellet obtained was then resuspended in 50 μL of 1× nuclei buffer (1× nuclei buffer of 10× Genomics, with 1 mM DTT, 1 U/μL RiboLock in H_2_O). For counting nuclei and quality check for nuclear membrane integrity, nuclei were stained with DAPI. The final nuclei were then directly used for the Single-nuclei RNA sequencing protocol.

#### Single-nuclei RNA sequencing

The Chromium 3′ Single Cell Library Kit (10× Genomics) was used to generate the Gel Beads-In-Emulsion (GEMs), following the guidelines provided by the manufacturer. Shortly, 22,000 of the isolated nuclei from mouse brain tissue were introduced into barcoded Gel Beads using the Chromium Controller. Following GEM-RT incubation, cDNA samples underwent recovery, purification and amplification through a cDNA amplification reaction. Quality assessments on the amplified cDNA were conducted using a High Sensitivity DNA Kit (Agilent) on a TapeStation platform. Libraries were subsequently generated through the processes of fragmentation and adaptor ligation. Sample Index PCR was executed and the resulting purified libraries underwent assessment on TapeStation using a High Sensitivity DNA Kit to evaluate fragment quality. The single-nucleus libraries were then sent to Novogene (United Kingdom) for sequencing with the NovaSeq 6000.

#### Sequencing and alignment of single nucleus samples

Paired 150bp snRNA-seq was performed using the 10× Genomics Gene Expression (GEX) 3′protocol with a NovaSeq 6000 sequencer. For the alignment of reads, a custom reference was created by adding the sequences of the S1/S2 transgene and the CamkIIa promoter to the mm10 mouse reference genome. Count matrices were obtained using the *cellranger count 7.1*^41^ pipeline, including introns. Six samples were mapped using the bwUni2.0 High-Performance Computing infrastructure.

#### Quality control and integration of cells and counts

The unfiltered count matrices were loaded into R and corrected for ambient mRNA using *SoupX 1.6.0*^42^ with default settings, adjusting “tfidfMin” settings between 0.9 and 1.3 depending on the sample. Seurat objects were created for each sample and subsequently merged. Cells were filtered out based on the following criteria: number of unique molecular identifiers (nUMI) < 2500, number of genes (nGene) < 1500, mitochondrial gene percentage >3%, ribosomal gene percentage >1.5%, or log10(Genes/nUMI) < 0.85. Next, doublets were removed using *scDblFinder 1.16.0*^43^ with default values. In addition, doublets based on sex were removed using *cellXY 0.99.0* with default values.

Normalization was performed using the SCTransform function on 4000 variable features with glmGamPoi method implemented in *Seurat 5.0.1*,^44^ and top 50 embeddings were obtained via *scVI (scvi-tools 1.1.1)*^45^ integration for sex, age, batch, and number of pooled animals. Clustering was done using the Leiden algorithm and visualized with Uniform Manifold Approximation and Projection algorithm (UMAP). Clusters represented by few samples, less than 100 cells, or a single batch, and not of conditions of interest, were removed. Clusters driven by ribosomal or mitochondrial genes, as well as markers of hindbrain and olfactory cell types, were also discarded. The steps from normalization onward were repeated until no further clusters needed removal. Final integration was performed using *harmony 1.2.0*^46^ with an integration diversity penalty (theta) of 2, followed by final clustering based on the top 30 harmony components and UMAP visualization. Each subsequent clustering for annotation of sub-cell types was computed following the same procedure.

#### Annotation of cell types

Clusters were annotated in a hierarchical manner using literature, the Mouse Brain Atlas (mousebrain.org)^17^ and markers identified via the FindConservedMarkers function in Seurat. First, neurons and non-neuronal cells were distinguished using mainly canonical markers, such as but not limited to Rbfox3 (neurons), Mbp (oligodendrocytes), Acsbg1 (astrocytes), Pdgfra (oligodendrocyte precursor cells), Cx3cr1 (microglia), Colec12 (vascular cells), and Ttr (choroid plexus cells). Neurons were further classified into Vglut1, Vglut2, GABA, cholinergic, and dopaminergic neurons. Vglut1 and GABA neurons were further sub-annotated. A full list of markers and cell types can be found in the Table S1.

#### Differential gene expression analysis for snRNA data

DEGs were identified using the Wald test from the DESeq2 package.^18^ Cells were pseudo-bulked per sample for each annotated cell type, normalized, and all ribosomal and mitochondrial-DNA genes were excluded. All genes that were not expressed in at least three samples at an expression level of three were excluded in the analysis. To ensure comparability, cell number variability between batches and conditions were limited to a maximum of 1.5-fold difference via random subsampling. Covariates for the design were obtained via the sva package^47^ and *p*-values were recalculated using *fdrtool 1.2.18*.^48^ Multiple testing correction was applied using the Bonferroni-Holm method. A cutoff of 0.05 for adjusted *p*-values for significance was used. Potential remaining batch effects were controlled by differential analysis between the batches and any significant “batch-genes” were removed from comparisons with confounding designs.

#### Defining gene signatures

The Aging Signature was defined as follows: All DEGs from the “synOFF24M vs. synOFF6M” comparison that were also concordantly significant in the “synOFF16M vs. synOFF6M″ or “synOFF24M vs. synOFF16M″ comparisons. Genes that were only significant in “synOFF16M vs. synOFF6M″ and “synOFF24M vs. synOFF16M″ were not included into the Aging Signature as those genes do not change concordantly through the ages.

The PD Signature was defined as follows: We focused on differential genes present in both the “synON vs. synOFF” and “synlateON vs. synOFF” comparisons at 16 M. The same approach was applied for 24 M. These intersection genes between synON and synlateON were then compared to DEGs from the “synON6M vs. synOFF6M″ comparison. From this comparison, we created two signatures: Genes present at both 6 M and in the intersection were designated as “PD Beginning”. Additionally, the "PD Signature" was defined as all intersection genes not present at 6 M. The results were combined to obtain the final “PD Signature” and “PD Beginning” gene sets.

#### Inference of networks

Using PIDC, the most interconnected genes were chosen based on T. E. Chan et al.^49^ PIDC, based on Partial Information Decomposition (PID), infers statistical dependencies between triplets of genes from a single-cell gene expression matrix by assigning a confidence score to each interaction. For network inference, synON24M GABA-Rgs9 neuron counts were used for the PD Signature and synOFF24M GABA-Rgs9 neuron was used for the Aging Signature. Only connections with a top 10% confidence score were used to build an interaction network using curated public databases of known interactions. The OmniPath database^50^ was accessed using OmnipathR 3.11.16^51^ and combined with interactions having a confidence score above 0.600 from STRING version 12.0.0.^52^ The final network was constructed by retrieving the interactions from the databases between the PIDC genes, allowing for a maximum of one interconnecting gene.

#### Inference of central regulators

Each vertex/gene was tested for significant over-connectivity using hypergeometric testing, comparing the number of connections of the gene in the obtained graph to the number of connections of the gene in the database. Vertices were ranked according to their adjusted *p*-values, and the top 20 genes were designated as central regulators for the PD Signature.

For the Aging Signature, mitochondria genes were biased to be defined as central regulator due to the high interconnectivity between genes specific to mitochondrial function, which would mask possible other effects. To find other relevant aging genes besides mitochondria function we summarized all genes represented in the KEGG term “Oxidative Phosphorylation” or in any GO-Cellular Component term containing “mitochondrial,” “respiratory chain,” “respirasome,” “NADH,” or “oxidoreductase” as “Mitochondria”. From the remaining genes, the top 40 genes were identified as central regulators as the number of significantly central genes were close to double in the Aging Signature compared to the PD Signature.

#### Topic categorization and interaction between topics

Central regulators were categorized into functional topics using the databases of GO,^53^ KEGG,^54^ UniProt,^55^ and GeneCard.^56^ An interaction score between categories was calculated as follows: A distribution of connections was synthesized by permuting the topic assignment of genes 10,000 times and recording the number of interactions between the topics. Using this distribution, *p*-values were obtained and -log10 of the *p*-values served as the interaction scores for the observed number of connections between the topics. The top two interaction partners for each topic were considered for further analysis. For final visualization, only topics with at least two assigned genes/proteins were included.

#### Label-free proteomic data synthesis of mouse brain tissue

Whole-brain lysates from same mice that had previously undergone behavioral testing and biochemical analyses (4–5 animals per condition; 6, 16 or 24 M; balanced mixed sex groups) were used for proteomic analysis. Tissue lysates containing 100 μg total protein (16–32 μL) were filled to 40 μL with 100 mM triethylammonium bicarbonate (TEAB) and Tris(2-carboxyethyl)phosphine hydrochloride (TCEP, final concentration 5 mM) and 2-chloroacetamide (final concentration 10 mM) were added. Samples were incubated at 95 °C for 10 min. After cool down, 180 μL 100 mM TEAB and a trypsin/LysC (Promega) solution (protein-to-enzyme ratio 50:1) were added and samples incubated at 37 °C overnight. The digestion was stopped by addition of 50 μL 6% TFA (final 1%). Peptides were fractionated using STAGE Tips (AttractSPE Disks Bio SDB, Affinisep) into 3 fractions using 15% acetonitrile (ACN, fraction 1), 24% ACN (fraction 2) and 70% ACN in 20 mM NH4-Formiat (pH10). The fractions were vacuum dried and resuspended in 12 μL 0.5% TFA. Peptides were separated using an UltiMate 3000 RSLCnano system and a PepMap100 C18, 20 × 0.075 mm, 3 μm trap column (Thermo) and a PepMap100 C18, 50 × 0.050 mm, 2 μm analytical column (Thermo). Mobile phase of the loading pump (trap column) was 0.05% TFA/2% MeOH (flow rate: 5 μL/min), the mobile phase of the nano pump (analytical column) was A: 4% DMSO/0.1% formic acid and B: 4% DMSO/76% acetonitrile/0.1% formic acid (flow rate: 150 nL/min) and peptides were separated with a 3 h gradient. Peptides were infused into a QExactive mass spectrometer (Thermo) and measured with data-dependent acquisition (Top15). Proteins were identified using MaxQuant 2.0.3.0 and the Mus musculus reference proteome from UniProt (downloaded 3rd February 2022). An FDR of 1% was used for peptide and protein identification and protein quantification was performed with the MaxLFQ algorithm (Cox, Hein et al. 2014).

#### Mass spectrometry data analysis

MassSpec data included four replicates per condition from two batches. First, proteins detected in at least two samples in both batches were retained. Subsequently, the data was further filtered for proteins measured in a minimum of 75% of replicates for at least one condition. All methods thereafter, including Normalization, missing value imputation, and differential expression analysis, were performed using the DEP package and its wrapper functions. Furthermore, normalization between batches was facilitated by certain samples being purposefully remeasured in both batches.

For imputation of the remaining missing values, protein expression values were classified as either missing at random (MAR) or missing not at random (MNAR). A value was defined as MNAR if the proteins was detected in at least 75% of replicates of one condition while the other condition had 75% missing values. The imputation algorithms were chosen in the DEP function “impute” with “knn” implementation set for MAR values and “MinProb” set for MNAR values. A more detailed explanation of the algorithms can be found in the official DEP package documentation. Three replicates (synlateON16-4, synON16-3, and synOFF16-4) made a subcluster independent of their conditions. Thus, differential analysis was performed without those three samples. Differential expression analysis was conducted with the wrapper function for limma in DEP, using sex and batch information as covariates, and significance set at a Bonferroni-Holm adjusted *p*-value below 0.1.

#### Comparing results with human data

To validate our findings against published human data, we utilized the dataset from Smajic et al. (2022).^9^ It included five clinically diagnosed idiopathic PD and six age-sex matched control frozen postmortem midbrain tissues. The “CADPS2+” cell type was excluded from the analysis. We computed a Module Score using the top 20 PD Signature genes + Calm3, employing the AddModuleScore function of Seurat. As a random control, we subsampled an equivalent number of genes as for the PD Signature from the human dataset and calculated a corresponding Module Score. *p* values between conditions were calculated by unpaired Wilcoxon testing on sample-wise pseudo-bulked samples. Additionally, we compared the log2FoldChanges between the synON24M versus synOFF24M conditions in mouse GABA-Rgs9 neurons and the PD versus Control conditions in human GABAergic neurons.

Additionally, we used another snRNA-seq dataset of frozen postmortem human PD midbrains with 15 clinically diagnosed PD patient samples and 14 controls.^7^ Cells were filtered and processed according to the original publication. A cluster was annotated as GABAergic Neurons when all four relevant markers showed enriched expression (*GAD1*, *GAD2*, *SNAP25*, *SYT1*). We performed pseudo-bulked DGE analysis using covariates from sva and sex as known covariate in GABAergic neurons for both datasets and compared the log2 fold changes from our PD Signature from mice GABA-Rgs9 neurons to the log2 fold changes in the human GABAergic neurons. For this, all log2 Fold Changes were scaled to −4 to 4 to make datasets comparable.

### Quantification and statistical analysis

Statistical analysis was performed and graphs were created with Prism (GraphPad Prism 10). For behavioral analysis, repeated-measures two-way ANOVA was performed. All tests for significance were two tailed with a = 0.05. Values are presented as mean ± SEM (standard error of the mean). *p*-values <0.05 were considered as statistically significant with significance levels ∗*p* < 0.05, ∗∗*p* < 0.01 and ∗∗∗*p* < 0.001.
