# Supplementary material for: Targeting Cdc42 improves motor phenotype in Parkinson’s disease mice and reveals age-dependent susceptibility to α-synuclein
Source: iScience. 2025 Nov 25;28(12):114217. doi: 10.1016/j.isci.2025.114217 (PMC12744266; doi:10.1016/j.isci.2025.114217)
Supplement: Document S1. Figures S1–S6 and Tables S1–S5 [file mmc1.pdf]

iScience, Volume *28*

Supplemental information

Targeting Cdc42 improves motor phenotype in Parkinson's disease mice and reveals age-dependent susceptibility to α-synuclein

Verena Bopp, Jaehyun LeeBae, Patrick Oeckl, Julia K. Kühlwein, Veselin Grozdanov, Martin Kiechle, Benjamin Mayer, Bettina Möhrle, Hartmut Geiger, and Karin M. Danzer

SUPPLEMENTAL INFORMATION

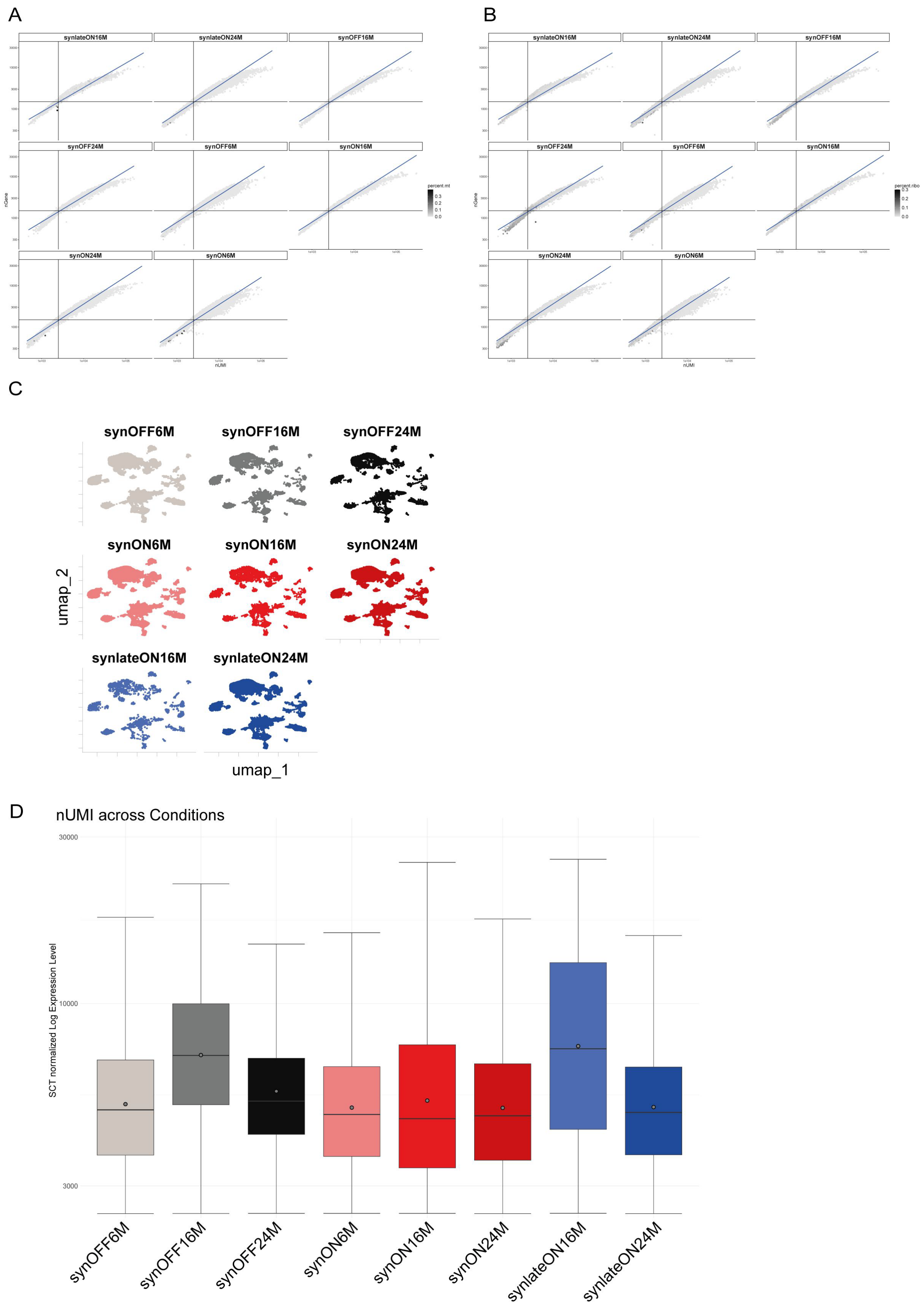

Suppl. Fig. 1: Quality Control and Integration of snRNA-seq data

(A) Scatterplot showcasing the number of Unique Molecular Identifiers (x-axis, nUMIs) and the number of genes (y-axis, nGene) per barcode across different conditions. Filtering thresholds are marked at 2500 for nUMI and 1500 for nGene on the respective axes. The color of the points represents the percentage of mitochondrial genes detected per barcode, with darker points indicating higher values. (B) Similar to (A), but here the color of the points represents the percentage of ribosomal genes. The scatterplots indicate comparable sequencing across conditions and barcodes with high mitochondrial or ribosomal gene percentages are found mostly in the filtered barcodes. (C) Results after data integration, showing that all clusters and cell types are represented across all conditions, even though cell numbers do vary between conditions: synOFF6M 23,324 - synON6M 24,015 - synOFF16M 5,346 - synlateON16M 3,407 - synON16M 5,453 - synOFF24M 5,921 - synlateON24M 15,016 - synON24M 23,207. (D) The nUMI was comparable between all conditions.

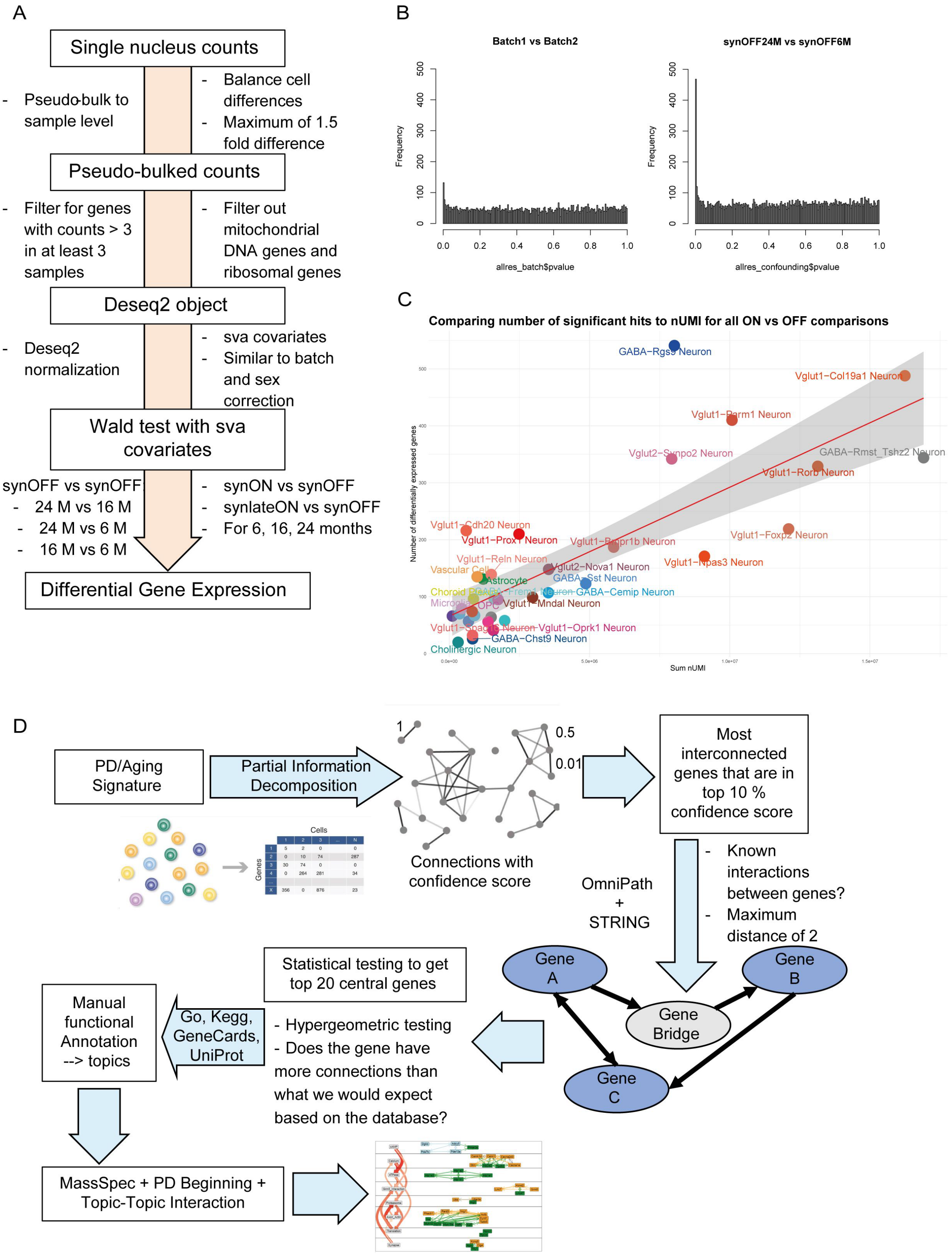

Suppl. Fig. 2: Pipeline and additional information for differential gene expression analysis

(A) Pipeline for differential expression gene (DEG) analysis. (B) To verify that batch effects do not significantly impact the results, p-value histograms were generated for comparisons between Batch1 vs. Batch2 and synOFF24M vs. synOFF6M, where the batch was a confounding factor. The histograms show minimal batch effects. (C) Scatterplot depicting the

total number of DEGs in each cell type for synON vs synOFF comparisons, plotted against the number of UMIs (nUMI) per cell type with a red regression line and the 95% confidence interval (grey zone). Cell types with low nUMI scatter further from the expected regression line. This is likely due to insufficient nUMI and thus differential hits are more likely technical differences. Notably, the GABA-Rgs9 Neurons, our cell type of interest, shows a significantly higher number of differential hits compared to other cell types. (D) Network inference pipeline is illustrated. Using the single nucleus count matrix of one condition for the signature genes in PIDC, an adjacency matrix with confidence scores for all interactions is inferred. The top interconnected genes are then integrated with curated interaction databases, OmniPath and STRING, to construct a biologically relevant network. Central regulators are identified via hypergeometric testing and manually annotated into functional topics. This network is then combined with bulk-protein data and other signatures. Topic-topic interaction scores are calculated to generate the final network graph.

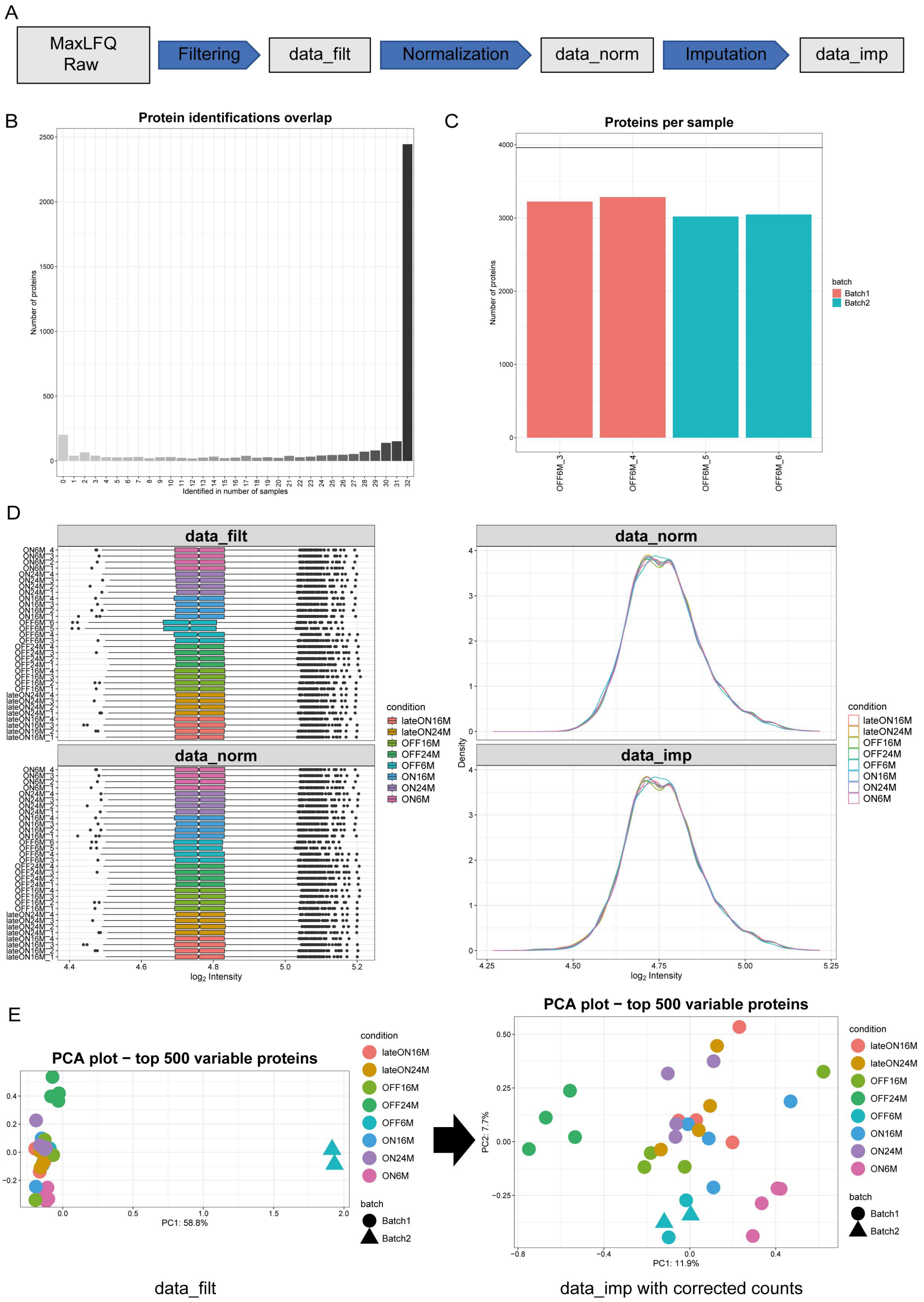
A
MaxLFQ Raw
Filtering
data_filt
Normalization
data_norm
Imputation
data_imp
B
Protein identifications overlap
Number of proteins
Identified in number of samples
C
Proteins per sample
Number of proteins
batch
Batch1
Batch2
OFF6M_3
OFF6M_4
OFF6M_5
OFF6M_6
D
data_filt
data_norm
data_norm
data_imp
condition
lateON16M
lateON24M
OFF16M
OFF24M
OFF6M
ON16M
ON24M
ON6M
Density
log2 Intensity
E
PCA plot − top 500 variable proteins
PC1: 58.8%
PC2: 10.7%
PC1: 11.9%
PC2: 7.7%
batch
Batch1
Batch2
data_filt
data_imp with corrected counts

Suppl. Fig. 3: Preprocessing of Mass Spectrometry data

(A) Analysis Pipeline for bulk Protein data from MassSpec. It consists out of filtering the raw MaxLFQ counts, followed by normalization and imputation of missing values. (B) Most proteins were detected across all 32 replicates. (C) However, the two replicates from Batch2 showed a lower detection rate compared to those from Batch1. To address this, proteins only detected in both batches in at least two samples were kept. Subsequent filtering was performed to retain proteins measured in at least 75% of replicates in a minimum of one condition. (D) After filtering and normalization, the remaining missing values were imputed, which did not alter the dataset's structure. (E) By incorporating the batch effect as a covariate in the differential analysis design, this method effectively removed the batch effect from the dataset.

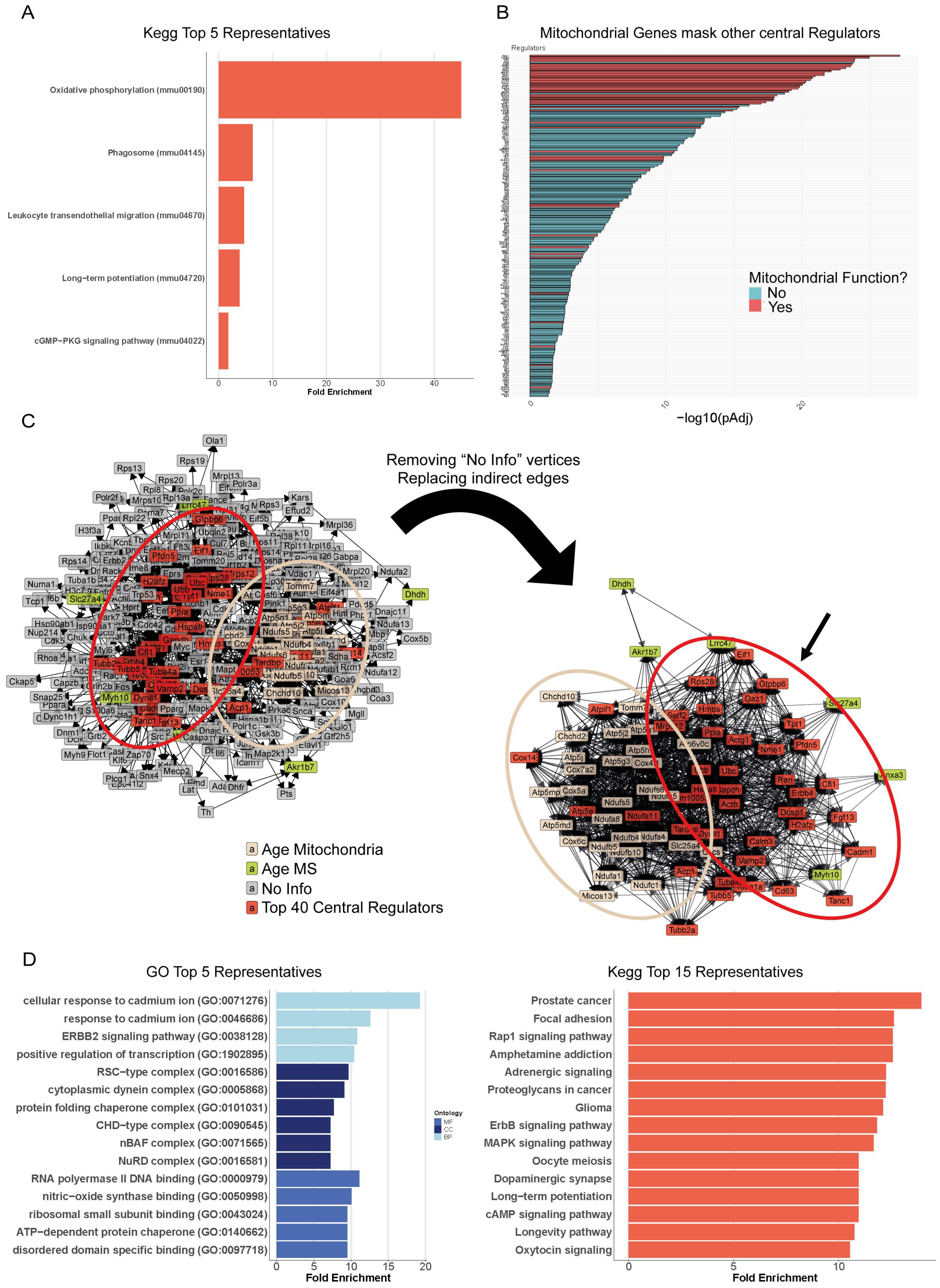

Suppl. Fig. 4: Characterization of aging signature

(A) Top 5 KEGG-Terms representing the genes in the Aging Signature. Oxidative Phosphorylation, which represents the electron transport system in mitochondria, showcases highly significant Fold Enrichment compared to other terms. (B-C) This mitochondrial bias is

also evident when examining the top central regulators from the Aging Signature network. Most of the top 40 regulators are genes specific to mitochondrial function. (C) By summarizing and excluding mitochondrial-specific genes, we identify the relevant top 40 central regulators. As done for the PD Signature network, "No Info" vertices are removed, and connections to regulators are replaced with direct connections between the regulators. This network was further annotated, and inter-topic edges was removed for clearer visualization. (D) Gene Ontology (GO) and KEGG terms for the genes excluding mitochondrial-specific ones predominantly include metabolic and aging-related terms.

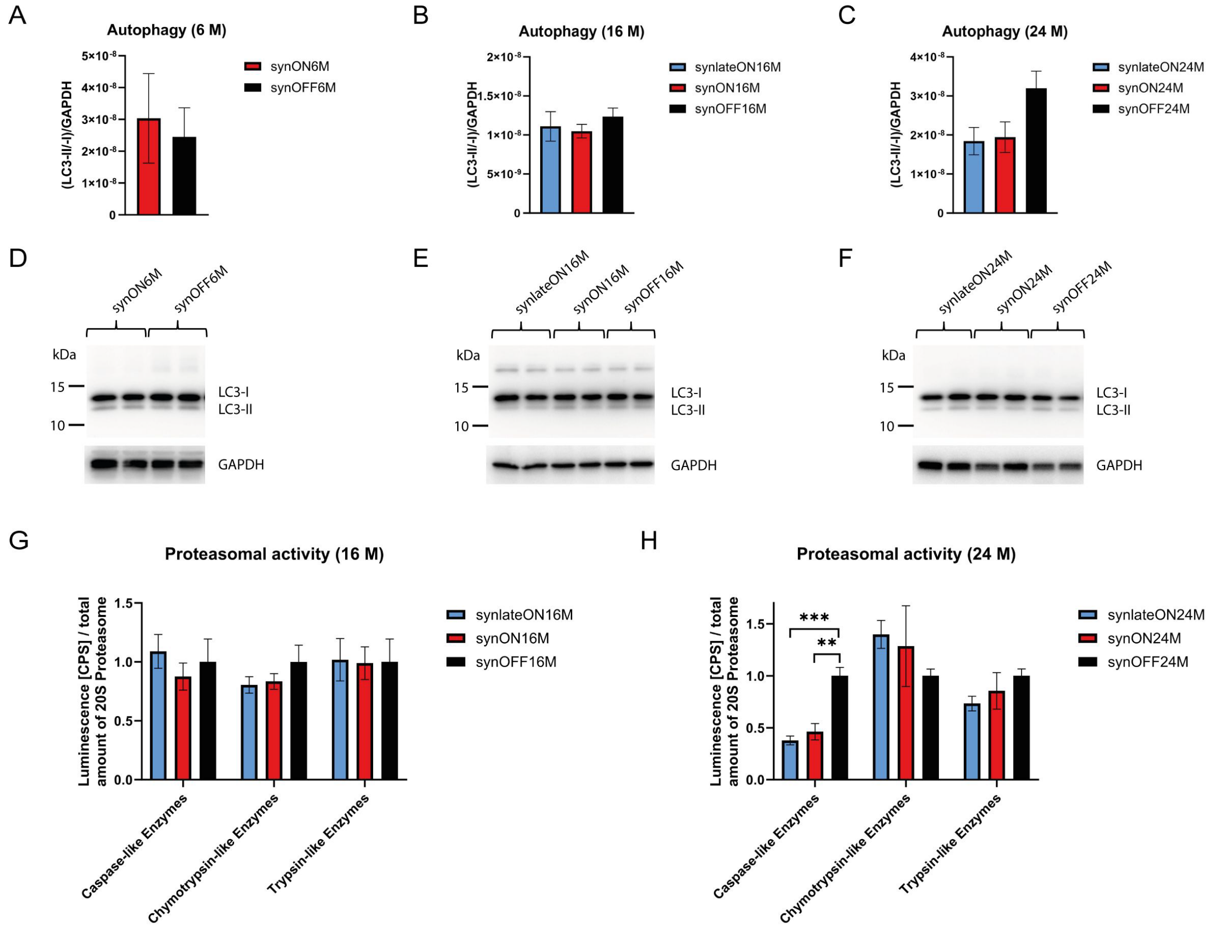

Suppl. Fig. 5: Measurement of autophagy and proteasomal activity in PD mice

(A-F) Determination of autophagy of full-brain lysates from S1/S2 animals by analyzing of autophagic marker LC3. Comparison of short-, long- and non-expressing animals with (A) 6 M, (B) 16 M and (C) 24 M of age. (D-E) Representative western blot pictures with autophagic marker LC3 and GAPDH as loading control. (G-H) Determination of proteasomal activity of Caspase-, Chymotrypsin- and Trypsin-like enzymes of full-brain lysates from all cohorts by proteasome activity assay. Comparison of the animals with (G) 16 M and (H) 24 M of age. Luminescence signal (counts per second, CPS) is proportional to proteasomal activity and was

*normalized to total amount of 20S proteasome determined by Western blot analysis (student's t-test, ***<0.001, **<0.01; data: mean ± SEM).*

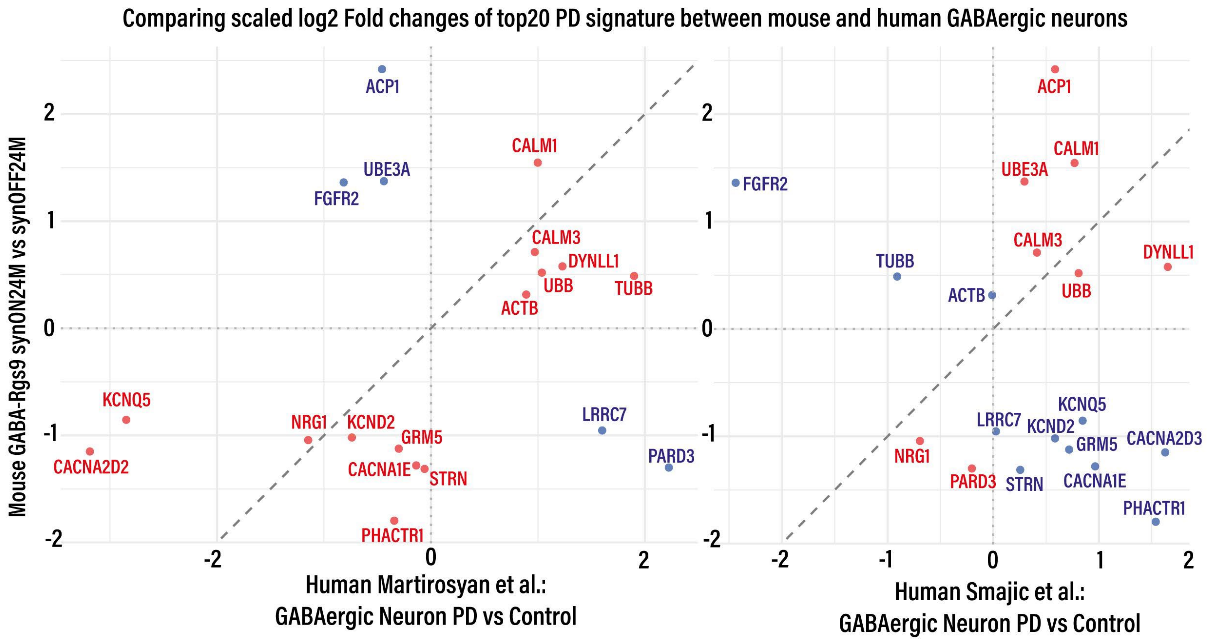

Suppl. Fig. 6: Comparison between mice and human data show concordance

(A) Scatter plot showing the Log2 fold changes for the PD Signature genes. Y-axis represents synON24M vs synOFF24M comparison in the GABA-Rgs9 cell type in our dataset while the x-axis represents PD vs Control comparison in GABAergic Neurons in the human snRNA dataset of human midbrain samples for Smajic et al. (Smajic et al., 2022) and Martirosyan et al. [7]. We observe a concordant change between our and the human dataset for all six Pathological Aging genes (Acp1, Actb, Calm3, Ubb, Dynll1, Tubb5). Ubb, Dynll1, and Calm3 were concordant in both human datasets, while Acp1, Actb and Tubb5 were only upregulated in one of the human datasets.

Suppl. Table 1: Used Annotation Markers for all Levels
Each header shows the cell type followed by rows of used annotation markers.

**Neuron**	**Astrocyte**	**Choroid Plexus**	**Microglia**	**Oligodendrocyte**	**OPC**	**Vascular Cell**
Rbfox3	Acsbg1	Ttr	Cx3cr1	Mbp	Pdgfra	Colec12
Slit3	Ndrg2	Slc4a5	Ikzf1	Mag	Cspg4	Prdm6
Cdh12	S100b	Rbm47	Hk2	Mog	Vcan	Slc6a13
Kcnj6	Ntsr2	Otx2	Slco2b1	Mobp	Cacng4	Eya2
Slc35f4	Atp13a4	Tmem72	Inpp5d	Prr5l	Itga9	Col1a2
Cntnap5a	Nhsl1	Kl	Dock8	St18	Calcrl	Cped1
Srrm4	Gjb6	Col9a3	Ptprc	Enpp6	Afap1l2	Bnc2
Zmat4	Etnppl	Trpv4	Runx1	Galnt6		Tbx15
Dlgap3		F5	Fyb			Aebp1
						Slc13a3
**Cortical Neuron**	**Hippocampus Neuron**	**Cortex/Hippocampus Interneuron**	**Basal Ganglia Neuron**	**Thalamus Neuron**		
Satb2	Shisa6	Kcnmb2	Rgs9	Shox2		
Sv2b	Nrp1	Maf	Actn2	Mctp2		
Stx1a	Iqgap2	Kcnip1	Gng7	Prkcd		
Khdrbs3	Wipf3	Alk	Sh3rf2	Synpo2		
Pdzrn3	Pkp2	Sox6	Grp88	Mrvi		
	Cdh9	Afap1	Drd2	Vangl1		
	Raver2	Col19a1	Drd1	Atp10a		
	Rnf182	Adra1a	Rxrg	Adamts19		
**GABAergic**	**Vglut1**	**Vglut2**	**Cholinergic**	**Dopaminergic**		
Gad2	Satb2	Synpo2	Chat	Slc6a3		
Gad1	Slc17a7	Slc17a6	Slc5a7	Th		
Dlx6os1	Ankrd33b			Lmx1b		
**Vglut2-Synpo2**	**Vglut2-Nova1**	**GABA-Rgs9**	**GABA-Otx2**	**GABA-Rmst/Tshz2**	**GABA-Cemip**	**GABA-Sst**
Synpo2	Erbb4	Rgs9	Otx2	Rmst	Cemip	Sst
Plekhg1	Nova1	Actn2		Tshz2	Nek7	
		Gng7				
		Sh3rf2				
		Grp88				
		Drd2				
		Drd1				
		Rxrg				
**GABA-Vip**	**GABA-Lamp5**	**GABA-Frem1**	**GABA-Chst9**	**GABA-Six3**	**GABA-Ano1/2**	**GABA-Etv1**
Vip	Lamp5	Frem1	Chst9	Six3	Ano1	Etv1

	Cripld1				Ano2	
**Vglut1-Foxp2**	**Vglut1-Cdh20**	**Vglut1-Parm1**	**Vglut1-Npas3**	**Vglut1-Mndal**	**Vglut1-Prox1**	**Vglut1-Reln**
Foxp2	Cdh20	Parm1	Npas3	Mndal	Prox1	Reln
Gng12	Tshz2	Shoc1	Ptpn14	Rerg	Prdm5	
**Vglut1-Rorb**	**Vglut1-Oprk1**	**Vglut1-Bmpr1b**	**Vglut1-Spag16**	**Vglut1-Shc4**	**Vglut1-Col19a1**	
Rorb	Oprk1	Bmpr1b	Spag16	Shc4	Col19a1	
	Col24a1					

Suppl. Table 2: DEGs from synON24M vs synOFF24M in GABA-Rgs9 neurons

baseMean: Mean expression; log2FoldChange: log2 transformed Fold Changes from DESeq2; lfcSE: shrinked log2FoldChange; stat: Wald statistic; pvalue: p value calculated by DESeq2; padj: Benjamini-Hochberg corrected pvalues

baseMean	log2FoldChange	lfcSE	stat	pvalue	padj	gene
1316,599352	0,76174103	0,135605	5,486129	2,22E-16	2,75E-13	Sntg1
24,2885356	0,241705225	0,093699	3,789783	2,22E-16	2,75E-13	Gm15478
18,33028577	0,210761544	0,086454	3,637374	2,22E-16	2,75E-13	Pebp1
22,11920783	0,227286176	0,091084	3,803705	2,22E-16	2,75E-13	Actg1
74,32080765	0,460629657	0,138875	3,581286	2,22E-16	2,75E-13	Acp1
109,5385411	0,501187596	0,144777	3,756415	2,22E-16	2,75E-13	Gm20642
17,43476545	0,193455484	0,082852	4,246891	2,22E-16	2,75E-13	Cox8a
32,50414054	0,354168521	0,105477	5,037402	2,22E-16	2,75E-13	Fth1
389,9216679	2,756753347	0,157289	12,2827	2,22E-16	2,75E-13	S1S2
3329,631162	-0,400395421	0,117494	-3,40002	6,66E-16	7,43E-13	Pde10a
11,5650379	0,128879105	0,069393	3,321945	3,33E-15	3,38E-12	Ndufa13
30,90724319	0,055357402	0,047165	3,257672	1,13E-14	1,05E-11	Tmsb4x
9562,950316	-0,342047471	0,108847	-3,17729	4,97E-14	4,27E-11	Phactr1
1880,736608	-0,383989207	0,126684	-3,16562	6,15E-14	4,57E-11	Cacnb2
11,82062065	0,139096979	0,072495	3,167063	6E-14	4,57E-11	Atp6v0e2
34,25864274	0,098916901	0,06316	2,946178	2,85E-12	1,99E-09	Ubb
7,258271556	0,08189267	0,056383	2,892392	7,02E-12	4,61E-09	Hint1
10,48619979	0,123684554	0,0689	2,80456	2,95E-11	1,83E-08	Cst3
7,743615553	0,087024304	0,058002	2,673942	2,31E-10	1,35E-07	Uqcrb
6,277656015	0,071110728	0,052774	2,66947	2,47E-10	1,38E-07	Dbi
225,923606	-0,467034473	0,158136	-2,62123	5,15E-10	2,74E-07	Gm26917
13,39871446	0,135551092	0,074418	2,56896	1,13E-09	5,71E-07	Chchd2
79,47918642	0,37019332	0,141472	2,553898	1,41E-09	6,03E-07	Slc24a5
5,851248724	0,066413415	0,051098	2,557878	1,33E-09	6,03E-07	Atp5k
5,567446445	0,063327015	0,049959	2,55476	1,39E-09	6,03E-07	Gm12027
44,84403116	0,276525198	0,113988	2,55641	1,36E-09	6,03E-07	Ly6h
5,768988163	0,065522512	0,050775	2,545701	1,59E-09	6,32E-07	Fabp5
11,51223951	0,114876072	0,068946	2,547792	1,54E-09	6,32E-07	Fam173a
2806,605862	-0,301313058	0,120337	-2,53309	1,91E-09	7,34E-07	Cntnap2
5,879148651	0,066673348	0,051192	2,529336	2,02E-09	7,5E-07	Gm11290

5,489461522	0,062457687	0,049636	2,488422	3,65E-09	1,31E-06	Edf1
5,109164023	0,058286809	0,048031	2,428199	8,58E-09	2,99E-06	Snhg8
688,5847049	-0,359491966	0,146143	-2,41166	1,08E-08	3,65E-06	Zbtb20
18,94644618	0,171738186	0,088401	2,404927	1,19E-08	3,9E-06	Tubb2a
1147,248844	0,319396857	0,134736	2,383629	1,59E-08	5,08E-06	Ctnna3
2614,047424	-0,280434021	0,120783	-2,37321	1,84E-08	5,7E-06	Dgkb
5,750208905	0,065222255	0,050664	2,346604	2,65E-08	7,98E-06	Ier5
1065,221162	-0,312374436	0,136201	-2,33712	3,01E-08	8,84E-06	Spock3
6,624351033	0,079747592	0,056696	2,317691	3,91E-08	1,12E-05	Mfge8
2125,902058	-0,287633058	0,125263	-2,31335	4,15E-08	1,16E-05	Pcdh9
42,1752584	0,093078835	0,063102	2,262986	8,09E-08	2,2E-05	Atp6v0c
1551,318419	-0,285065645	0,129547	-2,22992	1,25E-07	3,31E-05	Pde7b
7,911554789	0,094214877	0,061109	2,223343	1,36E-07	3,52E-05	Rnasek
2590,088878	-0,264165709	0,121278	-2,20771	1,66E-07	4,21E-05	Anks1b
1726,807365	-0,269910424	0,127358	-2,17388	2,55E-07	6,33E-05	Rora
85,68012137	0,310317278	0,143731	2,165233	2,85E-07	6,9E-05	Rapgef4os1
69,01740919	0,292157323	0,137846	2,141302	3,84E-07	9,12E-05	Scd2
6,207064411	0,074831326	0,055116	2,1241	4,76E-07	0,000111	Cox14
6,463536156	0,077761628	0,056078	2,112908	5,46E-07	0,000124	1810037I17Rik
19,21234059	0,172407976	0,091914	2,103895	6,1E-07	0,000136	Ubc
14,27836484	0,140683809	0,081741	2,100958	6,33E-07	0,000138	Eif1b
26,0326195	0,2091666	0,10286	2,096038	6,72E-07	0,000144	Selenok
82,00179457	0,265493653	0,142839	2,083759	7,81E-07	0,000164	2900097C17Rik
614,5409374	-0,286783428	0,148017	-2,05481	1,11E-06	0,000229	Prkg1
185,3936669	-0,307809301	0,157217	-2,04991	1,17E-06	0,000238	Efna5
4,000178129	0,046063266	0,042917	2,043873	1,26E-06	0,000251	Timm10b
25,19707904	0,182742404	0,098013	2,041224	1,3E-06	0,000255	Cd81
191,4026613	0,294463875	0,157188	2,03786	1,36E-06	0,000261	Calm1
4,050563558	0,046600981	0,043158	2,007419	1,94E-06	0,000356	Dpm3
9,343875261	0,092614973	0,063982	2,008769	1,91E-06	0,000356	Ran
4,050563558	0,046600981	0,043158	2,007419	1,94E-06	0,000356	Slc25a1
9,073168856	0,089923725	0,063215	2,004656	2,01E-06	0,000356	Gm26740
26,63685152	0,06122848	0,051799	2,004711	2,01E-06	0,000356	Eif1
6,197674782	0,074640833	0,055062	2,001677	2,08E-06	0,000361	Flt1
24,04008935	0,181329855	0,09959	2,000744	2,1E-06	0,000361	Cox4i1
3,706986221	0,042841533	0,041444	1,996563	2,21E-06	0,000373	Dancr
76,04322918	0,266817149	0,139219	1,988836	2,42E-06	0,000402	Gm28376
9,763441211	0,092993675	0,064777	1,981876	2,62E-06	0,00043	Tubb5
8,280374212	0,093880503	0,064988	1,967184	3,1E-06	0,000487	4921511C10Rik
17,24557333	0,055460702	0,049352	1,96747	3,09E-06	0,000487	Tmem126a
89,01553861	0,28255563	0,144755	1,966077	3,14E-06	0,000487	Psap
27,23790788	0,186251712	0,104631	1,96882	3,05E-06	0,000487	Bex2
136,742519	-0,245138599	0,153295	-1,95741	3,47E-06	0,000531	Chrm3
21,54821861	0,155288	0,091861	1,952306	3,68E-06	0,000551	Ndufb3
10,65413391	0,106300553	0,067676	1,95178	3,71E-06	0,000551	Tuba1b
433,2700701	-0,291970709	0,153176	-1,94606	3,96E-06	0,000579	Aff3

3,844477243	0,044318592	0,042126	1,944002	4,05E-06	0,000579	Ctxn1
3,80775708	0,04392703	0,041947	1,944893	4,01E-06	0,000579	Aldoc
383,4279832	-0,302970874	0,15552	-1,93493	4,49E-06	0,000634	Ldlrad4
19,41778804	0,126993325	0,082447	1,926428	4,94E-06	0,000681	H2afz
20,1908853	0,173643845	0,090558	1,926645	4,93E-06	0,000681	Gm36198
97,46423912	0,285139464	0,143999	1,917468	5,47E-06	0,000744	Ckb
733,4024319	-0,301743951	0,136969	-1,91348	5,72E-06	0,00076	Cdk8
1762,692582	-0,238512123	0,126751	-1,91349	5,72E-06	0,00076	Foxp1
10,19039946	-0,075557567	0,069068	-1,89472	7,06E-06	0,000926	Gm17167
2407,2662	-0,230077998	0,12393	-1,87645	8,64E-06	0,001121	Grm7
26,95005742	0,177248891	0,104396	1,871576	9,12E-06	0,001169	Fkbp2
29,48758786	-0,197702751	0,106685	-1,86377	9,93E-06	0,001259	Gpc5
38,03525986	0,217664619	0,115825	1,849192	1,17E-05	0,00146	C1qtnf4
31,48478464	0,191539691	0,110033	1,84061	1,28E-05	0,001585	Gm47423
4684,091465	-0,198751523	0,113867	-1,83294	1,39E-05	0,001704	Nrg1
48,37539102	0,083581056	0,063052	1,82706	1,48E-05	0,001795	Fndc9
477,3690552	0,297114008	0,152074	1,820115	1,6E-05	0,001914	Rapgef4
21,62922416	0,155504402	0,094356	1,815569	1,68E-05	0,001988	Atpif1
5,60501902	0,064321115	0,052631	1,81377	1,71E-05	0,002005	Polr2j
5,78656797	0,069285913	0,05573	1,812053	1,74E-05	0,002021	Guk1
11,75629758	0,108106775	0,075768	1,809408	1,79E-05	0,002057	Mrfap1
12,7499533	0,107548014	0,070276	1,804576	1,88E-05	0,002122	Chchd10
6,211247911	0,072185276	0,057778	1,804651	1,88E-05	0,002122	Maf1
13,71849218	0,119025766	0,078817	1,801435	1,95E-05	0,002173	Arf5
6,554769695	0,06388143	0,054431	1,794479	2,1E-05	0,002316	Pcbp1
1290,052632	-0,235889936	0,132502	-1,78772	2,25E-05	0,002463	Ryr3
2712,574494	-0,213979107	0,120123	-1,78412	2,34E-05	0,002534	Grm5
1453,705665	-0,233857516	0,134743	-1,77098	2,69E-05	0,002881	Opcml
2153,745628	-0,218974932	0,123623	-1,76892	2,74E-05	0,002915	Cacna2d3
1143,095338	-0,245769323	0,137883	-1,76691	2,8E-05	0,002949	Pde4b
6317,488738	-0,193024986	0,11262	-1,75712	3,1E-05	0,003235	Rbfox1
10,5500196	0,10025763	0,072578	1,746444	3,47E-05	0,003548	Ndufa6
54,2060271	-0,227677192	0,128441	-1,74643	3,47E-05	0,003548	Gm44593
6,74730185	0,068680936	0,056052	1,74218	3,62E-05	0,003642	Atp5d
538,819791	-0,267218968	0,149726	-1,74214	3,62E-05	0,003642	Mgat4c
24,96512193	0,135535586	0,091009	1,734502	3,92E-05	0,003905	Calm3
15,49382059	0,122037407	0,081413	1,732285	4,01E-05	0,003959	Atp5h
15,15045006	0,132539874	0,076203	1,729275	4,14E-05	0,004012	Ptgds
1423,065705	-0,23103525	0,130702	-1,72971	4,12E-05	0,004012	Erc2
597,7984977	0,261555393	0,148725	1,727031	4,23E-05	0,004035	Ube3a
16,19701705	0,128672283	0,086115	1,72774	4,2E-05	0,004035	Pgam1
62,63017238	0,215026392	0,134299	1,72129	4,49E-05	0,004243	Tmem30a
8,277312084	0,085469976	0,061272	1,719558	4,57E-05	0,004282	Atp5g1
48,40520474	0,223897216	0,125767	1,716823	4,7E-05	0,004366	D830024N08Rik
385,3393825	-0,283188857	0,154354	-1,7135	4,86E-05	0,004442	Egfem1
237,0132444	-0,319808447	0,157979	-1,71357	4,86E-05	0,004442	March1

468,1387275	-0,247516893	0,151559	-1,7096	5,05E-05	0,004547	Pard3
16,63714516	0,130847958	0,086239	1,709625	5,05E-05	0,004547	Pde6a
3952,768408	-0,194092876	0,119386	-1,70331	5,39E-05	0,004764	Kcnd2
179,7432039	-0,250251216	0,1571	-1,70275	5,42E-05	0,004764	Mpp6
454,1513649	-0,258088933	0,1521	-1,70246	5,43E-05	0,004764	Pde1c
10,15044642	0,100535576	0,071098	1,701859	5,47E-05	0,004764	Ndn
3065,259788	-0,2103631	0,119209	-1,69813	5,68E-05	0,004908	Lsamp
23,3756353	0,054065666	0,049724	1,695251	5,84E-05	0,004938	Ndufa4
1030,39615	-0,236030541	0,136888	-1,69571	5,82E-05	0,004938	Gabrg3
515,3982668	-0,249222574	0,150472	-1,69676	5,75E-05	0,004938	Rgs7bp
527,7482678	-0,251490615	0,149847	-1,69265	6E-05	0,00503	Enox1
6,923681257	0,074599546	0,059485	1,691504	6,07E-05	0,005051	Mbd3
482,5237589	-0,261012253	0,151069	-1,68139	6,71E-05	0,005548	Cdh8
39,92875495	0,182160509	0,118736	1,680239	6,79E-05	0,005571	Ptma
57,61942202	-0,218845158	0,132048	-1,67233	7,35E-05	0,00594	Slc24a3
24,83333205	0,167778187	0,100819	1,672406	7,34E-05	0,00594	Gm15398
122,0733334	0,278781657	0,152207	1,671467	7,41E-05	0,005948	P2ry14
278,5046794	0,266575982	0,157371	1,669876	7,53E-05	0,005999	Galnt18
316,9725276	-0,268039365	0,156454	-1,64541	9,58E-05	0,007582	Thrb
40,82195431	0,196985761	0,119935	1,642438	9,87E-05	0,007751	Pdlim7
290,8553916	0,259324637	0,157152	1,640356	0,000101	0,007855	Fgfr2
25,6325167	0,151079506	0,102655	1,638602	0,000102	0,007935	Nefl
6,270184617	0,072549973	0,05797	1,636622	0,000104	0,008034	Btg2
2526,405837	-0,19585928	0,121573	-1,63196	0,000109	0,008127	Adgrb3
692,1842983	-0,243789774	0,144897	-1,6323	0,000109	0,008127	Cacna1e
1579,191806	-0,216432595	0,128676	-1,63234	0,000109	0,008127	Csmd3
535,2846933	-0,252093654	0,149714	-1,63467	0,000106	0,008127	Adcy5
7,788544861	0,072251564	0,05891	1,63199	0,000109	0,008127	Ndufa1
500,7118104	-0,211043504	0,116131	-1,62945	0,000112	0,008272	Lars2
1247,368341	-0,217948635	0,135827	-1,61685	0,000126	0,009283	Grid2
12,40756539	0,11046974	0,070678	1,607335	0,000139	0,010106	Rabac1
286,7463459	-0,264519212	0,15736	-1,60463	0,000142	0,010304	Sorcs3
6,444395801	0,074667971	0,058629	1,603854	0,000143	0,010314	Cycs
4,991035711	0,057162524	0,050074	1,602108	0,000146	0,010354	Tomm7
9,807971487	0,097999423	0,069667	1,602447	0,000145	0,010354	Ap2m1
5,601540241	0,06447743	0,055383	1,600869	0,000147	0,010397	Coq8a
2125,332495	-0,191886411	0,123682	-1,60035	0,000148	0,010397	Ank3
420,2288277	-0,249961774	0,15323	-1,59768	0,000152	0,010598	Strn
4,239505828	0,047500098	0,046338	1,591757	0,000161	0,011144	Nsmce3
28,74022306	0,156394509	0,107022	1,589118	0,000165	0,011357	Pcdh19
2220,980404	-0,186676569	0,126284	-1,58132	0,000178	0,012152	Auts2
454,0331287	-0,257865902	0,152253	-1,57325	0,000192	0,013034	Gm26871
951,5280278	-0,2261615	0,145274	-1,56987	0,000198	0,013372	Lingo2
1390,176109	-0,207747305	0,132298	-1,56874	0,0002	0,013434	Magi2
261,3785771	-0,242424768	0,157638	-1,56695	0,000203	0,013579	D430041D05Rik
98,10186819	0,209386839	0,144602	1,564433	0,000208	0,01382	Hsp90aa1

140,9114765	-0,239833215	0,154027	-1,56085	0,000215	0,014123	Gm32647
37,36159322	-0,142311435	0,116608	-1,56088	0,000215	0,014123	Lrrc3b
9,827983221	0,088103307	0,068231	1,558613	0,00022	0,014337	Cox17
6,16486916	0,071227907	0,057576	1,556334	0,000224	0,014392	Tm2d2
207,1746466	-0,256432578	0,157769	-1,5571	0,000223	0,014392	Zfhx3
40,6199595	0,167196817	0,119228	1,556401	0,000224	0,014392	8030451O07Rik
6,06678248	-0,035445664	0,055302	-1,55518	0,000227	0,014464	Blvra
47,18333638	-0,181918256	0,125302	-1,55372	0,00023	0,014579	Synpo2
111,7340592	0,233670625	0,146454	1,549693	0,000239	0,01505	Serinc1
970,463047	-0,216592123	0,140716	-1,54757	0,000244	0,015263	Slit3
44,46967604	0,181270498	0,12187	1,545584	0,000248	0,01546	Arl6ip1
2588,744723	-0,202368668	0,129362	-1,54397	0,000252	0,015605	Ptprd
15,21688853	-0,132554306	0,084842	-1,5392	0,000263	0,016219	Gm50370
104,5048112	-0,227417915	0,149335	-1,53846	0,000265	0,016239	Gm2164
2095,174311	-0,19183077	0,124783	-1,5315	0,000283	0,017221	Mdga2
81,47727252	-0,220200827	0,143091	-1,52889	0,000289	0,017452	Iqgap2
3,659449513	0,044835229	0,043817	1,528854	0,000289	0,017452	Gm20616
219,0447124	-0,250941911	0,157843	-1,52824	0,000291	0,017457	Khdrbs2
28,61190301	0,151073016	0,104206	1,52418	0,000302	0,01801	Ywhah
616,0606251	-0,222359478	0,147196	-1,52366	0,000304	0,01801	Grid1
24,96051347	0,132037061	0,095473	1,518446	0,000318	0,018789	4430402I18Rik
8,412770211	0,071197841	0,060114	1,514857	0,000329	0,019311	Ndufb10
112,8960828	0,226986699	0,15026	1,511931	0,000338	0,019728	Plp1
14,61909004	0,107058138	0,082898	1,511356	0,00034	0,019728	Nat8l
11,97479416	0,097168878	0,075374	1,509969	0,000344	0,019772	Fbl
10,00392898	0,00449977	0,0694	-1,51015	0,000343	0,019772	Jmjd6
5776,955663	-0,172874059	0,116726	-1,50622	0,000356	0,020351	Celf2
1069,423158	-0,196262655	0,136504	-1,5017	0,000371	0,02109	Gpr158
143,3009273	-0,218335213	0,154627	-1,4974	0,000385	0,021484	Bach2
522,1994224	-0,221459654	0,150728	-1,49828	0,000382	0,021484	Pcdh7
10,33670663	0,079665288	0,064534	1,496858	0,000387	0,021484	Vamp2
5,668708618	0,061833414	0,055659	1,498697	0,000381	0,021484	Prrg3
6,88558973	0,072985228	0,060168	1,497253	0,000386	0,021484	Magee1
29,56012617	0,13974387	0,105009	1,494305	0,000396	0,021874	Rogdi
1914,021759	-0,186037423	0,125641	-1,49357	0,000399	0,02191	Dcc
11,14404162	0,100988682	0,07374	1,489938	0,000412	0,022525	Adam11
2523,264489	-0,188641897	0,122062	-1,48592	0,000427	0,023237	Nkain2
9,654402238	0,081245444	0,068435	1,482938	0,000439	0,023749	Rab3a
4,771230692	-0,042473613	0,050153	-1,48083	0,000447	0,024082	2900009J06Rik
125,2620591	0,222371172	0,151986	1,473906	0,000475	0,02549	Hsp90ab1
22,04284297	0,128193606	0,096343	1,469622	0,000494	0,026351	Gm27032
9,888541283	0,0972166	0,067507	1,468753	0,000498	0,026428	Gm48960
8,689809371	0,076908613	0,063488	1,467423	0,000503	0,026614	Serf2
21,61239966	0,131785005	0,096532	1,465442	0,000512	0,026762	Gm34086
29,04053933	0,154685647	0,107379	1,465839	0,000511	0,026762	Emc10
1474,947579	-0,192817293	0,137387	-1,4652	0,000513	0,026762	Ntm

2117,691889	-0,18157254	0,123913	-1,45737	0,00055	0,02854	Lrrc7
75,52494145	0,211271544	0,14039	1,45448	0,000564	0,029139	Itm2c
18,48892928	0,110142919	0,081657	1,451968	0,000577	0,029651	Dynll1
74,41235672	0,200992387	0,140077	1,442248	0,000628	0,032132	Gm28375
391,7328352	0,221733528	0,154556	1,438451	0,000649	0,032999	9630028H03Rik
6,001554859	-0,029544189	0,054205	-1,43814	0,000651	0,032999	Srf
103,0808496	0,191372452	0,148592	1,437343	0,000655	0,033077	Gm15261
34,81008275	-0,16282363	0,113555	-1,42662	0,000719	0,035963	Tanc1
117,0597702	0,211101203	0,151389	1,426605	0,000719	0,035963	Chn2
60,57530816	-0,112289578	0,081158	-1,42612	0,000722	0,035963	Gm19951
584,3166402	-0,220538795	0,148502	-1,42369	0,000737	0,036561	Xkr4
23,73910781	-0,132390927	0,099064	-1,42087	0,000756	0,037294	Phex
370,4365241	-0,222192005	0,154809	-1,41887	0,000769	0,037776	Unc5c
143,7940888	0,22186012	0,153425	1,407956	0,000844	0,041292	Syt11
670,9295325	-0,208890962	0,146113	-1,40595	0,000859	0,041821	Tnik
51,82897546	0,167082633	0,127623	1,397272	0,000924	0,044654	Pkm
41,80981561	0,161017315	0,118446	1,397215	0,000925	0,044654	Atp5b
834,5595908	-0,190130257	0,14207	-1,39519	0,000941	0,045036	Caln1
207,8269099	-0,233109546	0,157723	-1,39563	0,000937	0,045036	Slc8a1
20,53389071	-0,122501371	0,095179	-1,39387	0,000951	0,045348	Slc4a8
47,94313147	0,060019334	0,056954	1,391294	0,000972	0,046149	Actb
56,04939089	0,096243163	0,075964	1,39009	0,000982	0,046422	Aplp1
133,9854809	-0,190561195	0,153799	-1,38931	0,000989	0,046534	Pld5
47,52798811	0,177355518	0,122784	1,382709	0,001045	0,048779	Reep3
6,720443663	0,070778343	0,059628	1,382785	0,001044	0,048779	Golga7b
77,37125574	0,204514599	0,141089	1,381464	0,001056	0,049086	Itm2b
2559,95585	-0,162700767	0,121367	-1,38068	0,001063	0,049203	Kcnq5
100,3323705	0,207248481	0,145127	1,37761	0,001091	0,049697	Tac1
341,3073002	-0,231087555	0,15578	-1,37753	0,001092	0,049697	Cadm1
213,1201909	0,214251931	0,157816	1,378973	0,001078	0,049697	Gm20275
1016,057017	-0,19386158	0,137157	-1,37832	0,001084	0,049697	Ctnnd2

Suppl. Table 3: Gene Signatures

scRNA Aging Signature: Aging Signature; scRNA PD signature: Parkinson's Disease signature; scRNA PD Beginning: Genes that are changed due to alpha-syn at 6-months and keep being changed until 24-months; bProtein Aging Signature: Aging signature from Mass Spectrometry; bProtein PD Signature: Parkinson's Disease signature from Mass Spectrometry

scRNA Aging Signature	scRNA PD Signature	scRNA PD Beginning	bProtein Aging Signature	bProtein PD Signature
1700024B18Rik	1810037I17Rik	Adcy5	Akr1b7	Alg2
2410006H16Rik	4430402I18Rik	Anks1b	Anxa3	Atp1a1
4430402I18Rik	4921511C10Rik	Dgkb	Dhdh	Atp1a2
AC149090.1	Acp1	Gm26740	Gpt	Atp1a3
Acp1	Actb	Pde10a	Lrrc47	Atp1b1
Actb	Actg1	Pde7b	Myh10	Atp2a2

Actg1	Aplp1	S1/S2 (Transgene)	Pfdn5	Atp2b1
Amy1	Arf5	Sntg1	Slc27a4	Cdkn1b
Arf5	Atp6v0c			Faah
Atp5g1	Atp6v0e2			Flii
Atp5g3	Atpif1			Ina
Atp5h	AY036118			Lrrc47
Atp5j	Bach2			Myh10
Atp5j2	Bex2			Myh9
Atp5k	Btg2			Myo18a
Atp5md	Cacna1e			Nebl
Atp5mpl	Cacna2d3			Nefh
Atp6v0c	Calm1			Nefl
Atpif1	Calm3			Npm1
BC00e965	Camk1d			Nptn
Bc1	Cd81			Prkar2a
Calm3	Cdk8			Rpl34
Ccdc85b	Chchd10			Snrpd1
Cd63	Chchd2			Thy1
Cd81	Ckb			Tpd52
Celf3	Cntnap2			
Cfl1	Cox4i1			
Chchd10	Csmd3			
Chchd2	D830024N08Rik			
Chpf2	Dbi			
Chrac1	Dnase1l3			
Ckb	Dynll1			
Cox14	Edf1			
Cox4i1	Eif1			
Cox5a	Fgfr2			
Cox6c	Flt1			
Cox7a2	Fndc9			
Cox8a	Foxp1			
Cycs	Fth1			
Dusp1	Gabrg3			
Dynll1	Gm11290			
Egr4	Gm12027			
Eif1	Gm15398			
Eif1b	Gm15478			
Erbb4	Gm20616			
Fndc9	Gm20642			
Fth1	Gm2164			
Gapdh	Gm26871			
Gm13389	Gm28376			
Gm13889	Gm32647			
Gm15478	Gm36198			

Gm19951	Gm48960			
Gm32250	Grid2			
Gm32647	Grm5			
Gm36198	H2afz			
Gm47689	Ier5			
Gm484960	Kcnd2			
Grcc10	Kcnq5			
Grm3	Khdrbs2			
Grm8	Lars2			
H2afz	Lrrc7			
Hint1	Lsamp			
Hmbs	Ly6h			
Hras	Magee1			
Hsbp1	March1			
Hspa8	Mgat4c			
Junb	Mrfap1			
Jund	Ndufb10			
Kcnq1ot1	Ndufb3			
Krt12	Nkain2			
Krtcap2	Nrg1			
Luzp2	P2ry14			
Ly6h	Pard3			
Mettl26	Pde1c			
Micos13	Pde6a			
Myl6	Pebp1			
Ndn	Phactr1			
Ndufa4	Plp1			
Ndufa5	Prkg1			
Ndufb10	Psap			
Ndufb4	Ptgds			
Ndufb5	Rapgef4os1			
Ndufc1	Rpgrip1			
Ndufs5	Selenok			
Ndufs6	Slc24a5			
Nme1	Sorcs3			
Nme2	Strn			
Nop10	Tenm2			
Nudfa1	Timm10b			
Nudfa8	Tmem126a			
Oaz1	Tmem30a			
Pdlim7	Tuba1b			
Pdxp	Tubb2a			
Pfdn5	Tubb5			
Ppia	Ubb			
Prr5l	Ubc			
Qk	Ube3a			

Rab3a	Uqcrb			
Rabac1	Vamp2			
Ran	Xkr4			
Selenok	Zbtb20			
Serf2	Zfhx3			
Slc24a5				
Slc25a1				
Slc25a4				
Slc50a1				
Smpd1				
Sox2ot				
Spata16				
St18				
Thy1				
Timm8b				
Tm2d2				
Tmem160				
Tmem191c				
Tmem243				
Tmsb4x				
Tomm7				
Tpt1				
Tuba1a				
Tuba4a				
Tubb2a				
Tubb5				
Ubb				
Ubc				
Unc50				
Uqcc2				
Uqcrb				
Vamp2				
Vps28				
Zbtb20				

Suppl. Table 4: DEGs from all comparisons from Mass Spectrometry analysis

name: Gene symbol; ID: Protein ID; xy_p.val: p-value from the respecty xy condition; xy_p.adj: Benjamini Hochberg adjusted p-value from the respecty xy condition; xy_significant: Boolean statement if the p.adj was below the significance threshold of 0.05; xy_ratio: ratio reported by DEP; xy_centered: center reported by DEP

name	ID	ON24M_vs_OFF24M_p.val	ON24M_vs_OFF24M_p.adj	ON24M_vs_OFF24M_significant	ON24M_vs_OFF24M_ratio	OFF24M_centered	ON24M_centered
Alg2	Q9DBE8	1,87726E-05	0,022	WAHR	0,031	-0,0314	-0,0032

Atp1a1	Q8VDN2	2,92175E-05	0,0299	WAHR	0,0561	-0,0424	0,011
Atp1a2	Q6PIE5	9,9568E-05	0,0776	WAHR	0,108	-0,0913	0,0139
Atp1a3	Q6PIC6	1,48304E-06	0,00212	WAHR	0,0578	-0,0502	0,00751
Atp1b1	P14094	2,24047E-05	0,025	WAHR	0,0602	-0,0525	0,00661
Atp2a2	O55143	1,4448E-05	0,018	WAHR	0,0207	-0,0196	0,00124
Atp2b1	G5E829	8,09363E-05	0,0678	WAHR	0,0835	-0,0661	0,0169
Atp2b2	Q9R0K7	8,67248E-05	0,071	WAHR	0,0954	-0,0656	0,023
Cadm2	Q8BLQ9-3	1,0137E-07	0,0000993	WAHR	0,175	-0,138	0,0364
Cdkn1b	P46414	8,38525E-07	0,00107	WAHR	0,0356	-0,031	0,00393
Cops8	Q8VBV7	0,000115076	0,0846	WAHR	0,0164	-0,0141	0,00178
Crk	Q64010	1,43323E-05	0,0179	WAHR	-0,0163	0,0165	0,00082
Crtc1	Q68ED7	0,000133661	0,0937	WAHR	0,0277	-0,0235	0,00514
Esyt1	Q3U7R1	7,90575E-06	0,0113	WAHR	0,0987	-0,0919	0,00908
Faah	O08914	0,000118693	0,0862	WAHR	0,0273	-0,0229	0,0052
Flii	Q9JJ28	4,9893E-06	0,00778	WAHR	0,0391	-0,0396	0,000374
Gnai2	P08752	0,000118718	0,0862	WAHR	0,0438	-0,0338	0,00882
Gpm6a	P35802	0,000118179	0,0859	WAHR	0,0883	-0,0718	0,0152
Hnrnpf	Q9Z2X1	2,29576E-05	0,0254	WAHR	0,0482	-0,0379	0,011
Ina	P46660	1,51709E-05	0,0186	WAHR	0,102	-0,101	0,00279
Lrrc47	Q505F5	3,30963E-07	0,000367	WAHR	0,0207	-0,0214	-0,00166
Myh10	Q61879	3,91081E-08	0,0000303	WAHR	0,0238	-0,0253	-0,000552
Myh9	Q8VDD5	3,71953E-07	0,000402	WAHR	0,0256	-0,0219	0,00446
Myl12b	Q3THE2	2,62966E-05	0,0279	WAHR	0,0172	-0,014	0,00411
Myo18a	Q9JMH9-6	1,81133E-07	0,000193	WAHR	0,0247	-0,0183	0,00617

Nebl	Q9DC07	3,53289E-06	0,00552	WAHR	0,0337	-0,0329	0,000361
Nefh	P19246	1,76311E-06	0,00253	WAHR	0,128	-0,134	-0,000984
Nefl	P08551	1,3471E-05	0,0172	WAHR	0,151	-0,148	0,00402
Npm1	Q61937	6,37224E-07	0,000802	WAHR	0,026	-0,0206	0,00588
Nptn	P97300	7,14282E-05	0,0621	WAHR	0,0646	-0,053	0,0128
Pfdn6	Q03958	8,16749E-05	0,0682	WAHR	-0,0255	0,00459	-0,0201
Ppp1r9b	Q6R891	2,11056E-05	0,024	WAHR	0,0592	-0,0341	0,0239
Prkar2a	P12367	2,82552E-06	0,00439	WAHR	-0,0197	0,029	0,00985
Rabgap1l.1	A6H6A9-3	1,00948E-05	0,0138	WAHR	-0,0284	0,00782	-0,0189
Rpl34	Q9D1R9	7,13806E-06	0,0105	WAHR	0,0304	-0,0264	0,0052
Rplp1	P47955	6,05828E-05	0,055	WAHR	0,0307	-0,0219	0,0116
Serbp1	Q9CY58-2	7,31544E-06	0,0107	WAHR	0,0275	-0,0282	0,00101
Slc1a3	P56564	4,99647E-05	0,0472	WAHR	0,231	-0,162	0,0575
Snrpd1	P62315	7,19079E-05	0,0624	WAHR	0,0325	-0,0206	0,00971
Sptbn1	Q62261	4,09204E-05	0,0402	WAHR	0,0262	-0,0173	0,00828
Srsf1	Q6PDM2-3	2,7136E-05	0,0285	WAHR	0,0839	-0,0606	0,0257
Thy1	P01831	4,51274E-05	0,0436	WAHR	0,059	-0,0488	0,00864
Tpd52	Q62393-3	3,98473E-05	0,0393	WAHR	-0,0237	0,0166	-0,00476
Vps13c	Q8BX70-2	8,40779E-06	0,0118	WAHR	-0,0312	0,0107	-0,0202
**name**	**ID**	**lateON24M_vs_OFF24M_p.val**	**lateON24M_vs_OFF24M_p.adj**	**lateON24M_vs_OFF24M_significant**	**lateON24M_vs_OFF24M_ratio**	**lateON24M_centered**	**OFF24M_centered**
Alg2	Q9DBE8	1,91306E-07	0,00445	WAHR	0,0444	0,00756	-0,0314
Atp1a1	Q8VDN2	1,2401E-05	0,0851	WAHR	0,0623	0,0146	-0,0424

Atp1a2	Q6PIE5	1,55811E-05	0,09	WAHR	0,13	0,0342	-0,0913
Atp1a3	Q6PIC6	5,20234E-07	0,0104	WAHR	0,0645	0,0141	-0,0502
Atp1b1	P14094	6,17667E-06	0,067	WAHR	0,069	0,0144	-0,0525
Atp2a2	O55143	9,25793E-06	0,078	WAHR	0,0223	0,00297	-0,0196
Atp2b1	G5E829	1,82664E-05	0,0949	WAHR	0,0981	0,0309	-0,0661
Cdkn1b	P46414	1,47589E-05	0,0889	WAHR	0,0301	-0,00229	-0,031
Ckb	Q04447	1,58486E-05	0,0904	WAHR	-0,0113	-0,000107	0,0114
Dld	O08749	1,27507E-05	0,0857	WAHR	0,0256	0,00609	-0,0185
Faah	O08914	1,44203E-06	0,0263	WAHR	0,04	0,0187	-0,0229
Flii	Q9JJ28	7,66556E-06	0,073	WAHR	0,0393	0,00151	-0,0396
Ina	P46660	1,6188E-07	0,00393	WAHR	0,146	0,0485	-0,101
Lrrc47	Q505F5	3,01665E-09	0,0000342	WAHR	0,0287	0,00531	-0,0214
Lsm14a	Q8K2F8	1,20374E-05	0,0844	WAHR	-0,0323	-0,0245	0,00768
Map1b	P14873	1,92434E-05	0,0965	WAHR	0,0145	0,00293	-0,0119
Mettl13	Q91YR5	7,81099E-06	0,0736	WAHR	0,0335	0,0118	-0,0211
Myh10	Q61879	3,15025E-07	0,00709	WAHR	0,0216	-0,00167	-0,0253
Myh9	Q8VDD5	3,98519E-07	0,00851	WAHR	0,0264	0,00616	-0,0219
Myo18a	Q9JMH9-6	1,14932E-05	0,0834	WAHR	0,0192	0,000536	-0,0183
Ncbp1	Q3UYV9	1,55079E-05	0,0899	WAHR	0,0412	0,0278	-0,0108
Nebl	Q9DC07	5,17682E-06	0,0618	WAHR	0,034	0,000309	-0,0329
Nefh	P19246	7,60582E-08	0,00188	WAHR	0,163	0,0397	-0,134
Nefl	P08551	6,28074E-07	0,0118	WAHR	0,196	0,0489	-0,148
Nefm	P08553	3,94752E-06	0,0538	WAHR	0,104	0,0352	-0,0752
Npm1	Q61937	6,87137E-06	0,07	WAHR	0,0229	0,00319	-0,0206
Nptn	P97300	9,58854E-06	0,0789	WAHR	0,0789	0,0283	-0,053

Nucks1	Q80XU3	8,72667E-06	0,0765	WAHR	0,0455	0,0284	-0,0135
Ppid	Q9CR16	7,96435E-06	0,0741	WAHR	-0,0128	-0,0033	0,00643
Prkar2a	P12367	4,77713E-08	0,00151	WAHR	-0,0268	0,00327	0,029
Prkar2b	P31324	1,19394E-05	0,0842	WAHR	-0,0236	-0,00749	0,0166
Rpl34	Q9D1R9	1,34317E-05	0,0869	WAHR	0,0301	0,00615	-0,0264
Rplp2	P99027	4,40656E-06	0,0571	WAHR	0,0466	0,0136	-0,0312
Snrpd1	P62315	7,89801E-06	0,0739	WAHR	0,0403	0,0154	-0,0206
Thy1	P01831	1,41788E-05	0,0881	WAHR	0,0673	0,0153	-0,0488
Tpd52	Q62393-3	2,62208E-06	0,0418	WAHR	-0,0303	-0,00903	0,0166
Tstd3	Q9D0B5	9,00466E-06	0,0773	WAHR	-0,0409	-0,0121	0,0272
**name**	**ID**	**ON16M_vs_OFF16M_p.val**	**ON16M_vs_OFF16M_p.adj**	**ON16M_vs_OFF16M_significant**	**ON16M_vs_OFF16M_ratio**	**OFF16M_centered**	**ON16M_centered**
Ints3	Q7TPD0	0,000115335	0,0291	WAHR	0,0261	-0,00856	0,0181
Prune2	Q52KR3	0,000348663	0,0978	WAHR	0,0236	-0,0128	0,009
**name**	**ID**	**lateON16M_vs_OFF16M_p.val**	**lateON16M_vs_OFF16M_p.adj**	**lateON16M_vs_OFF16M_significant**	**lateON16M_vs_OFF16M_ratio**	**lateON16M_centered**	**OFF16M_centered**
Acadsb	Q9DBL1	7,11836E-05	0,0693	WAHR	-0,0255	-0,00704	0,0183
Ensa	P60840	3,01292E-05	0,0403	WAHR	-0,0314	-0,0143	0,0104
Urm1	Q9D2P4	2,55326E-06	0,00129	WAHR	0,0432	0,0286	-0,0195
Znf207	Q9JMD0-4	6,27297E-05	0,0655	WAHR	0,0217	0,0122	-0,00643
**name**	**ID**	**ON6M_vs_OFF6M_p.val**	**ON6M_vs_OFF6M_p.adj**	**ON6M_vs_OFF6M_significant**	**ON6M_vs_OFF6M_ratio**	**OFF6M_centered**	**ON6M_centered**
Ak4	Q9WUR9	3,35881E-05	0,0869	WAHR	-0,0348	0,00358	-0,0137
Akr1b7	P21300	9,32172E-09	0,000000185	WAHR	0,0905	-0,0415	-0,00135

Btbd17	Q9DB72	4,06498E-05	0,0936	WAHR	0,06	-0,00953	0,0393
Gabarapl1	Q8R3R8	4,68E-05	0,0981	WAHR	0,0332	-0,0272	0,00115
Glrx	Q9QUH0	4,06868E-05	0,0936	WAHR	-0,0305	0,00142	-0,0134
Golph3	Q9CRA5	8,46434E-07	0,00124	WAHR	0,0556	-0,0199	0,0193
**name**	**ID**	**OFF24M_vs_OFF6M_p.val**	**OFF24M_vs_OFF6M_p.adj**	**OFF24M_vs_OFF6M_significant**	**OFF24M_vs_OFF6M_ratio**	**OFF24M_centered**	**OFF6M_centered**
Akr1b7	P21300	9,96763E-08	0,0323	WAHR	0,0783	-0,0135	-0,0415
Alg2	Q9DBE8	5,95366E-07	0,0816	WAHR	-0,0497	-0,0314	0,00337
Anxa3	O35639	9,08992E-10	0,000277	WAHR	0,044	0,012	0,0123
Atp2a2	O55143	6,332E-08	0,0261	WAHR	-0,0377	-0,0196	-0,00197
Ca2	P00920	4,3898E-07	0,0704	WAHR	0,0215	0,0141	-0,0243
Crtc1	Q68ED7	2,68794E-07	0,0533	WAHR	-0,0552	-0,0235	0,00131
D3Ertd751e	Q8BGN2	1,56689E-07	0,0376	WAHR	0,0533	0,0238	0,00358
Dhdh	Q9DBB8	7,78757E-07	0,0912	WAHR	0,0372	0,0108	-0,00873
Fah	P35505	7,07107E-07	0,0878	WAHR	0,0223	0,0106	-0,0142
Gpt	Q8QZR5	4,94301E-10	0,000205	WAHR	0,0493	0,0239	-0,00304
Gstm7	Q80W21	1,03289E-07	0,0327	WAHR	0,0339	0,0115	-0,00691
Lrrc47	Q505F5	2,85063E-07	0,0552	WAHR	-0,0262	-0,0214	0,0021
Myh10	Q61879	7,86457E-07	0,0915	WAHR	-0,0245	-0,0253	0,0102
Nutf2	P61971	1,46192E-07	0,0368	WAHR	0,0386	0,0161	0,00503
Pfdn5	Q9WU28	1,59085E-07	0,0377	WAHR	0,043	0,0282	-0,0366
Ppt1	O88531	6,5133E-09	0,00322	WAHR	0,0668	0,0363	-0,0395
Psip1	Q99JF8	8,95967E-07	0,0969	WAHR	0,0378	0,0106	0,000351
Slc27a4	Q91VE0	3,86891E-07	0,0659	WAHR	-0,0475	-0,016	0,00786
Tppp	Q7TQD2	1,46165E-07	0,0368	WAHR	0,0274	0,0153	-0,015

**name**	**ID**	**OFF24M_vs_OFF16M_p.val**	**OFF24M_vs_OFF16M_p.adj**	**OFF24M_vs_OFF16M_significant**	**OFF24M_vs_OFF16M_ratio**	**OFF16M_centered**	**OFF24M_centered**
Cadm2	Q8BLQ9-3	2,37742E-06	0,0712	WAHR	-0,152	0,0135	-0,138
Gpt	Q8QZR5	1,67483E-08	0,000666	WAHR	0,0344	-0,0112	0,0239
Ina	P46660	9,82097E-08	0,00597	WAHR	-0,155	0,0551	-0,101
Lrrc47	Q505F5	1,56872E-08	0,000653	WAHR	-0,0268	0,0048	-0,0214
Mtpn	P62774	3,22635E-07	0,0168	WAHR	0,0259	-0,011	0,0155
Myh10	Q61879	9,23191E-09	0,000521	WAHR	-0,0278	0,00312	-0,0253
Myh9	Q8VDD5	5,33921E-07	0,0263	WAHR	-0,0267	0,00536	-0,0219
Nefh	P19246	1,17297E-08	0,000587	WAHR	-0,189	0,0582	-0,134
Nefl	P08551	1,46347E-07	0,0086	WAHR	-0,222	0,0747	-0,148
Nefm	P08553	8,50578E-07	0,0384	WAHR	-0,119	0,0464	-0,0752
Pfdn5	Q9WU28	2,71898E-06	0,0755	WAHR	0,0304	-0,00244	0,0282
Prkar2a	P12367	1,20341E-09	0,0000413	WAHR	0,0344	-0,00499	0,029
Rabggta	Q9JHK4	1,27756E-06	0,0508	WAHR	-0,0219	0,0102	-0,0113
Serbp1	Q9CY58-2	2,62742E-06	0,0744	WAHR	-0,0318	0,0047	-0,0282
Snta1	Q61234	2,79455E-06	0,0764	WAHR	0,0356	-0,0137	0,0232
Tecpr1	Q80VP0	1,91586E-06	0,0641	WAHR	-0,0302	0,0131	-0,0169
Tom1	O88746	2,92649E-07	0,0157	WAHR	0,0291	-0,0111	0,0177
Tstd3	Q9D0B5	2,6672E-06	0,0749	WAHR	0,0461	-0,0183	0,0272

**name**	**ID**	**OFF16M_vs_OFF6M_p.val**	**OFF16M_vs_OFF6M_p.adj**	**OFF16M_vs_OFF6M_significant**	**OFF16M_vs_OFF6M_ratio**	**OFF16M_centered**	**OFF6M_centered**
Akr1b7	P21300	1,17154E-09	3,67E-08	WAHR	0,11	0,0188	-0,0415
Anxa3	O35639	9,07451E-06	0,0776	WAHR	0,0263	-0,00528	0,0123

Dak	Q8VC30	9,28808E-06	0,0781	WAHR	0,0347	0,0173	-0,0296
Dhdh	Q9DBB8	2,96005E-05	0,092	WAHR	0,0306	0,00341	-0,00873
Pfn1	P62962	2,59795E-06	0,0407	WAHR	-0,0202	-0,00454	-0,011
Rap1gds1	E9Q912	1,19013E-05	0,0861	WAHR	0,0163	0,00376	-0,00218
Rps15	P62843	2,00586E-05	0,0996	WAHR	0,0387	0,00601	-0,000349
Slc27a4	Q91VE0	6,19796E-06	0,068	WAHR	-0,0421	-0,0102	0,00786

Suppl. Table 5: Central Regulator data of networks in PD and aging

PD sign gene: gene symbols from the PD signature; pAdj: Benjamini Hochberg correcte p-values; PD sigRegulator: Categorization of the gene in the network. "Top20" means it is one of the 20 most central genes in the network, "Sig" means the gene is significantly more central than random control, and "nonSig" means not more central than random control; Age gene: Gene symbols from the aging signature; Age sigRegulator: see PD sigRegulator description; MitochondrialFunction: Boolean column if a gene was annotated in a KEGG term for mitochondrial function; Age no Mito gene: Gene symbols from the aging signature without mitochondrial function genes; Age no Mito sigRegulator: see PD sigRegulator description

PD sign gene	pAdj	PD sigRegulator		Age gene	pAdj	Age sigRegulator	MitochondrialFunction		Age no Mito gene	pAdj	Age no Mito sigRegulator
Ube3a	6,2985E-18	Top20		Atp5a1	6,6253E-28	Top40	Yes		Atp5e	1,7474E-24	Top40
Lrrc7	5,0203E-15	Top20		Cox6b1	1,142E-25	Top40	Yes		Ndufa11	1,9104E-19	Top40
Tubb5	2,9173E-14	Top20		Atp5h	1,326E-24	Top40	Yes		Tubb2a	7,9714E-17	Top40
Calm1	5,3828E-14	Top20		Cycs	1,5163E-24	Top40	Yes		Dynll1	6,3923E-16	Top40
Grm5	5,3828E-14	Top20		Atp5e	1,7474E-24	Top40	No		Atpif1	4,666E-15	Top40
Kcnq5	7,9255E-14	Top20		Atp5j2	2,1847E-24	Top40	Yes		Gapdh	9,8094E-15	Top40
Fgfr2	1,3135E-13	Top20		Ndufa4	2,78E-24	Top40	Yes		Ubc	1,0265E-14	Top40
Strn	1,5523E-13	Top20		Atp5b	6,4268E-24	Top40	Yes		Tubb5	5,2434E-14	Top40
Actb	1,5656E-13	Top20		Atp5g3	1,5408E-23	Top40	Yes		Actb	1,4985E-13	Top40
Dynll1	4,1402E-13	Top20		Cox4i1	7,446E-23	Top40	Yes		Hspa8	1,4985E-13	Top40
Pard3	4,8079E-13	Top20		Atp5g1	2,4219E-22	Top40	Yes		Cd63	1,9723E-13	Top40

Camk1d	2,3394E-12	Top20		Ndufab1	2,4219E-22	Top40	Yes		Actg1	2,7643E-13	Top40
Phactr1	1,16E-10	Top20		Ndufc1	1,1219E-21	Top40	Yes		Nme1	7,1155E-13	Top40
Cacna2d3	3,4336E-10	Top20		Ndufa5	1,8323E-21	Top40	Yes		Cadm1	7,2321E-13	Top40
Kcnd2	4,3919E-10	Top20		Ndufs6	2,4194E-21	Top40	Yes		Tuba4a	7,2321E-13	Top40
Cacna1e	6,4689E-10	Top20		Cox7a2	5,3047E-21	Top40	Yes		Erbb4	8,0986E-13	Top40
Nrg1	6,9416E-10	Top20		Ndufb4	6,4582E-21	Top40	Yes		Cox14	1,0626E-12	Top40
Ubb	1,2538E-09	Top20		Cox6c	9,3254E-21	Top40	Yes		Tuba1a	2,8953E-12	Top40
Acp1	1,8631E-09	Top20		Ndufa1	1,5215E-20	Top40	Yes		Tanc1	4,3783E-12	Top40
Dlg4	2,125E-09	Top20		Ndufb5	1,9487E-20	Top40	Yes		Ppia	5,1039E-12	Top40
Prkg1	3,8713E-09	Sig		Atp5j	6,2602E-20	Top40	Yes		Cfl1	5,3198E-12	Top40
Khdrbs2	1,1161E-08	Sig		Ndufs5	9,9857E-20	Top40	Yes		Ran	1,0675E-11	Top40
Cntnap2	1,6046E-08	Sig		Ndufa11	1,9104E-19	Top40	No		Oaz1	1,3862E-11	Top40
Cacna1c	3,0272E-08	Sig		Ndufb10	7,1989E-19	Top40	Yes		Gm10053	1,5461E-11	Top40
Zbtb20	5,4716E-08	Sig		Cox5a	1,1511E-18	Top40	Yes		Gtpbp6	1,5461E-11	Top40
Tenm2	2,2314E-07	Sig		Ndufa8	1,1511E-18	Top40	Yes		H2afz	3,0916E-11	Top40
Pde1c	1,1266E-06	Sig		Atp5md	1,3189E-18	Top40	Yes		Eif1	3,9138E-11	Top40
Dbi	1,9726E-06	Sig		Atp5d	4,4402E-18	Top40	Yes		Acp1	1,7166E-10	Top40
Gabrg3	3,6355E-06	Sig		mt-Nd1	1,0958E-17	Top40	Yes		Vamp2	2,1913E-10	Top40
Dlg2	3,8075E-06	Sig		Tubb2a	7,9714E-17	Top40	No		Dusp1	3,8781E-10	Top40
Foxp1	3,8075E-06	Sig		Chchd2	4,3348E-16	Top40	Yes		Ubb	7,4927E-10	Top40
Grid2	4,3593E-06	Sig		Dynll1	6,3923E-16	Top40	No		Pfdn5	1,441E-09	Top40
Atp10a	1,1243E-05	Sig		Atp5mpl	1,3353E-15	Top40	Yes		Fgf13	2,803E-09	Top40
Cacng2	3,1903E-05	Sig		Atpif1	4,666E-15	Top40	No		Calm3	3,3468E-09	Top40
Ubqln2	3,1903E-05	Sig		Gapdh	9,8094E-15	Top40	No		Rps28	7,1291E-09	Top40
Btg2	4,2737E-05	Sig		Ubc	1,0265E-14	Top40	No		Hmbs	7,1612E-09	Top40
Nlgn3	4,6245E-05	Sig		Tubb5	5,2434E-14	Top40	No		Tardbp	1,1911E-08	Top40

Bach2	5,7481E-05	Sig		Actb	1,4985E-13	Top40	No		Tpt1	1,2849E-08	Top40
Atp6v0c	7,1892E-05	Sig		Hspa8	1,4985E-13	Top40	No		Mrps12	1,8772E-08	Top40
Tuba4a	0,00010059	Sig		Atp6v0c	1,5931E-13	Top40	Yes		Serf2	2,8972E-08	Top40
Ap2m1	0,00018997	Sig		Cd63	1,9723E-13	Sig	No		Hint1	3,0068E-08	Sig
Tmem30a	0,00045861	Sig		Actg1	2,7643E-13	Sig	No		Myl6	3,0068E-08	Sig
Calm3	0,00080131	Sig		Tomm7	2,9649E-13	Sig	Yes		Hras	3,7591E-08	Sig
Dlg1	0,00081251	Sig		Nme1	7,1155E-13	Sig	No		Mapt	3,7591E-08	Sig
Csmd3	0,00109669	Sig		Cadm1	7,2321E-13	Sig	No		Flt3l	3,7844E-08	Sig
Gabra5	0,00109669	Sig		Tuba4a	7,2321E-13	Sig	No		Hprt	3,7844E-08	Sig
Llgl2	0,00109669	Sig		Erbb4	8,0986E-13	Sig	No		Eif1b	5,8254E-08	Sig
Mrfap1	0,00109669	Sig		Cox14	1,0626E-12	Sig	No		Magi2	6,6643E-08	Sig
Mob4	0,00128452	Sig		Tuba1a	2,8953E-12	Sig	No		Nme2	1,5195E-07	Sig
Oca2	0,00128452	Sig		Tanc1	4,3783E-12	Sig	No		Dync1i2	1,885E-07	Sig
Rpgrip1	0,00216862	Sig		Ppia	5,1039E-12	Sig	No		Cdh18	2,7448E-07	Sig
Egfr	0,00222777	Sig		Cfl1	5,3198E-12	Sig	No		Maml2	5,6088E-07	Sig
Rpl23	0,0033298	Sig		Ran	1,0675E-11	Sig	No		Eef1b2	6,7177E-07	Sig
Prnp	0,00353444	Sig		Oaz1	1,3862E-11	Sig	No		Khdrbs2	9,1018E-07	Sig
Adgrl1	0,00389979	Sig		Gm10053	1,5461E-11	Sig	No		Thy1	9,2512E-07	Sig

Zfhx3	0,00389979	Sig		Gtpbp6	1,5461E-11	Sig	No		Cd81	1,1023E-06	Sig
Gabrb3	0,00456178	Sig		Slc25a4	2,4722E-11	Sig	Yes		Ckb	1,2441E-06	Sig
Kcnab2	0,00456178	Sig		H2afz	3,0916E-11	Sig	No		Rab3a	1,3285E-06	Sig
Dlgap1	0,00626518	Sig		Eif1	3,9138E-11	Sig	No		Aurkaip1	1,6854E-06	Sig
Slc24a5	0,00664196	Sig		Pink1	1,4437E-10	Sig	Yes		Fau	1,915E-06	Sig
Nrxn1	0,01154258	Sig		Sdha	1,5793E-10	Sig	Yes		Grm8	2,6714E-06	Sig
Eps15	0,01262804	Sig		Chchd10	1,5822E-10	Sig	Yes		Pdxp	2,9595E-06	Sig
Snap25	0,01307646	Sig		Acp1	1,7166E-10	Sig	No		Junb	3,5333E-06	Sig
Areg	0,01352594	Sig		Vamp2	2,1913E-10	Sig	No		Eif5a	3,8343E-06	Sig
Morf4l1	0,01464955	Sig		Dusp1	3,8781E-10	Sig	No		Grm3	5,3808E-06	Sig
Chchd10	0,0157966	Sig		Ubb	7,4927E-10	Sig	No		Zbtb20	6,2795E-06	Sig
Satb2	0,01625616	Sig		Pfdn5	1,441E-09	Sig	No		Mbd3	1,1618E-05	Sig
Afdn	0,0261441	Sig		Coa6	1,6069E-09	Sig	Yes		Vcp	1,8208E-05	Sig
Htt	0,02662284	Sig		Fgf13	2,803E-09	Sig	No		Ndn	2,055E-05	Sig
Apoe	0,03157071	Sig		Calm3	3,3468E-09	Sig	No		Drd3	2,3218E-05	Sig
Cd86	0,04184159	Sig		Rps28	7,1291E-09	Sig	No		Gm11808	3,2397E-05	Sig
Plg	0,04437328	Sig		Hmbs	7,1612E-09	Sig	No		Tmem243	3,2397E-05	Sig
Ppp2ca	0,04864295	Sig		Tardbp	1,1911E-08	Sig	No		Rpl35	4,9552E-05	Sig

Hras	0,05324879	nonSig		Tpt1	1,2849E-08	Sig	No		Qk	6,1057E-05	Sig
Grb2	0,05928826	nonSig		Mrps12	1,8772E-08	Sig	No		Selenok	7,0794E-05	Sig
Atp6v0e2	0,06041432	nonSig		Serf2	2,8972E-08	Sig	No		Arl6ip1	0,00010204	Sig
Cdc42	0,06215587	nonSig		Hint1	3,0068E-08	Sig	No		Aco2	0,00013887	Sig
Pts	0,06237407	nonSig		Myl6	3,0068E-08	Sig	No		Mrps10	0,00018261	Sig
Fmr1	0,07807099	nonSig		Hras	3,7591E-08	Sig	No		Mrps14	0,00018261	Sig
Agt	0,08110736	nonSig		Mapt	3,7591E-08	Sig	No		Prr5l	0,00026422	Sig
Gabrb1	0,08573363	nonSig		Flt3l	3,7844E-08	Sig	No		Vps28	0,0003259	Sig
Kcnq1	0,08573363	nonSig		Hprt	3,7844E-08	Sig	No		Chrac1	0,00047406	Sig
Ppp1ca	0,10261315	nonSig		Eif1b	5,8254E-08	Sig	No		Eef1a1	0,00048607	Sig
Dcx	0,10912657	nonSig		Magi2	6,6643E-08	Sig	No		Amy1	0,00061202	Sig
Cacna1d	0,11780381	nonSig		Nme2	1,5195E-07	Sig	No		Pdlim7	0,00075732	Sig
Akt1	0,14031313	nonSig		Dync1i2	1,885E-07	Sig	No		H3f3b	0,00084856	Sig
Igf2r	0,1487545	nonSig		Cdh18	2,7448E-07	Sig	No		Trp53	0,00084856	Sig
Rpl32	0,16113744	nonSig		Chchd3	2,8793E-07	Sig	Yes		Prkg1	0,00092835	Sig
App	0,18383766	nonSig		Cox10	2,8793E-07	Sig	Yes		Eif3m	0,00106906	Sig
Fgf4	0,2026428	nonSig		Maml2	5,6088E-07	Sig	No		Gucy1a1	0,00106906	Sig

Camk2a	0,20626768	nonSig		Eef1b2	6,7177E-07	Sig	No		Hsbp1	0,00106906	Sig
Atf2	0,21032554	nonSig		Khdrbs2	9,1018E-07	Sig	No		Krt12	0,00106906	Sig
Ctnnb1	0,2246303	nonSig		Thy1	9,2512E-07	Sig	No		Myh11	0,00106906	Sig
Camk1	0,30104309	nonSig		Cd81	1,1023E-06	Sig	No		Txn1	0,00106906	Sig
Cdh5	0,30324581	nonSig		Ckb	1,2441E-06	Sig	No		Cct7	0,00112675	Sig
Nos1	0,30324581	nonSig		Rab3a	1,3285E-06	Sig	No		Nsmce3	0,00112675	Sig
Prmt1	0,30324581	nonSig		Aurkaip1	1,6854E-06	Sig	No		Cct5	0,0013169	Sig
F2	0,35937612	nonSig		Fau	1,915E-06	Sig	No		Rpl5	0,0015172	Sig
Map4k4	0,35937612	nonSig		Grm8	2,6714E-06	Sig	No		Arf5	0,00166979	Sig
Plcb1	0,3731491	nonSig		Pdxp	2,9595E-06	Sig	No		Slc24a5	0,00173664	Sig
Ulk1	0,3731491	nonSig		Junb	3,5333E-06	Sig	No		Gga3	0,00181247	Sig
Bmpr2	0,38939285	nonSig		Eif5a	3,8343E-06	Sig	No		Ap2m1	0,00197594	Sig
Lat	0,38939285	nonSig		Grm3	5,3808E-06	Sig	No		Ndufa13	0,0024195	Sig
Braf	0,42134416	nonSig		Zbtb20	6,2795E-06	Sig	No		Atp6v0d2	0,00280026	Sig
Atf3	0,4367421	nonSig		Atp5c1	1,1618E-05	Sig	Yes		Cct2	0,00280026	Sig
Ngf	0,4367421	nonSig		Mbd3	1,1618E-05	Sig	No		Cyct	0,00280026	Sig
Cblb	0,4432279	nonSig		Vcp	1,8208E-05	Sig	No		Gpx1	0,00280026	Sig

Hsp90aa1	0,45273562	nonSig		Ndn	2,055E-05	Sig	No		Rpl22	0,00280026	Sig
Tgfb1	0,45273562	nonSig		Drd3	2,3218E-05	Sig	No		Acta2	0,00298278	Sig
Bdnf	0,46011958	nonSig		Gm11808	3,2397E-05	Sig	No		Cd34	0,00298278	Sig
Zap70	0,46162724	nonSig		Tmem243	3,2397E-05	Sig	No		Prkacb	0,00298278	Sig
Creb1	0,46447453	nonSig		Rpl35	4,9552E-05	Sig	No		Rpl31	0,00335273	Sig
Ncor2	0,46447453	nonSig		Micos13	5,1129E-05	Sig	Yes		Tm2d2	0,00335273	Sig
Neurod1	0,49510385	nonSig		Qk	6,1057E-05	Sig	No		Ins1	0,00395653	Sig
Nfatc2	0,50371741	nonSig		Selenok	7,0794E-05	Sig	No		Mrpl20	0,00395653	Sig
Egf	0,51893787	nonSig		Arl6ip1	0,00010204	Sig	No		Rabac1	0,00395653	Sig
Nfkbia	0,57481891	nonSig		Park7	0,0001205	Sig	Yes		Ubqln2	0,00395653	Sig
Aurkb	0,6478166	nonSig		Aco2	0,00013887	Sig	No		Rpl4	0,00418324	Sig
Src	0,6478166	nonSig		Snca	0,00015074	Sig	Yes		Capzb	0,00466725	Sig
Plcg1	0,67382355	nonSig		Mrps10	0,00018261	Sig	No		Cct4	0,00466725	Sig
Bcl2	0,67798086	nonSig		Mrps14	0,00018261	Sig	No		Ubr5	0,00870109	Sig
Syk	0,67798086	nonSig		Prr5l	0,00026422	Sig	No		Cd38	0,00991013	Sig
Cbl	0,70525066	nonSig		Vps28	0,0003259	Sig	No		Snap25	0,01033436	Sig
Crebbp	0,73224287	nonSig		Chrac1	0,00047406	Sig	No		Cdh2	0,01106172	Sig

Pak1	0,74117957	nonSig		Eef1a1	0,00048607	Sig	No		Ago2	0,01409	Sig
Pik3ca	0,82741524	nonSig		Amy1	0,00061202	Sig	No		Adcy1	0,01467537	Sig
Cdk1	0,83291619	nonSig		Pdlim7	0,00075732	Sig	No		Dync1h1	0,01467537	Sig
Cdk2	0,83291619	nonSig		H3f3b	0,00084856	Sig	No		Pdcd6ip	0,01539827	Sig
Bcl3	0,83417752	nonSig		Trp53	0,00084856	Sig	No		Hsp90aa1	0,01615176	Sig
Abl1	0,83523864	nonSig		Prkg1	0,00092835	Sig	No		Rpl13a	0,01774279	Sig
Bcl11a	0,84600117	nonSig		Eif3m	0,00106906	Sig	No		Selenop	0,01867821	Sig
Prkaca	0,84600117	nonSig		Gucy1a1	0,00106906	Sig	No		Flot1	0,02050144	Sig
Rhoa	0,84600117	nonSig		Hsbp1	0,00106906	Sig	No		Agt	0,0206002	Sig
Rps6ka3	0,84600117	nonSig		Krt12	0,00106906	Sig	No		Grin2b	0,02105035	Sig
Fyn	0,87236482	nonSig		Myh11	0,00106906	Sig	No		Fmr1	0,02130126	Sig
Brca1	0,8729025	nonSig		Txn1	0,00106906	Sig	No		Pmel	0,02130126	Sig
Mitf	0,94854009	nonSig		Cct7	0,00112675	Sig	No		App	0,02148234	Sig
Actr10	1	nonSig		Nsmce3	0,00112675	Sig	No		Dhx9	0,02148234	Sig
Ar	1	nonSig		Cct5	0,0013169	Sig	No		Egr4	0,02148234	Sig
Atp5a1	1	nonSig		Dnajc11	0,00141299	Sig	Yes		Rpl11	0,02148234	Sig
Atp5b	1	nonSig		Rpl5	0,0015172	Sig	No		Kdm6a	0,0241584	Sig

Cttnbp2	1	nonSig		Arf5	0,00166979	Sig	No		Cnbp	0,0244993	Sig
Egr1	1	nonSig		Slc24a5	0,00173664	Sig	No		Mrpl36	0,0244993	Sig
Ep300	1	nonSig		Gga3	0,00181247	Sig	No		Oca2	0,0244993	Sig
Esr1	1	nonSig		Ap2m1	0,00197594	Sig	No		Polr1c	0,0244993	Sig
Fau	1	nonSig		Ndufa13	0,0024195	Sig	No		Unc50	0,0244993	Sig
Fos	1	nonSig		Atp6v0d2	0,00280026	Sig	No		Eno1	0,0277608 7	Sig
Foxp3	1	nonSig		Cct2	0,00280026	Sig	No		Dlg4	0,0282728 3	Sig
Gata3	1	nonSig		Cyct	0,00280026	Sig	No		Adam10	0,0373697 2	Sig
Gsk3b	1	nonSig		Gpx1	0,00280026	Sig	No		Glo1	0,0381485 6	Sig
Gucy1a1	1	nonSig		Rpl22	0,00280026	Sig	No		Pdcd5	0,0381485 6	Sig
Gucy2d	1	nonSig		Acta2	0,00298278	Sig	No		Bag4	0,0488879 3	Sig
Hif1a	1	nonSig		Cd34	0,00298278	Sig	No		Myo18a	0,0488879 3	Sig
Hnf4a	1	nonSig		Prkacb	0,00298278	Sig	No		Ap3b1	0,0532971 2	nonSig
Hprt	1	nonSig		Rpl31	0,00335273	Sig	No		Dctn1	0,0532971 2	nonSig
Ift88	1	nonSig		Tm2d2	0,00335273	Sig	No		Dynll2	0,0532971 2	nonSig
Ins1	1	nonSig		Bdnf	0,0034468	Sig	Yes		Gpx4	0,0532971 2	nonSig
Jun	1	nonSig		Ins1	0,00395653	Sig	No		Rpl28	0,0532971 2	nonSig

Lsamp	1	nonSig		Mrpl20	0,00395653	Sig	No		Rpl38	0,05329712	nonSig
Magee1	1	nonSig		Rabac1	0,00395653	Sig	No		Ank1	0,05415493	nonSig
Mapk1	1	nonSig		Ubqln2	0,00395653	Sig	No		Psma7	0,05415493	nonSig
March1	1	nonSig		Rpl4	0,00418324	Sig	No		Naca	0,05855498	nonSig
Mgat4c	1	nonSig		Capzb	0,00466725	Sig	No		Pigz	0,06422793	nonSig
Myc	1	nonSig		Cct4	0,00466725	Sig	No		Tcp1	0,06422793	nonSig
Myzap	1	nonSig		Ubr5	0,00870109	Sig	No		Hnrnpu	0,07707115	nonSig
Prkca	1	nonSig		Cd38	0,00991013	Sig	No		Pick1	0,08360791	nonSig
Sorcs3	1	nonSig		Snap25	0,01033436	Sig	No		Cask	0,08491156	nonSig
Tp53	1	nonSig		Cdh2	0,01106172	Sig	No		Arf1	0,09671603	nonSig
				Ago2	0,01409	Sig	No		Rpl8	0,09671603	nonSig
				Adcy1	0,01467537	Sig	No		Mapkap1	0,10274055	nonSig
				Atp5k	0,01467537	Sig	Yes		Nlgn1	0,10274055	nonSig
				Dync1h1	0,01467537	Sig	No		Egfr	0,10475783	nonSig
				Pdcd6ip	0,01539827	Sig	No		Dlgap1	0,10879822	nonSig
				Hsp90aa1	0,01615176	Sig	No		Rpl26	0,10879822	nonSig
				Rpl13a	0,01774279	Sig	No		Afdn	0,11085376	nonSig

				Selenop	0,01867821	Sig	No		Dclk1	0,12259396	nonSig
				Flot1	0,02050144	Sig	No		Dmtf1	0,12259396	nonSig
				Agt	0,0206002	Sig	No		Rpl3	0,129903	nonSig
				Grin2b	0,02105035	Sig	No		Gnb2	0,13214973	nonSig
				Coa3	0,02130126	Sig	Yes		Rbbp7	0,14141493	nonSig
				Fmr1	0,02130126	Sig	No		Rps10	0,14418425	nonSig
				Pmel	0,02130126	Sig	No		Comt	0,15174388	nonSig
				App	0,02148234	Sig	No		Mbnl3	0,15474972	nonSig
				Dhx9	0,02148234	Sig	No		Ctnna1	0,15927224	nonSig
				Egr4	0,02148234	Sig	No		Cdc42	0,16141239	nonSig
				Rpl11	0,02148234	Sig	No		Slc18a2	0,16566608	nonSig
				Kdm6a	0,0241584	Sig	No		Nos2	0,16583337	nonSig
				Cnbp	0,02444993	Sig	No		Med1	0,16699722	nonSig
				Mrpl36	0,02444993	Sig	No		Chd3	0,17192043	nonSig
				Oca2	0,02444993	Sig	No		Epb41l2	0,17740252	nonSig
				Polr1c	0,02444993	Sig	No		Hdac6	0,17740252	nonSig
				Unc50	0,02444993	Sig	No		Hgs	0,17740252	nonSig

				Eno1	0,02776087	Sig	No		Spata16	0,17740252	nonSig
				Dlg4	0,02827283	Sig	No		Jarid2	0,18432606	nonSig
				Tomm20	0,02862085	Sig	Yes		Tuba1b	0,18432606	nonSig
				Adam10	0,03736972	Sig	No		Actl6b	0,19896757	nonSig
				Glo1	0,03814856	Sig	No		Dnm1	0,19896757	nonSig
				Pdcd5	0,03814856	Sig	No		Eif5b	0,20766837	nonSig
				Bag4	0,04888793	Sig	No		Gad1	0,22233724	nonSig
				Myo18a	0,04888793	Sig	No		Polr2f	0,22233724	nonSig
				Ap3b1	0,05329712	nonSig	No		Krtcap2	0,23018104	nonSig
				Dctn1	0,05329712	nonSig	No		Ckm	0,23804429	nonSig
				Dynll2	0,05329712	nonSig	No		Rab7	0,24524819	nonSig
				Gpx4	0,05329712	nonSig	No		Rps11	0,24524819	nonSig
				Rpl28	0,05329712	nonSig	No		Eif4g1	0,25083805	nonSig
				Rpl38	0,05329712	nonSig	No		Ndufa2	0,25083805	nonSig
				Ank1	0,05415493	nonSig	No		Pts	0,2637476	nonSig
				Psma7	0,05415493	nonSig	No		Eef2	0,26544577	nonSig
				Naca	0,05855498	nonSig	No		Psmb6	0,26544577	nonSig

				Pigz	0,06422793	nonSig	No		Rbx1	0,27326082	nonSig
				Tcp1	0,06422793	nonSig	No		Arfgap1	0,27923143	nonSig
				Hnrnpu	0,07707115	nonSig	No		H3c7	0,27923143	nonSig
				Pick1	0,08360791	nonSig	No		Rho	0,28663023	nonSig
				Cask	0,08491156	nonSig	No		Kdr	0,29774388	nonSig
				Arf1	0,09671603	nonSig	No		Sumo3	0,29774388	nonSig
				Rpl8	0,09671603	nonSig	No		Actl6a	0,30469197	nonSig
				Aifm1	0,10274055	nonSig	Yes		Hsp90ab1	0,30469197	nonSig
				Mapkap1	0,10274055	nonSig	No		Ap2b1	0,31069862	nonSig
				Nlgn1	0,10274055	nonSig	No		Dut	0,31069862	nonSig
				Egfr	0,10475783	nonSig	No		Lypd6b	0,31069862	nonSig
				Dlgap1	0,10879822	nonSig	No		Rims2	0,31069862	nonSig
				Rpl26	0,10879822	nonSig	No		Tmem126b	0,32821047	nonSig
				Afdn	0,11085376	nonSig	No		Fus	0,33541891	nonSig
				Dclk1	0,12259396	nonSig	No		Ola1	0,34519254	nonSig
				Dmtf1	0,12259396	nonSig	No		Anxa1	0,36521994	nonSig
				Rpl3	0,129903	nonSig	No		Grin2a	0,36521994	nonSig

				Gnb2	0,13214973	nonSig	No		Rps3	0,36521994	nonSig
				Rbbp7	0,14141493	nonSig	No		Dnmt1	0,36801677	nonSig
				Rps10	0,14418425	nonSig	No		Egr2	0,39307541	nonSig
				Comt	0,15174388	nonSig	No		Atp2b1	0,39311048	nonSig
				Mbnl3	0,15474972	nonSig	No		Rpl9	0,40956359	nonSig
				Ctnna1	0,15927224	nonSig	No		Aurkc	0,42415743	nonSig
				Cdc42	0,16141239	nonSig	No		Dcx	0,42415743	nonSig
				Slc18a2	0,16566608	nonSig	No		Dlk1	0,42453431	nonSig
				Nos2	0,16583337	nonSig	No		Prkaca	0,43654208	nonSig
				Med1	0,16699722	nonSig	No		Cdh15	0,43678217	nonSig
				Chd3	0,17192043	nonSig	No		Hnrnpk	0,43707076	nonSig
				Epb41l2	0,17740252	nonSig	No		Jup	0,45929273	nonSig
				Hdac6	0,17740252	nonSig	No		Aldoc	0,46212992	nonSig
				Hgs	0,17740252	nonSig	No		Ly6h	0,46212992	nonSig
				Spata16	0,17740252	nonSig	No		U2af2	0,47655917	nonSig
				Jarid2	0,18432606	nonSig	No		Ppp1ca	0,51250114	nonSig
				Tuba1b	0,18432606	nonSig	No		Ncstn	0,54766536	nonSig

				Vdac1	0,19203126	nonSig	Yes		Jund	0,55659298	nonSig
				Actl6b	0,19896757	nonSig	No		Slc45a2	0,55659298	nonSig
				Dnm1	0,19896757	nonSig	No		Txnrd1	0,55659298	nonSig
				Eif5b	0,20766837	nonSig	No		Eif4e	0,57234746	nonSig
				Gad1	0,22233724	nonSig	No		Nek6	0,57828133	nonSig
				Polr2f	0,22233724	nonSig	No		Numa1	0,5790069	nonSig
				Krtcap2	0,23018104	nonSig	No		Atf2	0,58563458	nonSig
				Ckm	0,23804429	nonSig	No		Psmd14	0,58686505	nonSig
				Rab7	0,24524819	nonSig	No		Flna	0,6125284	nonSig
				Rps11	0,24524819	nonSig	No		Gja1	0,6125284	nonSig
				Cox5b	0,25083805	nonSig	Yes		Gna12	0,61800906	nonSig
				Eif4g1	0,25083805	nonSig	No		Arhgap35	0,63088109	nonSig
				Ndufa2	0,25083805	nonSig	No		Ier3	0,64125963	nonSig
				Pts	0,2637476	nonSig	No		Ngfr	0,64132998	nonSig
				Eef2	0,26544577	nonSig	No		Gnaq	0,64329692	nonSig
				Psmb6	0,26544577	nonSig	No		Ctnnb1	0,65884627	nonSig
				Rbx1	0,27326082	nonSig	No		Dnm2	0,65884627	nonSig

				Arfgap1	0,27923143	nonSig	No		Grb2	0,65884627	nonSig
				Atp5l	0,27923143	nonSig	Yes		Tsg101	0,66462203	nonSig
				H3c7	0,27923143	nonSig	No		Eif1a	0,67399803	nonSig
				Rho	0,28663023	nonSig	No		Arhgef6	0,68315457	nonSig
				Kdr	0,29774388	nonSig	No		Mlst8	0,69209598	nonSig
				Sumo3	0,29774388	nonSig	No		Eif4a1	0,70082646	nonSig
				Actl6a	0,30469197	nonSig	No		Ikbke	0,70779801	nonSig
				Hsp90ab1	0,30469197	nonSig	No		Men1	0,70779801	nonSig
				Ap2b1	0,31069862	nonSig	No		Sgk1	0,71587192	nonSig
				Dut	0,31069862	nonSig	No		Nup214	0,73562622	nonSig
				Lypd6b	0,31069862	nonSig	No		Evl	0,74145715	nonSig
				Rims2	0,31069862	nonSig	No		Afp	0,76822731	nonSig
				Mfn1	0,32821047	nonSig	Yes		Ccnb1	0,77108796	nonSig
				Tmem126b	0,32821047	nonSig	No		Apaf1	0,7729084	nonSig
				Fus	0,33541891	nonSig	No		Rack1	0,77975804	nonSig
				Ola1	0,34519254	nonSig	No		Ehmt2	0,78643746	nonSig
				Anxa1	0,36521994	nonSig	No		Alb	0,81023633	nonSig

				Grin2a	0,36521994	nonSig	No		Chuk	0,81023633	nonSig
				Rps3	0,36521994	nonSig	No		Dlx5	0,81983618	nonSig
				Dnmt1	0,36801677	nonSig	No		Mecp2	0,81983618	nonSig
				Egr2	0,39307541	nonSig	No		Il6	0,83916944	nonSig
				Atp2b1	0,39311048	nonSig	No		Plk1	0,84398666	nonSig
				Rpl9	0,40956359	nonSig	No		Brd2	0,84955705	nonSig
				Aurkc	0,42415743	nonSig	No		Ptk6	0,85207977	nonSig
				Dcx	0,42415743	nonSig	No		Ranbp2	0,85207977	nonSig
				Dlk1	0,42453431	nonSig	No		Calml3	0,86172156	nonSig
				Prkaca	0,43654208	nonSig	No		Pdgfa	0,86172156	nonSig
				Cdh15	0,43678217	nonSig	No		Hsf1	0,88278325	nonSig
				Hnrnpk	0,43707076	nonSig	No		Smurf1	0,89531918	nonSig
				Mtg1	0,4504575	nonSig	Yes		Plg	0,91816949	nonSig
				Jup	0,45929273	nonSig	No		Fgfr2	0,91879546	nonSig
				Aldoc	0,46212992	nonSig	No		Gnao1	0,91879546	nonSig
				Ly6h	0,46212992	nonSig	No		Smarca1	0,93412377	nonSig
				U2af2	0,47655917	nonSig	No		Epha2	0,94284555	nonSig

				Bcl2	0,51250114	nonSig	Yes		Hdac4	0,94572298	nonSig
				Ppp1ca	0,51250114	nonSig	No		Klf6	0,94582683	nonSig
				Ncstn	0,54766536	nonSig	No		Ntrk2	0,94582683	nonSig
				Jund	0,55659298	nonSig	No		Creb1	0,95713811	nonSig
				Slc45a2	0,55659298	nonSig	No		Ins2	0,97024957	nonSig
				Txnrd1	0,55659298	nonSig	No		Akt1	0,97655392	nonSig
				Eif4e	0,57234746	nonSig	No		Cbl	0,97655392	nonSig
				Nek6	0,57828133	nonSig	No		Bmpr2	0,99597754	nonSig
				Numa1	0,5790069	nonSig	No		Abce1	1	nonSig
				Atf2	0,58563458	nonSig	No		Abl1	1	nonSig
				Bcl2l1	0,58563458	nonSig	Yes		Acsf2	1	nonSig
				Psmd14	0,58686505	nonSig	No		Actr10	1	nonSig
				Flna	0,6125284	nonSig	No		Als2	1	nonSig
				Gja1	0,6125284	nonSig	No		Ar	1	nonSig
				Gna12	0,61800906	nonSig	No		Atf3	1	nonSig
				Arhgap35	0,63088109	nonSig	No		Atf4	1	nonSig
				Ier3	0,64125963	nonSig	No		Atm	1	nonSig
				Ngfr	0,64132998	nonSig	No		Atp1a2	1	nonSig

				Gnaq	0,64329692	nonSig	No		Aurka	1	nonSig
				Ctnnb1	0,65884627	nonSig	No		Bad	1	nonSig
				Dnm2	0,65884627	nonSig	No		Braf	1	nonSig
				Grb2	0,65884627	nonSig	No		Brca1	1	nonSig
				Tsg101	0,66462203	nonSig	No		Casp3	1	nonSig
				Eif1a	0,67399803	nonSig	No		Ccdc85b	1	nonSig
				Arhgef6	0,68315457	nonSig	No		Ccl12	1	nonSig
				Mlst8	0,69209598	nonSig	No		Cd44	1	nonSig
				Eif4a1	0,70082646	nonSig	No		Cdk1	1	nonSig
				Ikbke	0,70779801	nonSig	No		Cdk2	1	nonSig
				Men1	0,70779801	nonSig	No		Cdk5	1	nonSig
				Sgk1	0,71587192	nonSig	No		Celf3	1	nonSig
				Nup214	0,73562622	nonSig	No		Chmp2b	1	nonSig
				Evl	0,74145715	nonSig	No		Chmp3	1	nonSig
				Afp	0,76822731	nonSig	No		Ckap5	1	nonSig
				Ccnb1	0,77108796	nonSig	No		Coa5	1	nonSig
				Apaf1	0,7729084	nonSig	No		Cope	1	nonSig

				Rack1	0,77975804	nonSig	No		Crebbp	1	nonSig
				Ehmt2	0,78643746	nonSig	No		Csnk2a1	1	nonSig
				Alb	0,81023633	nonSig	No		Ctcf	1	nonSig
				Chuk	0,81023633	nonSig	No		Ctcfl	1	nonSig
				Dlx5	0,81983618	nonSig	No		Cul7	1	nonSig
				Mecp2	0,81983618	nonSig	No		Dnajc13	1	nonSig
				Bax	0,83759864	nonSig	Yes		E2f1	1	nonSig
				Il6	0,83916944	nonSig	No		E2f4	1	nonSig
				Plk1	0,84398666	nonSig	No		Egr1	1	nonSig
				Brd2	0,84955705	nonSig	No		Eif2ak2	1	nonSig
				Ptk6	0,85207977	nonSig	No		Ep300	1	nonSig
				Ranbp2	0,85207977	nonSig	No		Erbb2	1	nonSig
				Calml3	0,86172156	nonSig	No		Esr1	1	nonSig
				Pdgfa	0,86172156	nonSig	No		Fndc9	1	nonSig
				Hsf1	0,88278325	nonSig	No		Fos	1	nonSig
				Lyn	0,88856613	nonSig	Yes		Foxa2	1	nonSig
				Smurf1	0,89531918	nonSig	No		Foxp3	1	nonSig

				Plg	0,91816949	nonSig	No		Fubp3	1	nonSig
				Fgfr2	0,91879546	nonSig	No		Gabpa	1	nonSig
				Gnao1	0,91879546	nonSig	No		Gata2	1	nonSig
				Smarca1	0,93412377	nonSig	No		Gata3	1	nonSig
				Epha2	0,94284555	nonSig	No		Gnb1	1	nonSig
				Hdac4	0,94572298	nonSig	No		Grpel1	1	nonSig
				Klf6	0,94582683	nonSig	No		H2ax	1	nonSig
				Ntrk2	0,94582683	nonSig	No		H3f3a	1	nonSig
				Creb1	0,95713811	nonSig	No		Hdac1	1	nonSig
				Ins2	0,97024957	nonSig	No		Hif1a	1	nonSig
				Akt1	0,97655392	nonSig	No		Jun	1	nonSig
				Cbl	0,97655392	nonSig	No		Kif1a	1	nonSig
				Bmpr2	0,99597754	nonSig	No		Lat	1	nonSig
				Abce1	1	nonSig	No		Luzp2	1	nonSig
				Abl1	1	nonSig	No		Map3k7	1	nonSig
				Acad9	1	nonSig	Yes		Mapk1	1	nonSig
				Acsf2	1	nonSig	No		Mapk3	1	nonSig
				Actr10	1	nonSig	No		Mapk8	1	nonSig
				Als2	1	nonSig	No		Mitf	1	nonSig
				Ar	1	nonSig	No		Mrpl11	1	nonSig
				Atf3	1	nonSig	No		Mrpl12	1	nonSig
				Atf4	1	nonSig	No		Mrpl13	1	nonSig

				Atm	1	nonSig	No		Mrpl16	1	nonSig
				Atp1a2	1	nonSig	No		Mrpl2	1	nonSig
				Aurka	1	nonSig	No		Mtor	1	nonSig
				Bad	1	nonSig	No		Myb	1	nonSig
				Braf	1	nonSig	No		Myc	1	nonSig
				Brca1	1	nonSig	No		Nanog	1	nonSig
				Casp3	1	nonSig	No		Nap1l5	1	nonSig
				Ccdc85b	1	nonSig	No		Nfkbia	1	nonSig
				Ccl12	1	nonSig	No		Nfya	1	nonSig
				Cd44	1	nonSig	No		Nfyb	1	nonSig
				Cdk1	1	nonSig	No		Ngf	1	nonSig
				Cdk2	1	nonSig	No		Nkain2	1	nonSig
				Cdk5	1	nonSig	No		Notch1	1	nonSig
				Celf3	1	nonSig	No		Npm1	1	nonSig
				Chmp2b	1	nonSig	No		Nrf1	1	nonSig
				Chmp3	1	nonSig	No		Nsmce1	1	nonSig
				Ckap5	1	nonSig	No		Pak1	1	nonSig
				Clpb	1	nonSig	Yes		Pax6	1	nonSig
				Coa5	1	nonSig	No		Plcg1	1	nonSig
				Cope	1	nonSig	No		Pten	1	nonSig
				Crebbp	1	nonSig	No		Rac1	1	nonSig
				Csnk2a1	1	nonSig	No		Ret	1	nonSig
				Ctcf	1	nonSig	No		Rhoa	1	nonSig
				Ctcfl	1	nonSig	No		Scly	1	nonSig
				Cul7	1	nonSig	No		Slc50a1	1	nonSig
				Dnajc13	1	nonSig	No		Smad3	1	nonSig
				E2f1	1	nonSig	No		Smarca4	1	nonSig
				E2f4	1	nonSig	No		Smarcb1	1	nonSig
				Egr1	1	nonSig	No		Snx4	1	nonSig
				Eif2ak2	1	nonSig	No		Sox2	1	nonSig
				Ep300	1	nonSig	No		Sox9	1	nonSig
				Erbb2	1	nonSig	No		Srf	1	nonSig
				Esr1	1	nonSig	No		St18	1	nonSig
				Fndc9	1	nonSig	No		Syk	1	nonSig
				Fos	1	nonSig	No		Tbp	1	nonSig

				Foxa2	1	nonSig	No		Tgfb1	1	nonSig
				Foxp3	1	nonSig	No		Tnf	1	nonSig
				Fubp3	1	nonSig	No		Tp53	1	nonSig
				Gabpa	1	nonSig	No		Zap70	1	nonSig
				Gata2	1	nonSig	No		Zbtb7a	1	nonSig
				Gata3	1	nonSig	No				
				Gnb1	1	nonSig	No				
				Grpel1	1	nonSig	No				
				H2ax	1	nonSig	No				
				H3f3a	1	nonSig	No				
				Hdac1	1	nonSig	No				
				Hif1a	1	nonSig	No				
				Jun	1	nonSig	No				
				Kat2a	1	nonSig	Yes				
				Kif1a	1	nonSig	No				
				Lat	1	nonSig	No				
				Luzp2	1	nonSig	No				
				Map3k7	1	nonSig	No				
				Mapk1	1	nonSig	No				
				Mapk3	1	nonSig	No				
				Mapk8	1	nonSig	No				
				Mitf	1	nonSig	No				
				Mrpl11	1	nonSig	No				
				Mrpl12	1	nonSig	No				
				Mrpl13	1	nonSig	No				
				Mrpl16	1	nonSig	No				
				Mrpl2	1	nonSig	No				
				Mtor	1	nonSig	No				
				Myb	1	nonSig	No				
				Myc	1	nonSig	No				
				Nanog	1	nonSig	No				

				Nap1l5	1	nonSig	No				
				Nfkbia	1	nonSig	No				
				Nfya	1	nonSig	No				
				Nfyb	1	nonSig	No				
				Ngf	1	nonSig	No				
				Nkain2	1	nonSig	No				
				Notch1	1	nonSig	No				
				Npm1	1	nonSig	No				
				Nrf1	1	nonSig	No				
				Nsmce1	1	nonSig	No				
				Pak1	1	nonSig	No				
				Pax6	1	nonSig	No				
				Plcg1	1	nonSig	No				
				Pten	1	nonSig	No				
				Rac1	1	nonSig	No				
				Ret	1	nonSig	No				
				Rhoa	1	nonSig	No				
				Scly	1	nonSig	No				
				Slc50a1	1	nonSig	No				
				Smad3	1	nonSig	No				
				Smarca4	1	nonSig	No				
				Smarcb1	1	nonSig	No				
				Snx4	1	nonSig	No				
				Sox2	1	nonSig	No				
				Sox9	1	nonSig	No				
				Src	1	nonSig	Yes				
				Srf	1	nonSig	No				
				St18	1	nonSig	No				
				Stat3	1	nonSig	Yes				
				Syk	1	nonSig	No				
				Tbp	1	nonSig	No				
				Tgfb1	1	nonSig	No				
				Tnf	1	nonSig	No				
				Tp53	1	nonSig	No				
				Zap70	1	nonSig	No				
				Zbtb7a	1	nonSig	No				
